# Crude phosphorylation mixtures containing racemic lipid amphiphiles self-assemble to give stable primitive compartments

**DOI:** 10.1038/s41598-017-18053-y

**Published:** 2017-12-22

**Authors:** Dimitri Fayolle, Emiliano Altamura, Alice D’Onofrio, Warren Madanamothoo, Bernard Fenet, Fabio Mavelli, René Buchet, Pasquale Stano, Michele Fiore, Peter Strazewski

**Affiliations:** 1 Institut de Chimie et Biochimie Moléculaires et Supramoléculaires, Université de Lyon, Claude Bernard Lyon 1, 43 bvd du 11 Novembre 1918, F–69622 Villeurbanne Cedex, France; 2 Department of Chemistry, University of Bari, Via E. Orabona 4, I–70125 Bari, Italy; 3 Biological and Environmental Science and Technology Department, University of Salento, Ecotekne, I–73100 Lecce, Italy

## Abstract

It is an open question how the chemical structure of prebiotic vesicle-forming amphiphiles complexified to produce robust primitive compartments that could safely host foreign molecules. Previous work suggests that comparingly labile vesicles composed of plausibly prebiotic fatty acids were eventually chemically transformed with glycerol and a suitable phosphate source into phospholipids that would form robust vesicles. Here we show that phosphatidic acid (PA) and phosphatidylethanolamine (PE) lipids can be obtained from racemic dioleoyl glycerol under plausibly prebiotic phosphorylation conditions. Upon *in situ* hydration of the crude phosphorylation mixtures only those that contained *rac-*DOPA (not *rac-*DOPE) generated stable giant vesicles that were capable of encapsulating water-soluble probes, as evidenced by confocal microscopy and flow cytometry. Chemical reaction side-products (identified by IR and MS and quantified by ^1^H NMR) acted as co-surfactants and facilitated vesicle formation. To mimic the compositional variation of such primitive lipid mixtures, self-assembly of a combinatorial set of the above amphiphiles was tested, revealing that too high dioleoyl glycerol contents inhibited vesicle formation. We conclude that a decisive driving force for the gradual transition from unstable fatty acid vesicles to robust diacylglyceryl phosphate vesicles was to avoid the accumulation of unphosphorylated diacylglycerols in primitive vesicle membranes.

## Introduction

The spontaneous supramolecular self-assembly of amphiphiles to give vesicles is a powerful thermodynamic drive for the emergence of primitive cell-like compartments on the early Earth^1–7^. Fatty acid vesicles are plausible models of primitive cells^8,9^ but contemporary cell membranes are based on mixtures of phospholipids, glycolipids and proteins. This elicits the questions about the stepwise transition from very early achiral or racemic amphiphiles to enantiopure glycerophospholipids.

It has been argued that the complexification and the compositional evolution of primitive membranes was subjected not only to chemical rules (which chemical transformation is possible?) but also to a sort of supramolecular selection based on the ‘performance’ of the resulting membranes and whole vesicles – e.g., low critical aggregation (micelle, vesicle) concentration, membrane permeability, capture and retention of non-lipidic organic molecules, membrane growth and vesicle division upon the addition of membrane components, vesicle stability at high magnesium and calcium ions concentrations^8,9^. Although chemistry suggests several plausible pathways for the stepwise transformation of simple amphiphiles into complex lipids^10–15^, the experimental verifications of the existence, stability, and properties of vesicles composed of plausibly primitive amphiphile mixtures are still limited^9,16–21^.

Here we report on primitive membrane systems that originate from crude lipid phosphorylation mixtures. Our starting point is a non-phosphorylated diacylglycerol racemate. Glycerol is a reduced formose C_3_ reaction product^22^ and *n-*alkanoic acids are Fischer-Tropsch-type reactions products^23^. Acyl glycerol esters are formed from the above compounds under so-called “hydrothermal conditions” (100–300 °C)^10–12^. Such conditions are too harsh for phosphorylation reactions to be efficient. It is worth noting that our approach deals with the phosphorylation of *n*-alkanoic glyceryl esters rather than with the acylation of glycerol phosphate, which could be an alternative prebiotic route^24^. As phosphate source we have used inorganic phosphate (P_i_) and 2-aminoethyl phosphate (AEP) for assessing the formation of racemic phosphatidic acid (PA) monoesters and, respectively, phosphatidylethanolamine (PE) diesters. Urea or cyanamide have been employed as dehydrating agents. Cyanamide is a plausibly prebiotic condensing agent^13,15^ already used for the synthesis of glycerol phosphate, of some phospholipids and the concomitant growing of oligopeptides and nucleosides^25–29^. Our approach considerably differs from (and is therefore complementary to) the pioneering experiments carried out for the formation of several phospholipids^10,14,25,26,30,31^.

## Results

### Prebiotic phosphorylation of racemic dioleoyl glycerol to racemic DOPA and DOPE

**Figure 1:**
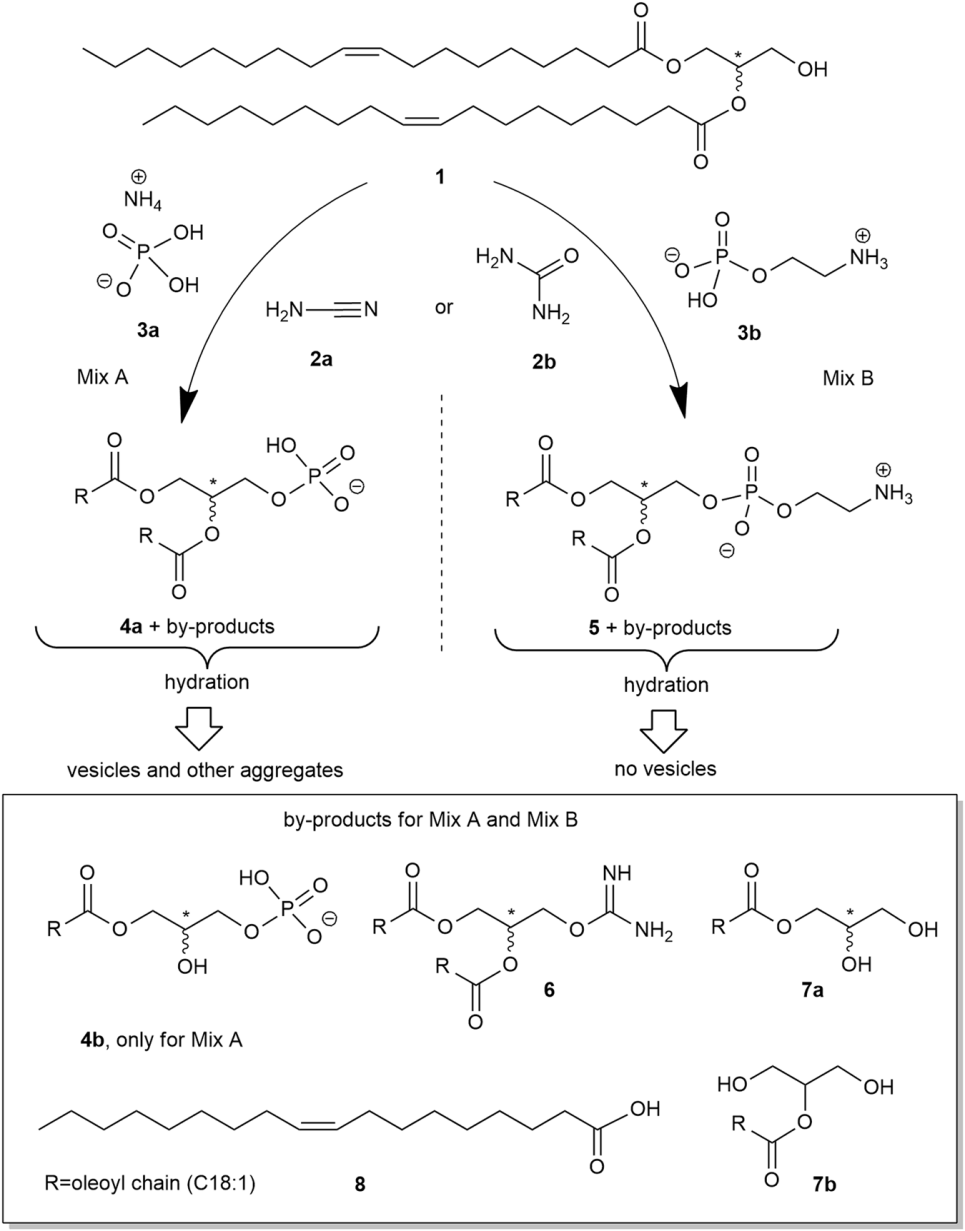
Summary of the two investigated phosphorylation reactions and structures of the obtained compounds (R = oleoyl chain), followed by the hydration of the crude reaction mixtures: *rac*-DOG (**1**) in the presence of cyanamide (**2a**) or urea (**2b**) undergoes two phosphorylation reactions with ammonium dihydrogen phosphate (**3a**) and with 2-aminoethyl phosphate (**3b**) to give *rac*-DOPA (**4a**) and, respectively, *rac*-DOPE (**5**). Five by-products were identified in the Mix A: *rac-*MOPA (**4b**), isourea **6**, *rac*-MOG (**7a**), *sec-*MOG (**7b**) and oleic acid (OA, **8**) and four (**6**, **7a**,**b** and **8**) in the Mix B. Compounds **1**, **4a**, **4b**, **5** and **7a** are 1:1 mixtures of two enantiomers: *rac*-DOG (**1**) = 1,2-dioleyl-*sn*-glycerol + 2,3-dioleoyl-*sn*-glycerol; *rac*-DOPA (**4a**) = 1,2-dioleoyl-*sn*-glycero-3-phosphate + 2,3-dioleoyl-*sn*-glycero-1-phosphate; *rac*-MOPA (**4b**) = 1-oleoyl-*sn*-glycero-3-phosphate + 3-oleoyl-*sn*-glycero-1-phosphate; *rac*-DOPE (**5**) = 1,2-dioleoyl-*sn*-glycero-3-phosphoethanolamine + 2,3-dioleoyl-*sn*-glycero-1-phosphoethanolamine; *rac*-MOG (**7a**) = 2,3-*sn*-dihydroxypropyl-1-oleate + 1,2-*sn*-dihydroxypropyl-3-oleate.

Racemic dioleoylglycerol^32^ (*rac*-DOG, **1**, Fig. 1) was submitted to phosphorylation in the presence of cyanamide (**2a**) or urea (**2b**) and ammonium dihydrogen P_i_ (**3a**) or AEP (**3b**) to obtain the corresponding racemic dioleoyl-PA (**4**, *rac*-DOPA) and, respectively, dioleoyl-PE (**5**, *rac*-DOPE). All reactions were carried out for 24 to 48 hours at 80 °C in rapidly evaporating water, as well as under neat conditions^33^. The structures of **4a**, **5** and the by-products **4b** (*rac-*MOPA)**, 6**, **7a** (*rac*-MOG), **7b** (*sec*-MOG) and **8** (OA) are consistent with ^1^H NMR, IR and ESI-MS analyses of the crude extracted mixtures and by comparison with commercial or synthetic samples (Figures S19–S38)^34^. Small, not quantified amounts of the isourea **6** were identified by ESI-MS and formed only when **2a** was used as the activator. We refer to “Mix A” for mixtures containing *rac*-DOPA (**4a**) while “Mix B” contain *rac*-DOPE (**5**). Crude mixtures A and B were analyzed by ^1^H NMR, IR and ESI-MS. The products **4a** or **5** appeared together with **7a**, **8** and traces of **7b**. We summarize the detailed analysis described below as follows: Mix A contained **8**, **7a** + **7b**, **1** and **4a** + **4b** in comparable amounts = 3.3: 1.6: 0.9: 4.2 (conversion of **1** = 65.7%) and Mix B contained molar ratios of **8**, **7a** + **7b**, **1** and **5** = 2.0: 2.5: 2.4: 3.1 (conversion of **1** = 75%). The composition of the two crude mixtures Mix A and Mix B is quite intriguing (Table 1, upper part). These mixtures realistically model the molecular lipid diversity in primitive reacting environments. They represent plausible compositions of protocell membranes that emerged from the underlying chemistry. In particular, the lipids of Mix A and Mix B can be considered as members of a primitive chemical route, including fatty acids, monoacylglycerols, diacylglycerols and the simplest glycerophospholipids.

**Table 1:** Vesicles prepared from hydration of plausibly prebiotic lipid mixtures.

Crude mixtures	OA	MOG	DOG	MOPA	DOPA	DOPE	Vesicles?^c^
	8	7a^a^ + 7b	1^a^	4b^a^	4a^a^	5^a^	
	**Obtained molar ratios**						
Mix A	3.3	1.6	0.9	0.4^b^	3.8		mostly
Mix B	2.0	2.5	2.4			3.1	no
**Reconstituted mixtures**	**OA**	**MOG**	**DOG**		**DOPA**	**DOPE**	**Vesicles?** ^c^
	**8**	**7a** ^a^	**1** ^a^				
	**Added molar ratios**						
M1	4	1	2		3		mostly
M2	4	1	2			3	no
M3	1	1	2		6		mostly
M4	1	1	2			6	no
N1	8	2					mostly
N2	6	2	2				no
N3	4	2	2		2		mostly
N4	2	2	2		4		partially
N5		2	2		6		partially
N6			2		8		partially
N7					10		yes

^a^Racemic compounds; ^b^Estimated from TOCSY integrals; ^c^All mixtures have been hydrated with 25 mM Tris-HCl pH 7.5 containing 200 mM sucrose, except Mix B, M2 and M4 which have been hydrated with 200 mM Na-bicine pH 8.5 containing 200 mM sucrose. An isotonic sucrose/glucose density difference was used for the reliable microscopic imaging and counting of GVs; see supplementary documentation, paragraphs I.1 and I.2 for details on the preparation of GVs from Mix A and B and for N1–N7.

**Figure 2:**
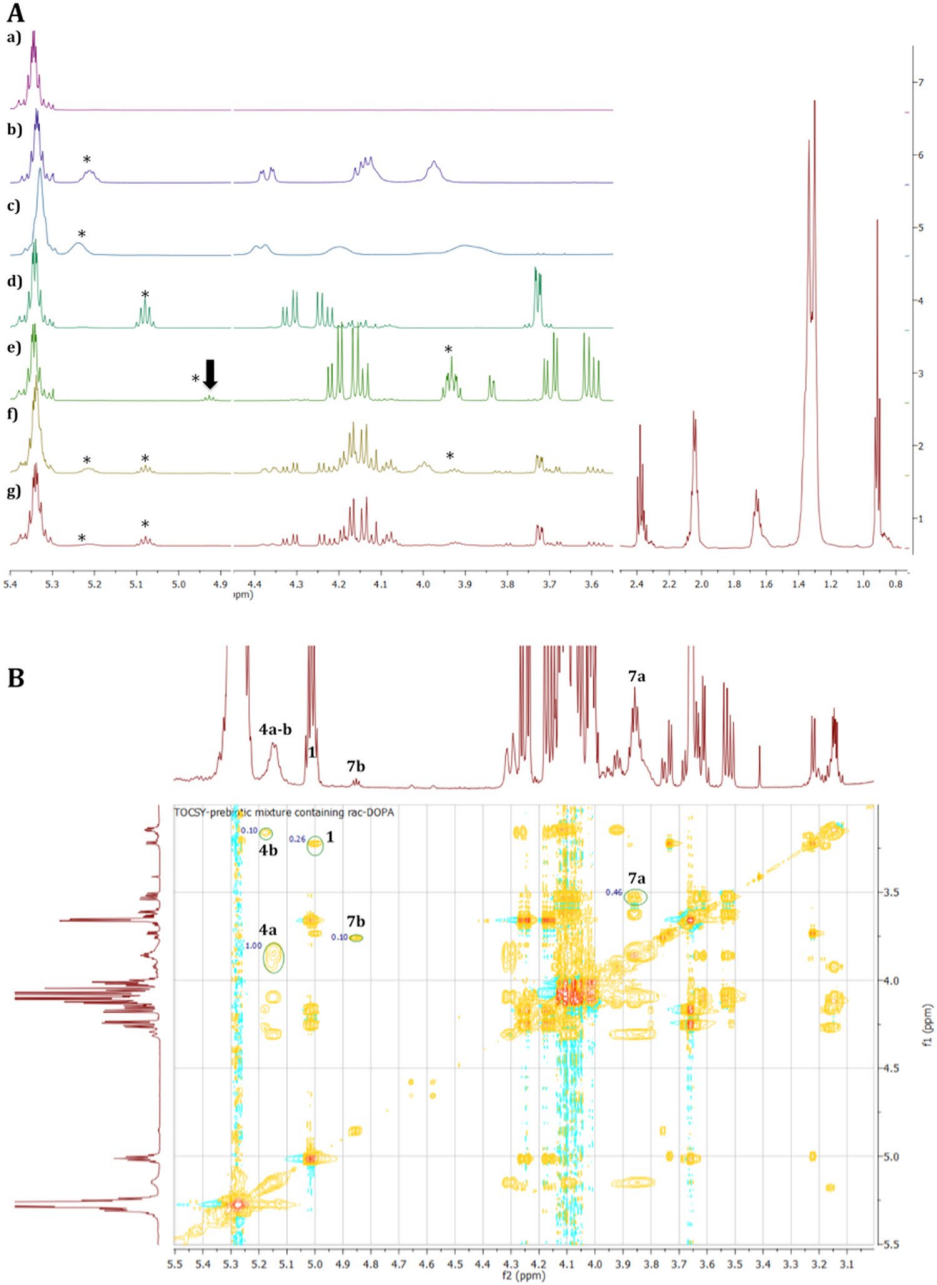
(**A**) ^1^HNMR recorded at 500 MHz in CDCl_3_; chemical shift region for protons bound to carbon atoms in α-position to an oxygen atom or part of a –C=C– double bond, i.e. glycerol –CH– and –CH_2_– groups and *Z*–CH=CH– of the oleoyl chains. **a**) commercial oleic acid; **b**) Commercial DOPE; **c**) Commercial DOPA; **d**) *rac*-DOG (**1**); **e**) *rac*-MOG (**7a**) that contains traces of *sec*-MOG (**7b**, indicated by an arrow); **f**) Mix B (containing **1**, **5**, **7a**, **7b** and **8**); **g**) Mix A (containing **1**, **4a**, **4b**, **7a**, **7b** and **8**); Asterisks indicate the secondary hydrogen atom of the glycerol backbone. Small amounts of adduct **6** were identified only by ESI-MS and formed only when **2a** was used as an activator. (**B**) TOCSY (500 MHz, CDCl_3_, region 5.5–3.0 ppm) of crude extract of Mix A containing **1**, **4a**, **4b 7a, 7b** and **8**. The green circles represent the 2D integration of the central glycerol backbone CH proton signal. In ^1^H NMR of Mix A (Figure 2**Ag**) the signals of **4a** and **4b** overlap.

We based the quantification of all major side-products on the integration of proton NMR signals of the glycerol backbone in the chemical shift region δ_H_ = 5.28–3.50 ppm (Fig. 2, Table S1, Fig. S1–S3). The aliphatic region, δ_H_ = 0.87 ppm (CH_3_) to 1.27–2.36 (CH_2_) ppm, and that of the *Z* unsaturation at δ_H_ = 5.38–5.31 ppm (CH=CH) were very similar for all these molecules. The approximate amount of not esterified oleic acid was derived from the total integral of the CH=CH signals minus the sum of the integrals of the secondary protons of the glycerol backbone of **4b** and **7a** + **7b** and **7a** + **7b** for Mix A and Mix B, respectively. The obtained mol% value for OA served to quantify the conversion of **1** into **4a** or **5** and into the corresponding monooleins **7a**,**b** and **8**. TOCSY (TOtal Correlated SpectroscopY) and HSQC (Heteronuclear Single Quantum Coherence spectroscopy) experiments confirmed what was attributed in the 1D ^1^H NMR analysis and allowed to estimate the small amount of **4b**. The ratio between **4a** and **4b** was estimated 9:1 (Table S1). Analysis of crude extracts for reaction mixtures that involved **1** for the formation of **4a** or **5** in the presence of **2b** (urea) and **3a** or **3b** have shown the presence of a puzzle of signals that mainly correspond to those molecules observed by ESI-MS analysis containing one or two oleoyl chains. The ^1^H NMR of the crude extracted mixture (Mix A)  has the characteristic signals of the oleoyl chain observed at δ_H_ = 0.87, 1.26, 1.29, 1.61, 2.00, and 2.36 ppm with a typical NMR profile of a fatty acid chain bearing one insaturation (δ_H_ = 5.38–5.31 ppm) (Figures S17 and S18). The signal of the secondary hydrogen atom of the glycerol backbone in *rac*-DOPA (**4a**) was observed at δ_H_ = 5.15 ppm and confirmed by comparison of the signals of commercial DOPA. In the ^1^H NMR of Mix A, the signal of the secondary hydrogen atom of the glycerol backbone in *rac*-MOPA (**4b**) overlap that of **4a** but was observed at δ_H_ = 5.17 ppm in the TOCSY spectra (Figure S3). The residual starting material **1** appears at δ_H_ = 5.08, 4.33–4.20 and 3.73 ppm. Oleic acid (**8**) appears with signals of oleic alkyl chain that overlap with the signals already described for *rac*-DOPA. The corresponding protons of formed *rac*-2,3-dihydroxypropyl oleate (**7a**) and partially masked by those of **1** (δ_H_ = 4.20–4.12 ppm) appear at δ_H_ = 3.92 and 3.68–3.57 ppm. Traces of 1,3-dihydroxypropan-2-yl oleate (**7b**, *sec*-MOG) are visible as a triplet at δ_H_ = 4.92, whereas the other signals are masked by those of **1**, **6** and **7a**. The analysis of ^1^H NMR spectra of crude extracts of mixtures containing **1**, **5**, **7a** and **8** (Mix B) gave similar results. Gratifyingly, the signals of the glycerol backbone at δ_H_ = 5.21, 4.39–4.36 and 4.10 ppm were distinct from the methylene signals of the phosphoethanolamine residue (–OCH_2_CH_2_NH_3_^+^) found at δ_H_ = 3.98 and 3.12 ppm.

Confirmation of the NMR spectroscopic assignments was obtained by mass spectrometry, where *rac*-DOPA (**4a**) was detected as *m*/*z* 701 [M + H]^+^, 723 [M + Na]^+^ and 699 [M–H]^−^, mono-acyl glycerol phosphate **4b** as *m*/*z* 435 [M–H]^–^. Compound **6** was detected as *m*/*z* 663 [M + H]^+^ and 685 [M + Na]^+^. Its presence was observed only when the reactions were carried out in presence of 2a. The two unphosphorylated mono-acylglycerols **7a** or **7b** were detected as *m*/*z* 339 [M-H_2_O]^+^, and oleic acid (**8**) as *m*/*z* 282 [M + H]^+^. Starting material **1** was detected as *m*/*z* 621 [M + H]^+^, 643 [M + Na]^+^, 603 [M-H_2_O]^+^ and in the negative ion mode 1 as *m*/*z* 655 [M+Cl]^−^ and 665 [M+HCOO]^−^. *Rac*-DOPE (**5**) was detected as *m*/*z* 744 [M + H]^+^ and 742 [M-H]^−^. Notably, **5** was detected together with compounds **1**, **7a** or **7b** and **8**. Again, compound **6** was detected as *m*/*z* 663 [M + H]^+^. Its presence was observed only when the reactions conditions used for obtaining **5** were carried out in presence of **2a** instead of **2b**.

###  Vibrational analysis of crude mixtures containing racemic phospholipids

**Figure 3:**
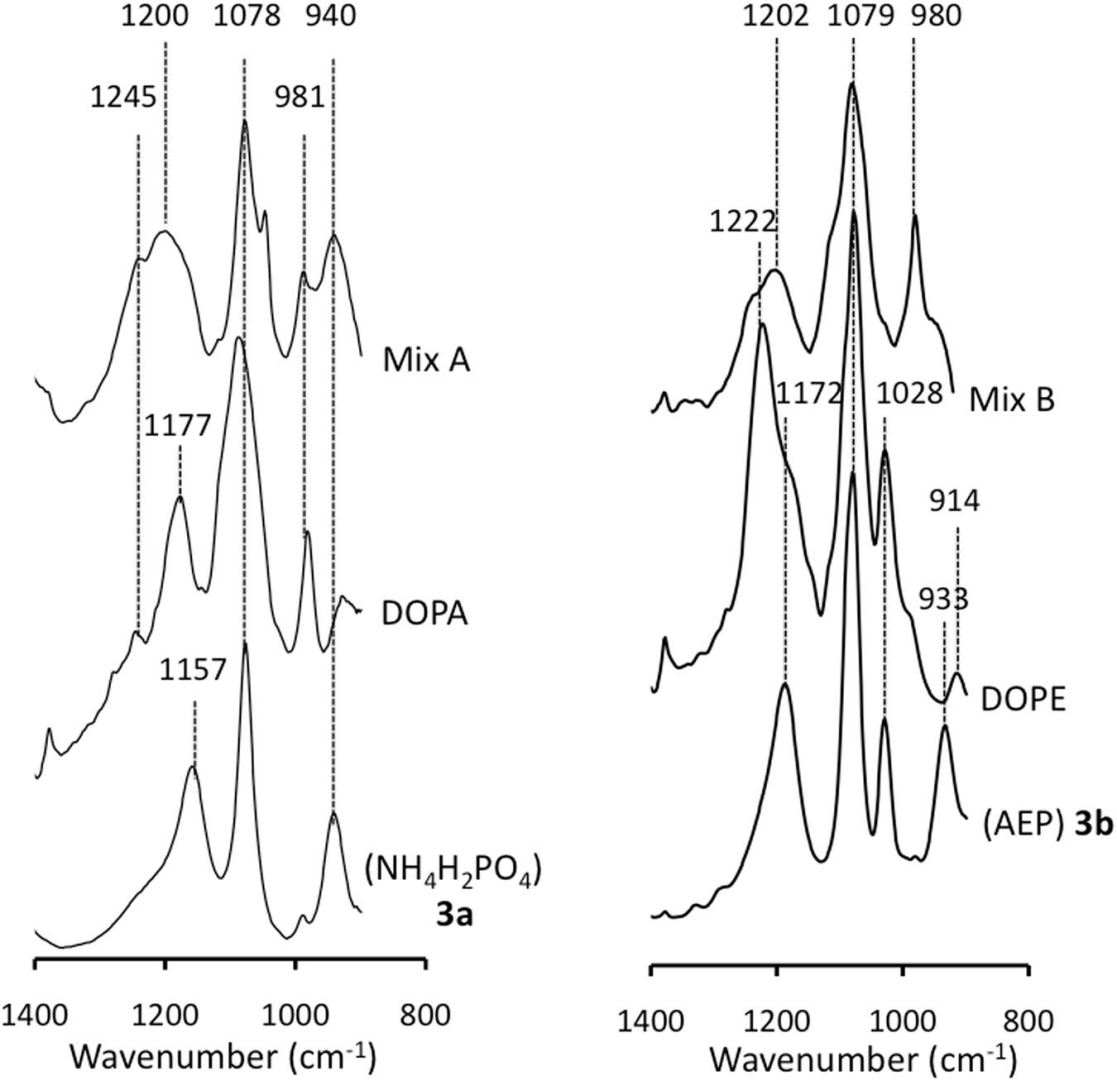
Left side: Infrared spectra of crude mixtures containing *rac*-DOPA (**4a**, top lane) obtained under prebiotic conditions compared to that of commercial DOPA (middle lane) and commercial NH_4_H_2_PO_4_ (**3a**, bottom lane). Right side: Infrared spectra of crude mixture containing *rac*-DOPE (**5**, top lane) obtained under prebiotic conditions compared to that of commercial DOPE (middle lane) and commercial 2-aminoethyl phosphate (**3b**, bottom lane). All samples were analyzed in 100 mM tris buffer pH = 7.8.

The infrared spectra of the racemic mixtures containing *rac*-DOPA and *rac*-DOPE (Fig. 3, top lanes) indicated at least three distinct chemical compounds. Several characteristic peaks on the infrared spectra of commercial DOPA and DOPE (Fig. 3, middle lanes) located at 1245, 1078 and 981 cm^−1^ for DOPA and 1222, 1078–1079 cm^–1^ for DOPE were found in the racemic mixtures containing *rac*-DOPA and *rac*-DOPE (Fig. 3, top lanes). In addition, the vibrations derived from the added phosphate sources (for P_i_ 940 cm^−1^ and for AEP 933 cm^−1^) in the crude prebiotic mixtures (Fig. 3, top lanes) indicated the residual phosphate vibrations of the phosphate sources (Fig. 3, bottom traces). Additional peaks such as the satellite peak at around 1078 cm^−1^ in crude mixture containing rac-DOPA, and at 980 cm^−1^ in mixtures containing *rac*-DOPE (Fig. 3, top lanes) suggested the presence of the third chemical compound for both *rac*-DOPA and *rac*-DOPE mixtures.

### Vesiculation of crude mixtures A and B

**Figure 4:**
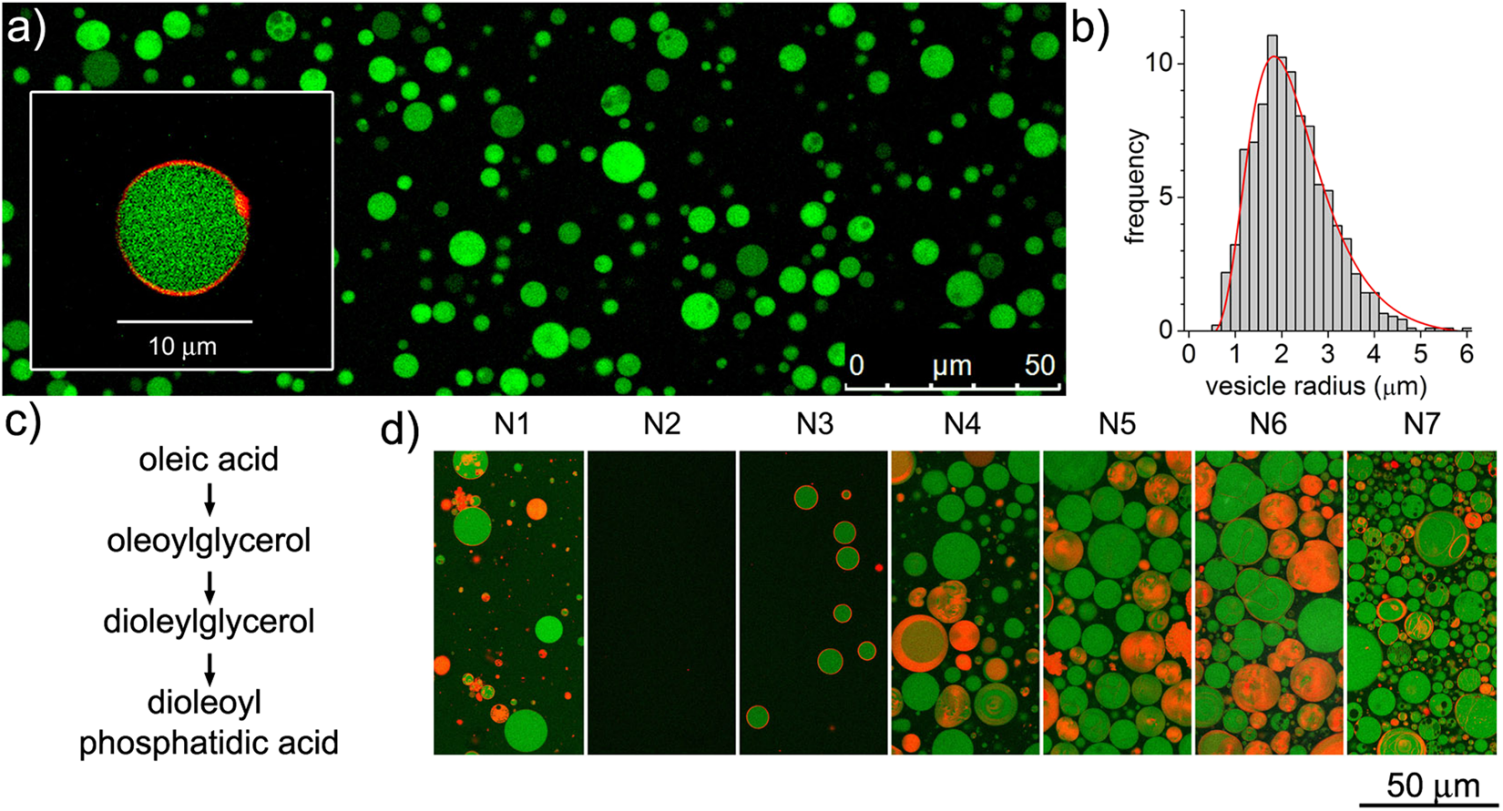
(**a**) GVs obtained from the gentle hydration of crude reaction mixture Mix A. GVs were further characterized by flow cytometry (see Fig. 3 and Table S2). (**b**) Size distribution analysis, as obtained by image analysis, of GVs prepared from the lipid mixture Mix A. The experimental distribution (grey bars) has been fitted with a log-normal curve (red line). The population is described by the following mean  radius ± standard deviation: 2.2 ± 0.8 (n = 1826). Confocal microscopy images of calcein-filled GVs were firstly thresholded in order to distinguish GVs from background (on the basis of calcein fluorescence). The resulting binary image was analyzed for object recognition by the proprietary *image J* algorithm applying a shape filter (0.8 < circularity < 1.0). As outcome, a set of Region Of Interest (ROI) was obtained and overlayed over the initial picture, from which the GVs radii were obtained after a proper pixel-to-micrometer calibration. (**c**) The possible chemical pathway that converts oleic acid to phosphatidic acid realizes a combinatorial chemical space made of the four indicated amphiphilic compounds. (**d**) Hydration of lipid mixtures N1 to N7 gave, depending on their composition (cf. Table 1), GVs (green with or without orange-red membrane) and non-vesicle aggregates, i.e. red/orange filled objects without aqueous (green) internal volumes. In both cases vesicles were prepared by the slow hydration of the lipid mixtures in the presence of green-fluorescent calcein as water-soluble probe and small amounts (0.01–0.2 mol%) of co-hydrating commercial (enantiopure) DOPE-Rh as orange-fluorescent lipid derivative for membrane staining.

**Figure 5:**
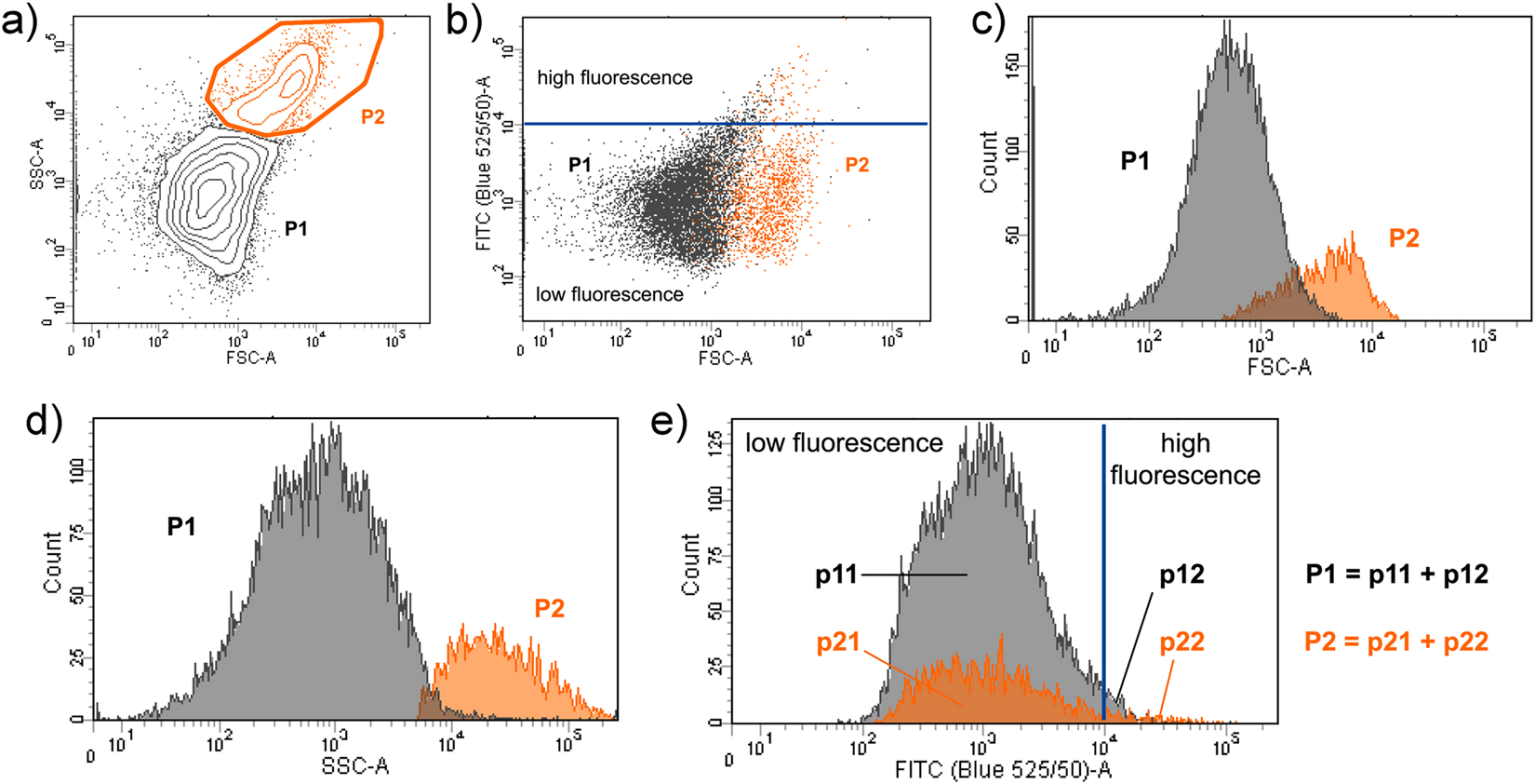
Flow cytometry analysis of vesicles obtained from the hydration of Mix A. **(a)** The whole vesicle population has been partitioned in two sub-populations (**P1** and **P2**) on the basis of the appearance of the side-scattering (SSC) *vs*. forward-scattering (FSC) contour plot; **(b)** green fluorescence (FITC) *vs*. FSC dotplot reveals two well distinguished populations, centered at fluorescence values of 10^3^ a.u. in the FITC channel, with a minor number of vesicles whose fluorescence is above an arbitrary threshold level of 10^4^ a.u.; (**c**) histogram of vesicle distribution over FSC values, revealing partially overlapping sub-populations; (**d**) histogram of vesicle distribution over the SSC values, revealing a good separation among the two sub-populations according to the SSC parameter; (**e**) histogram of vesicle distribution over the FITC values, where the value of 10^4^ a.u. has been used as threshold in order to distinguish normally-filled vesicles (green fluorescence <10^4^ a.u.), termed **p11** and **p21**, and highly-filled vesicles (green fluorescence >10^4^ a.u.), termed **p12** and **p22**. Note that **P1** = **p11** + **p12**, and **P2** = **p21** + **p22**. The quantitative analysis of vesicle populations is reported in Table S2.

The self-assembly of Mix A or Mix B was then tested by attempting the preparation of vesicles applying a natural ‘gentle’ hydration method^35^ under conditions that are widely used the formation of giant vesicles (GVs), *i.e*., micrometer sized spherical objects bearing an aqueous “lumen” inside volume being isolated from the external solution by at least one bilayer membrane composed of lamellar-phase lipids^36^. Figure 4a shows confocal fluorescence microscopy images of GVs obtained from Mix A. Stable GVs were obtained displaying the key property of encapsulating water-soluble compounds (calcein). Image analysis allowed to determine the GV’s size distribution (Fig. 4b) and the mean GVs  radius, which is 2.2 μm (standard deviation: ±0.8 μm). Flow cytometric analysis shows that the GV population of Mix A is composed of two sub-populations (ratio 4:1) distinguished by their light scattering (Fig. 5), where close to 3% of the GVs encapsulate green-fluorescent calcein with an exceptionally high efficiency (Table S2). A detailed microscopic analysis (Figs S39–S41, Table S3) shows that simple unilamellar vesicles coexist with more complex multi-vesicular structures. In contrast, GVs were barely detectable in the case of *rac*-DOPE containing Mix B.

### Vesiculation of model lipid mixtures

We then investigated the vesiculation of artificially reconstructed prebiotic mixtures (Table 1, lower part), which contain combinations of the four above-mentioned lipids to explore an expanded set of chemical compositions. The first set (M1–M4) revealed that mixtures containing DOPA rather than DOPE successfully produced GVs. In particular, 30 to 60 mol% DOPA, like DOPC (sample M5, see Figure S41 and Table S3), is a key ingredient for favoring bilayer assembly. Note that DOPC ideally derives from the phosphorylation of **1** with phosphocholine. In contrast, analogous amounts of DOPE (entries M2 and M4) did not promote the formation of vesicles.

Next, we explored in a more systematic way quaternary mixtures composed of **8**, **7a**, **1** and DOPA (N1–N7) simulating the stepwise “transition” from very primitive fatty acid vesicles to PA vesicles, noting that these four compounds might be the members of an hypothetical OA-to-DOPA primitive pathway (Fig. 3c). In other words, we sampled the compositional space that realistically emerges when primitive chemical interconversion routes are available for these four compounds. Confocal microscopy was used to image the corresponding GVs (Fig. 4d). While the binary 8:2 mixture of **8** and **7a** (entry N1) forms GVs as expected^7,37^, the inclusion of 20 mol% of **1** inhibits GV formation (entry N2). By adding 20 to 80 mol% DOPA (N3 to N6) GVs are again observed, although they coexist with non-vesicle aggregates, probably lipid “clumps” based on mixtures of lamellar and non-lamellar lipid phases (wholly orange-red objects in Fig. 4d).

A full account of these observations would require a detailed study on the whole-phase diagram of the lipids under investigation, as the prediction of the lipid(s) preferred phase (lamellar, hexagonal, cubic, etc.) is still conceptually challenging. The surfactant packing parameter *p* is often used to discuss lipid phases.

## Discussion

Initially introduced by Israelachvili and co-workers in 1976^38^, the dimensionless parameter *p* is defined as:

$$p=\frac{v}{{a}_{0}\cdot {l}_{c}}$$

where *v* is the molecular volume, *l*_*c*_ is the chain length and *a*_0_ is the headgroup surface of the surfactant molecule in the aggregate. The range of *p* values can be sectioned according to the type of aggregate. For example, small *p* values (<1/3) are typical of spherical micelles (normal micelles), large *p* values (>1) are instead typical of inverted phases, like reverse (or inverted) micelles or the inverted hexagonal phase (with a strong dependence on the exact conditions), when 1/3 < *p* < 1/2 elongated “tubular” micelles generally form, intermediate *p* values 0.5–1 are instead typical of the lamellar phase (*L*_*α*_) – and thus a curved bilayer as membranes in vesicles. Whereas it is allowed to safely infer the *p* value once the surfactant aggregation phase is known, the contrary is not true, i.e., a *p* value cannot be easily derived from the molecular shape. The reason is that the values *v*, *l*_*c*_ and especially *a*_0_ *in the aggregate* are difficult to predict. In particular *a*_0_ results from the balance between attractive and repulsive forces of the surfactant in the aggregate^39,40^. Salt type and content, pH, temperature, interaction with other lipids, etc. play a role in determining the surfactant aggregation phase. The value of *p*, in conclusion, is generally not an *a priori* parameter. In the case of surfactant mixtures, the prediction of the most stable phase is challenging, the multiple surfactant-surfactant interactions are hardly predictable as well as the role of the solvent. In other words, we are facing emergent properties.

### Discussion on the association phase formed by lipids

Despite these limitations, lipid phases have been – and are – qualitatively discussed in terms of their “molecular shape”^41^. It is then possible to cautiously discuss on the mixtures used in this work as it follows.

The aggregation properties of oleic acid have been well characterized with respect to its ability to form vesicles. It is well known that oleic acid can form vesicles or micelles depending on the pH^5–7,42,43^. At high pH (>9.5), the carboxylate headgroups experience a repulsive force due to the negative charge and are plausibly more hydrated, so that *a*_0_ is large and *p* is small. Oleate micelles are formed under these conditions. In contrast, at pH 8.5 one observes the formation of vesicles because *a*_0_ decreases and *p* increases. The decrease of *a*_0_ is due to attractive forces between only partially ionized carboxylate groups, i.e., near the p*K*_a_ of oleic acid in the bilayer. The observed p*K*_a_ of oleic acid in such a lamellar phase (*L*_α_) is about 4 pH units higher than in the hypothetical monomeric form (expected to be similar to short chain carboxylic acids, i.e., around 4.5), because the removal of protons from the oleic acid molecules, thus, the generation of negative charges of oleate assembled in the bilayer becomes difficult. Bridging hydrogen bonds are responsible for attractive forces between the carboxylate headgroups^44^. In addition to the head group protonation, hence the pH, the type and the amount of counter ions play a role that determine the stability of fatty acid bilayers. Indeed, fatty acid vesicles should be better recognized as mixed vesicles made of fatty acids and their soaps. Counter ions like Na^+^, K^+^ and others (e.g., organic ammonium, guanidinium)^45^ interact with the polar headgroups and fine-tune their local associative properties and the resulting type of aggregate. Moreover, when present in large excess (e.g., oleate vesicles in sea water), counter ions destabilize fatty acid vesicles^20,37^.

Minor amounts of MOG (**7a**) do not alter these OA features^16–18^. Saturated monoacylglycerols have been used in mixtures with the parent free acid to create vesicles that were more resistant to high concentrations of Mg^2+^ and other ions, when compared to vesicles made of the oleic acid only^8,16,37^. Although some saturated monoacyl-glycerols may form vesicles by themselves under certain conditions^12^, in the case of unsaturated monoacyl-glycerols the *cis* double bond increases the effective cross-sectional area and volume of the hydrophobic tail and the formation of reversed phases is favored. However, monooleoylglycerol (<10%)/oleic acid vesicles have been reported^46^, as well as mono-myristoleoylglycerol (<33%)/myristoleic acid vesicles^7^. The bilayer-forming tendency of the fatty acid overcomes the monoacylglycerol effects due to their small molar fraction.

Dioleoylglycerol (and in general, diacylglycerols) have been often investigated in mixtures with phospholipids. Diacylglycerols are hydrophobic molecules with little interaction with water. They have generally a pronounced conical shape (small *a*_0_, large *p*)^47,48^ and are strong perturbers of planar bilayers, affecting both chain packing and head group conformation^49^, leading to an inverted hexagonal (*H*_II_) phase or cubic phase. Diacylglycerols are known to induce the *L*_α_-*H*_II_ phase transition in PCs (phosphocholines)^50^. However, it seems that, in order to play this role, the PC acyl chains need to bear unsaturations^48^.

DOPA self-associates as a lamellar phase and forms vesicles^51,52^. DOPA bears a phosphomonoester headgroup and therefore is a diprotic acid of the form R-O-(P=O)-(OH)_2_ with two ionization constants p*K*_a1_ and p*K*_a2_. Their values have been measured by different techniques and result to be around 3.5 and 8, respectively^53^. Note that the first two p*K*_a_s of H_3_PO_4_ are around 2 and 7, so that also in this case a shift toward higher p*K*_a_ values is observed due to molecular association. Thus, around pH 7 DOPA is a largely monoanionic compound, with the possibility of establishing hydrogen bonds between headgroups^53–55^, as suggested in the case of oleic acid. At neutral pH and in the presence of Mg^2+^ or Ca^2+^, DOPA is a lamellar phase-forming lipid^52,56^ but due to the fact that its *a*_0_ actually depends on phosphate-proton and phosphate-ion interaction, the changes in temperature, pH and salt content strongly affects its behavior. For example, at pH 5, and in the presence of Ca^2+^ (over a certain molar ratio) the inverted hexagonal phase has been reported^56^.

Phosphatidylethanolamine (PE) is a class of zwitterionic lipids which is prone to form an inverted hexagonal phase^57^. DOPE is zwitterionic, like DOPC, but its headgroup is much smaller and carries a primary ammonium group at neutral pH that may become deprotonated at higher pH values, whereas the positive charge of the quaternary ammonium group of phosphocholines remains pH-independent. DOPE self-associates in an aqueous solution as a lamellar phase or as an inverted hexagonal phase, depending on the temperature (a transition has been reported to be at 3 °C, but also at around 10 °C) and water content^58^. Due to this feature, DOPE is the most common non-bilayer forming lipid used for the preparation of “fusogenic” drug delivery vesicles^59^ because, during membrane-membrane fusion, regions with negative curvature must be formed – and in these regions non-bilayer forming lipids find their “natural” place^60^. In other words, non-bilayer lipids promote membrane fusion. However, as the PE headgroup has mobile protons, pH plays a decisive role, thus, the *L*_*α*_-*H*_II_ phase transition strongly depends on pH (and it is occasionally abolished). Negatively charged PE forms vesicles at high pH (>9) in the absence of cations like Ca^2+^ ^61^.

Thus, pure DOPE vesicles have been prepared at pH 9.5, maintained their inner content (leak-free), but had a tendency to aggregate^62^. PCs in general, and POPC and DOPC in particular, are a well-known bilayer-forming phospholipids (Tresset, 2009 and references therein)^63^. PC-rich mixtures generally bear a good propensity to form vesicles with a diminished pH dependence.

### Discussion on the association phase formed by racemic lipid mixtures Mix A and Mix B

Based on the above consideration, it is possible to rationalize the observations as follows (*i-vi*), recalling, that—to simplify—OA, DOPA and DOPC are bilayer-forming lipids, whereas DOG and DOPE are non-bilayer-forming lipids and actually favor the *H*_II_ phase. MOG can be incorporated up to a certain amount in bilayers without a strong destabilization. The stereochemistry of natural lipids is briefly addressed under (*vii*).

(i) Pure oleic acid (OA) form vesicles at pH 8.5 (actually consisting in a ca. equimolar mixture of oleic acid and oleate).
(ii) By adding monooleoylglycerol (MOG) to oleate there could be a destabilization of the oleic acid/oleate bilayer, but this depends on the MOG molar fraction. It is interesting to note that our results show, unexpectedly, that when GVs are formed from pure oleic acid in 200 mM bicine (pH 8.5), without the initial addition of sodium oleate, the presence of MOG (20 mol%) actually improves the formation of mixed oleic acid/oleate/MOG GVs.
(iii) The ternary systems composed by OA, MOG, and dioleoylglycerol (DOG, 20 mol%) do not form vesicles because of the DOG presence, which possibly act in couple with MOG to abrogate the bilayer-forming capacity of OA at this pH.
(iv) The quaternary system of Mix A, that includes OA, MOG, DOG and DOPA, recovers the capability of self-assembly in form of bilayer due to the presence of two bilayer-forming compounds, OA and DOPA at pH 7.5. This pH value is not the best for OA which – in a pure OA system – should be 90% in the protonated form. However, due to the presence of both acidic and basic sites on the DOPA headgroup, OA could be ionized and form H-bonds and/or salt bridges with DOPA heads and, in reverse fashion, undissociated carboxylic acid heads could H-bond with anionic oxygen of DOPA heads.
(v) The quaternary system of Mix B that includes OA, MOG, DOG and DOPE, the presence of non-bilayer-forming lipids overwhelms the OA capacity of holding a lamellar phase, and no vesicles can be formed.
(vi) The quaternary system M5 that includes OA, MOG, DOG and DOPC, as it happens for Mix A, has an overall good propensity for the lamellar phase, as OA and DOPC tendencies are the driving forces for getting a bilayered association phase (see Figure S41 and Table S3).
(vii) Phospholipids such as phosphatidic acids (PA), phosphatidylethanolamines (PE) and phosphatidylcholines (PC) possess a stereogenic centre at the C-2 carbon atom of the glycerol backbone. The natural enantiomer for all diacylglycerol phospholipids is d (Fischer convention) or 2 *R* ^64^ — the opposite is true for the natural phospholipidic isoprenoid glycerol ethers of *Archea* membranes^65^. Notably, together with the problem of the emergence of homochirality, another problem fascinated scientists, v*iz*. the fact that *Archaea*, the third domain of life, have a distinct molecular nature (phospholipids ethers) as compared with those (phospholipid esters) found in *Bacteria* and *Eukarya* ^65^. The problem of the divergent evolution of phospholipids in the three domains of life was studied by Shimada & Yamagishi (2011)^66^, Koga^67^ and more recently by Goñi and Mirazo (2014)^68^. However, for this research we only consider the formation of acyl glycerols as starting material for the synthesis of racemic phospholipids.

Few works were carried out in the field of homo- and hetero-chirality of phospholipids. Among others, Weis and McConnell reported in 1984 on the effect of chirality on the organization of lipid membranes and reported that enantiopure and racemic mixtures of dipalmitoyl phosphocholine (DPPC) possess different properties in the solid state^64^. Ock-Youm and Carreira reported that membranes composed of one of the two possible enantiomers can influence the activity of (enantiopure) biologically active molecules^69^. The pioneering simulated prebiotic syntheses of phosphatidic acids (PA), phosphatidyl ethanolamines (PE) and phosphatidylcholines (PC) reported by Deamer & coworkers and Oró & coworkers^10,13,14,25,26,30,31^ started from achiral glycerol, *n*-alkylic aldehydes or *n*-alkanoic acids in the presence of achiral condensing agents, thus, necessarily yielding the described products as racemic mixtures—accordingly, no optical rotations were reported (see section III in Electronic Supplementary Information). Since vesiculation from racemic lipids is readily achieved, this argues for the notion that natural “symmetry breaking”, i.e. the spontaneous generation of an imbalance between left- and right-handed enantiomeric natural molecules (chiral carbohydrates, amino acids and phospholipids), has most likely occurred in the aftermath of the formation of primitive membranes that formed vesicles, thus, that were already able to encapsulate newly formed biopolymers.

## Concluding Remarks

We draw three major conclusions from this study. First, starting from a pure pre-phospholipid intermediate, *rac*-DOG (**1**), realistic prebiotic conditions bring about a complexification of the system generating by-products that have similar self-assembling properties to those of the chemically expected end-products, leading to lipid mixtures with unique and difficult-to-predict physico-chemical and supramolecular properties. The chemical conditions carried out to promote phosphorylation of *rac*-DOG (**1**) in an open Eppendorf vial had a double-role of mimicking hydrothermal pools at close to neutral pH undergoing quite rapid evaporation of water^70,71^, as well as—under neat conditions—dry sea beds where super-concentrated amphiphile mixtures are generated upon (periodic) rehydration. Selection rules thus apply to these systems based not only on the availability of chemical interconversion pathways^72^ but also the aggregation properties of the resulting mixtures. Second, **1** cannot be an end-point product of primitive lipid synthesis because its accumulation would cause the collapse of lipid membranes. Its transformation to the phosphorylated form (*i.e*., PA or PC, but not PE) was the way to avoid such a scenario. We conclude, therefore, that avoiding diayclglycerol accumulation was one of the main driving forces for the transition of fatty acids to phospholipids in general, and PAs in particular. Third, prebiotic racemic mixtures of phospholipids and chemically related amphiphiles were able to form stable GVs, which supports the notion^66^ that the prebiotic amphiphile pool need not have been enantioenriched for the compartmentation of foreign hydrosoluble molecules. This study calls for further and more detailed theoretical and experimental investigations in the arena of primitive lipid mixtures, also considering the origin of homochiral membranes and various phosphoric polar head groups. All this will shed light on the nature of early micro-compartments and justify the appearance of protocells under plausible geochemical conditions.

## Electronic supplementary material

Supporting Information
