# Supplementary material for: Crude phosphorylation mixtures containing racemic lipid amphiphiles self-assemble to give stable primitive compartments
Source: Sci Rep. 2017 Dec 22;7:18106. doi: 10.1038/s41598-017-18053-y (PMC5741756; doi:10.1038/s41598-017-18053-y)
Supplement: Supplementary file 1 — Supporting Information [file 41598_2017_18053_MOESM1_ESM.pdf]

Supporting Information for

Crude phosphorylation mixtures containing racemic lipid amphiphiles self-assemble to give stable primitive compartments

Dimitri Fayolle,[†] Emiliano Altamura,[‡] Alice D'Onofrio,[†] Warren Madanamothoo,[†] Bernard Fenet,[†] Fabio Mavelli,[‡] René Buchet,[†] Pasquale Stano,* [§] Michele Fiore* [†] and Peter Strazewski* [†]

[†]Institut de Chimie et Biochimie Moléculaires et Supramoléculaires, Université de Lyon, Claude Bernard Lyon 1, 43 bvd du 11 Novembre 1918, F–69622 Villeurbanne Cedex, France

[‡]Department of Chemistry, University of Bari, Via E. Orabona 4, I–70125 Bari, Italy

[§]Biological and Environmental Science and Technology Department, University of Salento, Ecotekne, I–73100 Lecce, Italy;

Corresponding authors:

Michele Fiore: michele.fiore@univ-lyon1.fr

Peter Strazewski: strazewski@univ-lyon1.fr

Pasquale Stano: pasquale.stano@unisalento.it

Abstract. It is an open question how the chemical structure of prebiotic vesicle-forming amphiphiles complexified to produce robust primitive compartments that could safely host foreign molecules. Previous work suggests that comparingly labile vesicles composed of plausibly prebiotic fatty acids were eventually chemically transformed with glycerol and a suitable phosphate source into phospholipids that would form robust vesicles. Here we show that phosphatidic acid (PA) and phosphatidylethanolamine (PE) lipids can be obtained from racemic dioleoyl glycerol under plausibly prebiotic phosphorylation conditions. Upon *in situ* hydration of the crude phosphorylation mixtures only those that contained *rac*-DOPA (not *rac*-DOPE) generated stable giant vesicles that were capable of encapsulating water-soluble probes, as evidenced by confocal microscopy and flow cytometry. Chemical reaction side-products (identitied by IR and MS and quantified by ^{1}H NMR) acted as co-surfactants and facilitated vesicle formation. To mimic the compositional variation of such primitive lipid mixtures, self-assembly of a combinatorial set of the above amphiphiles was tested, revealing that too high dioleoyl glycerol contents inhibited vesicle formation. We conclude that a decisive driving force for the gradual transition from unstable fatty acid vesicles to robust diacylglyceryl phosphate vesicles, was to avoid the accumulation of unphosphorylated diacylglycerols in primitive vesicle membranes.

Table of contents

I Materials and Methods 3

II Experimental Procedures 4

II.a General procedure for the simulated prebiotic formation of amphiphiles 4

II.b General procedure for control experiments 4

II.c General procedure for the extraction of amphiphiles from crude prebiotic mixtures 4

III Results and Supplementary Discussion 5

III.a Specific rotation of prebiotic mixtures containing *rac*-DOPA (Mix A), *rac*-DOPE (Mix B), pure DOPA and pure DOPE 5

III.b ^{1}H NMR analysis of prebiotic mixtures containing *rac*-DOPA (Mix A) and *rac*- DOPE (Mix B) 5

III.c ESI-MS analysis of crude prebiotic reaction mixtures 9

IV NMR spectroscopic characterization of commercial compounds 15

IV.a 1,2–Dioleoyl–*sn*–glycero–3–phosphate (commercial DOPA) 15

IV.b 1,2–Dioleoyl–*sn*–glycero–3–phosphoethanolamine (commercial DOPE) 15

IV.c (9*Z*)–octadec-9-enoic acid (commercial OA) 15

V Synthesis of *rac*–DOG (1) and *rac*–MOG (7a) 17

V.a 3–*O*–Triphenylmethyl–DL–glycerol (9) 17

V.b 1,2-*O,O*-Dioleoyl-DL-glyceryl-3-*O*-triphenylmethyl ether (10) 18

V.c 1,2-*O,O*-Dioleoyl-DL-glycerol (1) 18

V.d *(4R/S)*-(2,2-Dimethyl-1,3-dioxolan-4-yl)methyl oleate (11) 18

V.e *(2R/S)*-2,3-dihydroxypropyl-1-oleate (7a) 19

VI Quantitative analysis of Mix A vesicles as derived from flow cytometry 29

VII Additional confocal fluorescence micrographs of Mix A, M1–4 and M5 30

I Materials and Methods

Oleic acid was purchased from Sigma-Aldrich and TCI-Europe and used without further purification. 1,2–dioleoyl–*sn*glycero–3–phosphate (DOPA), 1,2–dioleoyl–*sn*–glycero–3–phosphoethanolamine (DOPE), 1,2–dioleoyl–*sn*–glycero–3–phosphocholine (DOPC) and 1,2-dioleoyl-sn-glycero-3-phosphatidylethanolamine-*N*-lissamine rhodamine B sulfonate (DOPE-Rh) were purchased from Avanti Polar Lipids, Alabaster AL (USA). All the other reagents and solvents were purchased from Sigma-Aldrich and TCI Europe and were used without further purification. HPLC solvents were purchased from Thermo-Fisher Scientific (mass spectrometry grade).

Thin-layer chromatography (TLC) was carried out on aluminium sheets coated with silica gel 60 F254 (Merck). TLC plates were inspected by UV light (λ = 254 nm) and developed by treatment with a mixture of 10% H_2SO_4 in EtOH/H_2O (1:1 *v/v*), $KMnO_4$ 10% solution or the *Pancaldi* reagent ($(NH_4)_6MoO_4$, $Ce(SO_4)_2$, H_2SO_4, H_2O) followed by heating.

IR data were acquired with a Thermo Scientific Nicolet iS10 spectrometer equipped with a DTGS detector. The IR spectra were recorded with 64 interferograms at 4 cm^{-1} resolution each and then Fourier transformed.

Optical rotations were measured as $CHCl_3$ solutions on a JASCO P-1010 digital polarimeter and converted to specific rotations $[\alpha]_D$.

ESI–MS and UPLC–HRMS analyses were performed on a Bruker Impact II equipped with a hybrid mass spectrometer quadrupole type.

NMR spectra were recorded in $CDCl_3$ on a Bruker Avance 300MHz spectrometer at 300 for ^1H nuclei and 75 for ^{13}C nuclei and on a Bruker Avance 500 MHz for ^1H nuclei. Chemical shifts of solvents ($CDCl_3$: δ_H=7.26 and δ_C=77.23) served as internal references. Signal shapes and multiplicities are abbreviated as br (broad), s (singlet), d (doublet), t (triplet), q (quartet), quint (quintet) and m (multiplet). Where possible, a scalar coupling constant *J* is given in Hertz (Hz).

I.a Giant vesicle (GV) preparation by the natural swelling method

Lipids (mixture of commercial lipids or crude extracts from reaction mixtures, with or without the addition of 0.01–0.2 mol% DOPE-Rh) were dissolved in dichloromethane (typically, 2 mL) and poured in a 10 mL cylindrical thick-wall round-bottom glass tube. The solvent was completely evaporated under reduced pressure using a rotatory evaporator. The resulting thin lipidic film was further dried for 30 minutes at 1 mbar/25 °C, and next hydrated for 16 hours – without shaking – with the aqueous buffer, termed "I-solution" (composed of 200 mM sucrose in 5 mM or 200 mM of Na-bicine, pH 8.5 or 200 mM sucrose in 25 mM Tris-HCl, pH 7.5), in order to obtain an overall 1-2 mM lipid concentration. The hydration temperature was 25 °C. When needed, 1-10 μM calcein was included in the I-solution. Three volumes of the thus obtained GVs were diluted with one volume of an aqueous isotonic buffer solution termed "O-solution" (composed of 200 mM glucose in 5 mM or 200 mM of Na-bicine, pH 8.5 or 200 mM glucose in 25 mM Tris-HCl, pH 7.5), and centrifuged at 5,000 rpm for 10 minutes in a bench-top Eppendorf mini-centrifuge. GVs were pelletted down in the Eppendorf tube due to the density difference between the I-solution and the O-solution. The supernatant was carefully removed and the pellet – which appears pink-red when DOPE-Rh is present – was re-suspended in 100 μL of fresh O-solution.

Note that Mix A and Mix B were hydrated by using one of the above mentioned I-solutions (pH 7.5 or pH 8.5) depending on the lipid composition. Exploratory experiments showed that a lower pH value (7.5) is advantageous for the hydration of PA-containing lipid mixtures. Consequently, reconstituted lipid mixtures (main text, Table 1) were treated with the I-solution based on 25 mM Tris-HCl, 200 mM sucrose (pH 7.5). These observations fit with previously published reports on conventional PA-based vesicles (Hauser and Gains, 1982; Hauser *et al*., 1983).

I.b GV imaging by confocal laser scanning fluorescence microscopy and image analysis

GV samples, being further diluted with O-solution when needed, were placed in a micro-well plate (#81821, ibidi GmbH, Martinsried, Germany). Each circular micro-well has a diameter of 5 mm and can hold a maximum of about 35 µL. Owing to the density difference between the I-solution and the O-solution the GVs accumulate after 10-20 minutes at the bottom of the micro-wells. Then GVs can be imaged by an inverted TCS SP5 confocal microscope (Leica Microsystems, Wetzlar, Germany). Samples

without DOPE-Rh could be membrane-stained directly in the micro-well by direct addition of Trypan Blue (final concentration 0.06% *w/v*). Fluorescence was measured by employing standard settings for sequential acquisition of green and orange/red fluorescence; objectives 40× and 63×. Digital image processing and analysis was carried out using ImageJ public domain software (Rasband, W.S., ImageJ, U.S. National Institutes of Health, Bethesda, Maryland, USA, https://imagej.nih.gov/ij , 1997-2016)

I.c Flow cytometry

GV samples were analysed using a BD LSRFortessa X-20 (Becton, Dickinson and Company, Franklin Lakes, NJ, USA) cell analyser. Forward scatter, side scatter and green fluorescence data were collected using a 488 nm laser with a power of 50 mW as excitation light source. For the forward scatter and the side scatter a photodiode detector with a 488/10 bandpass filter and a photomultiplier tube with a 488/10 bandpass filter have been used, respectively. The quartz cuvette flow cell is gel-coupled by refractive index-matching optical gel to the fluorescence objective lens (1.2 NA) for optimal collection efficiency. Emitted light from the gel-coupled cuvette was delivered by fiber optics to the detector arrays. The flow rate was 12 µL/min. The morphologic plot was side scatter *vs.* forward scatter and green emission was recorded in the wavelength range 500-550 nm. The number of GVs analysed in each run was about 10,000.

II Experimental Procedures

II.a General procedure for the simulated prebiotic formation of amphiphiles

In a typical experiment an Eppendorf tube (volume 2 ml), was filled with 0.025 mmol of *rac*-DOG (**1**, 15.2 mg) and mixed with 10 equivalents (0.25 mmol) of cyanamide (**2a,** 52.5 mg) or urea (**2b**, 75.1 mg) in the presence of 10 equivalents (0.25 mmol) of ammonium dihydrogen phosphate (**3a,** 28.7 mg) or 2-aminoethyl phosphate (AEP, **3b**, 35.2 mg). The heterogeneous mixture was suspended in 0.9 mL of ultrapure water and 0.1 mL of ethanol were added in order to dissolve **1** in water and avoid the formation of giant oil droplets. The obtained suspension was vortexed (1 minute), sonicated (5 minutes) and the obtained clear solution was left heated without any cap between 24 and 48 hrs in a thermo-shaker apparatus set to 80°C/500 rpm until dryness of the former water/ethanol mixture. Reaction mixtures were monitored by analytical TLC using eluent A ($CHCl_3$ 100%) and eluent B ($CHCl_3$:MeOH:H_2O, 65/25/4 *v*/*v*/*v*). A variant of this procedure was to mix all the reactants and to let them melt at 80°C without adding any solvent (neat conditions). All reactions were made in triplicate. For both reaction conditions, a solid yellowish residue was obtained.

II.b General procedure for control experiments

In a typical experiment *rac*-DOG (**1**) was mixed with cyanamide (**2a**) or urea (**2b**) without phosphate sources **3a** or **3b**. Similar reactions were performed in the absence of **2a** or **2b** but in the presence of **3a** or **3b**. Molar ratios, reaction times and conditions were the same for all control experiments. All control reactions were made in triplicate and in both reaction conditions used in the general procedures.

II.c General procedure for the extraction of amphiphiles from crude prebiotic mixtures

The crude mixtures were carefully dissolved in a minimum amount of water (1–3 mL) and transferred in a small separation funnel (10 mL) where the mixture was extracted three times with $CHCl_3$ or CH_2Cl_2 (3x5 mL). The combined organic layers were collected and evaporated in a small round bottom flask. Aliquots of those residues were submitted to NMR spectroscopic and vesiculation studies. TLC (eluent systems A or B) of the water phases showed no presence of amphiphiles and or lipophilic compounds.

III Results and Supplementary Discussion

III.a Specific rotation of prebiotic mixtures containing *rac*-DOPA (Mix A), *rac*-DOPE (Mix B), pure DOPA and pure DOPE

Crude mixtures "Mix A" and "Mix B", 10 mg each, were dissolved in chloroform (HPLC grade, 10 mL). Optical rotations were measured in triplicate and resulted in specific rotations $[\alpha]_D^{20}$ = 0.00 (*c* 0.1, $CHCl_3$) for both mixtures. The specific rotations of pure commercial DOPA and DOPE under the same conditions were $[\alpha]_D^{20}$ = +6.74 (*c* 0.1, $CHCl_3$) and, respectively, $[\alpha]_D^{20}$ = +5.29 (*c* 0.1, $CHCl_3$)

III.b ^{1}H NMR analysis of prebiotic mixtures containing *rac*-DOPA (Mix A) and *rac*-DOPE (Mix B)

Table S1. Composition of crude prebiotic mixtures containing racemic DOPA (**4**) and DOPE (**5**) together with starting material *rac*-DOG (**1**), by-products *rac*-MOPA (**4b**), *rac*-MOG (**7a**), *sec*-MOG (**7b**) and oleic acid (**8**) as determined by ^{1}H NMR spectroscopic analysis.

Entry[a]	Molecules	δ_H (ppm) [a]	Relative signal intensity [b]	mol %[c]
**Mix A** (end product: *rac*-DOPA)	**8** (oleic acid)	–	–	32.6
	**7a** (*rac*-MOG)	3.96	0.45	14.3
	**7b** (*sec*-MOG)	4.85	0.10	3.2
	**1** (*rac*-DOG)	5.01	0.26	8.3
	**4a** (*rac*-DOPA)	5.15	1.00	37.4
	**4b** (*rac*-MOPA)	5.17	0.10	4.2
**Mix B** (end product: *rac*-DOPE)	**8** (oleic acid)	–	–	21.9
	**7a** (*rac*-MOG)	3.96	0.63	20.0
	**7b** (*sec*-MOG)	4.85	0.05	1.6
	**1** (*rac*-DOG)	5.01	0.77	24.6
	**5** (*rac*-DOPE)	5.20	1.00	31.9

[a] of the central glycerol CH proton signal recorded at 500MHz; [b] from integration; [c] molar percentage in the mixture.

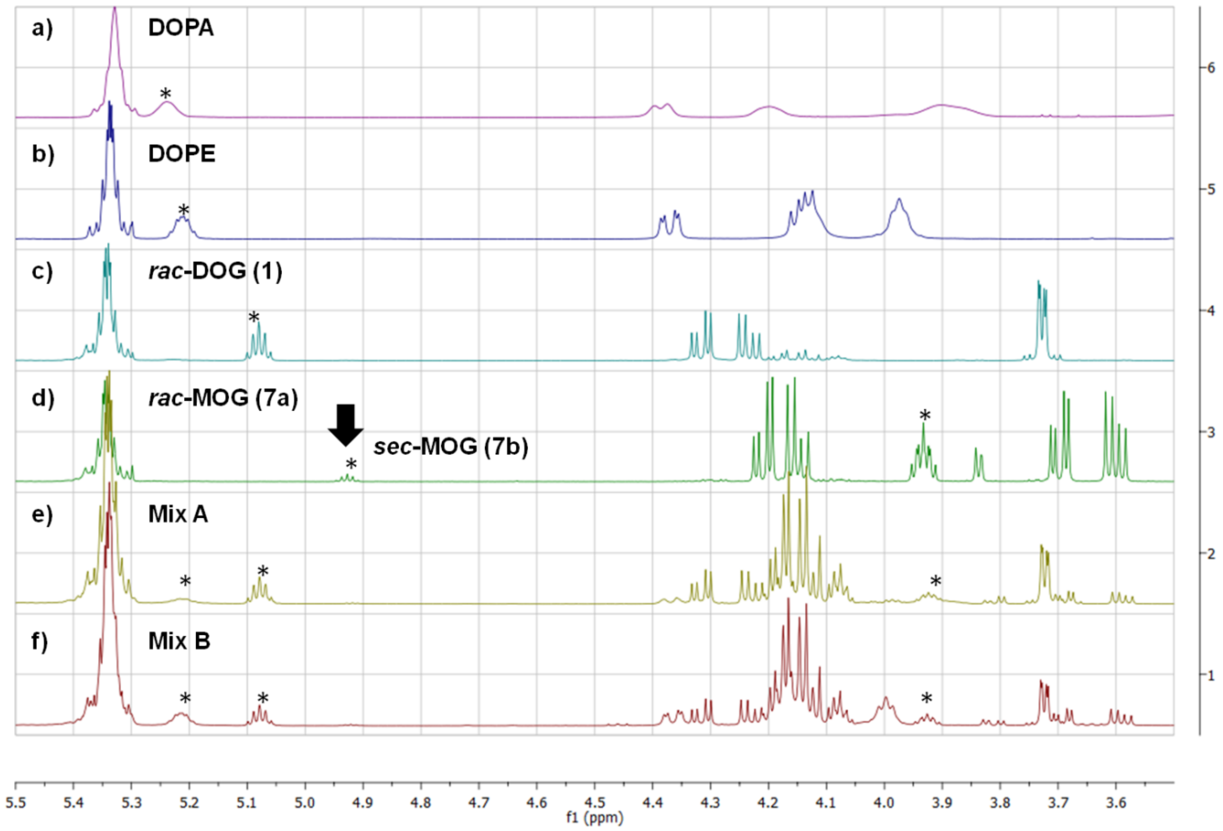

Figure S1. ^{1}H NMR recorded at 500 MHz in $CDCl_3$; chemical shift region for protons bound to carbon atoms in α-position to an oxygen atom or part of a –C=C– double bond, i.e. glycerol –CH– and –CH_2– groups and Z–CH=CH– of the oleoyl chains. **a**) Commercial DOPA; **b**) Commercial DOPE; **c**) *rac*-DOG (**1**); **d**) *rac*-MOG (**7a**) that contains traces of *sec*-MOG (**7b**) indicated by a black arrow; **e**) Mix A (containing **1**, **4a**, **4b**, **7a**, **7b** and **8**); **f**) Mix B (containing **1**, **5**, **7a**, **7b** and **8**); Asterisks indicate the secondary hydrogen atom of the glycerol backbone. Small amounts of adduct **6** were identified only by ESI-MS and formed only when **2a** was used as an activator. Samples of commercial and synthetic compounds were prepared as 25 mg of pure compound dissolved in 660 µL of $CDCl_3$.

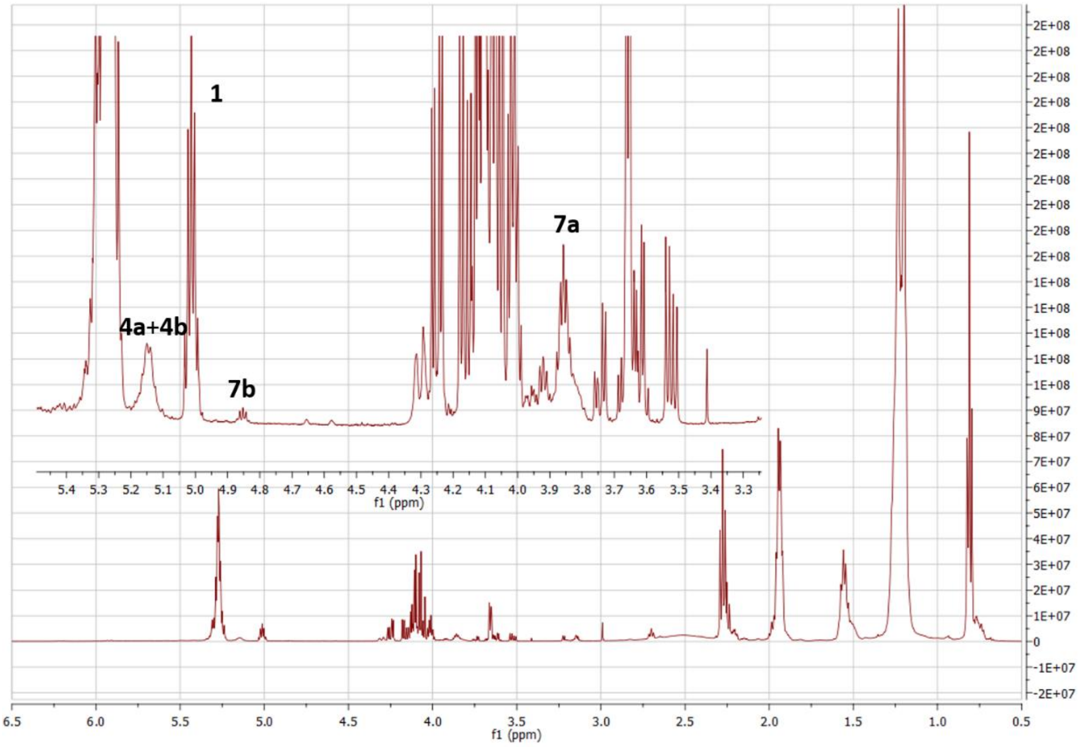

Figure S2. ^{1}H NMR (500MHz, $CDCl_3$) of crude extract of Mix A containing **1**, **4a, 4b**, **6**, **7a, 7b** and **8**. Molecules were identified (except **4b**, **6** and **7b**) by comparison with commercial or synthetic versions of the molecules.

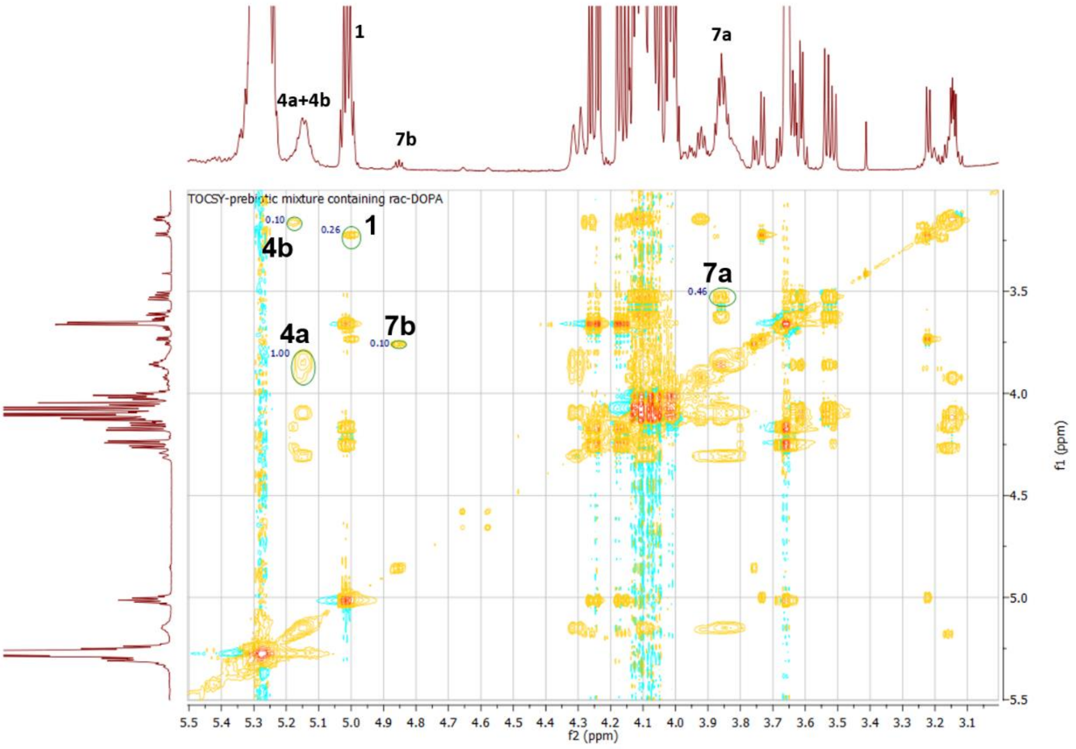

Figure S3. TOCSY NMR (500MHz, $CDCl_3$, region 5.5-3.0 ppm) of crude extract of Mix A containing **1**, **4a**, **4b 7a, 7b** and **8**. The green circles represent the 2D integration of the central glycerol backbone CH proton signal. In 1H NMR (Figure S3, the signals of **4a** and **4b** overlaps)

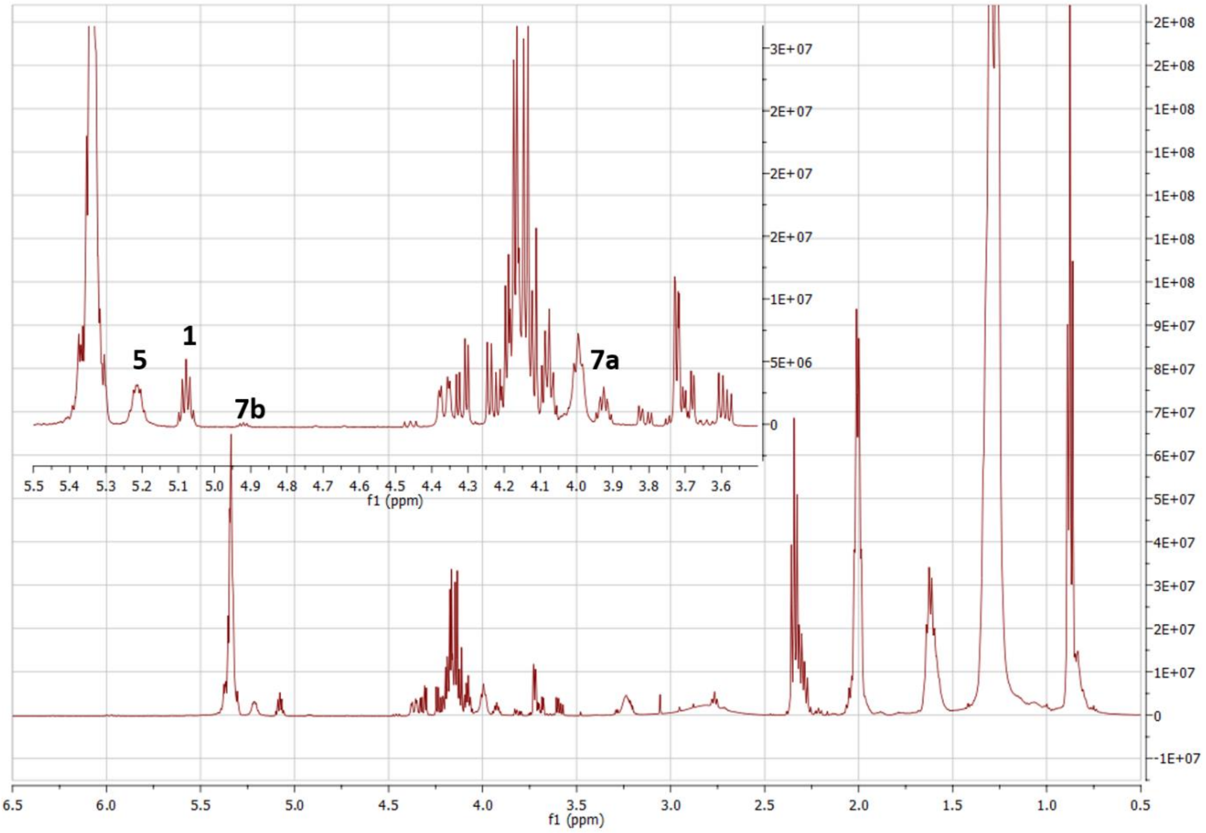

Figure S4. ^{1}H NMR (500MHz, $CDCl_3$) of crude extract of Mix B containing **1**, **5**, **7a**–**b** and **8**.

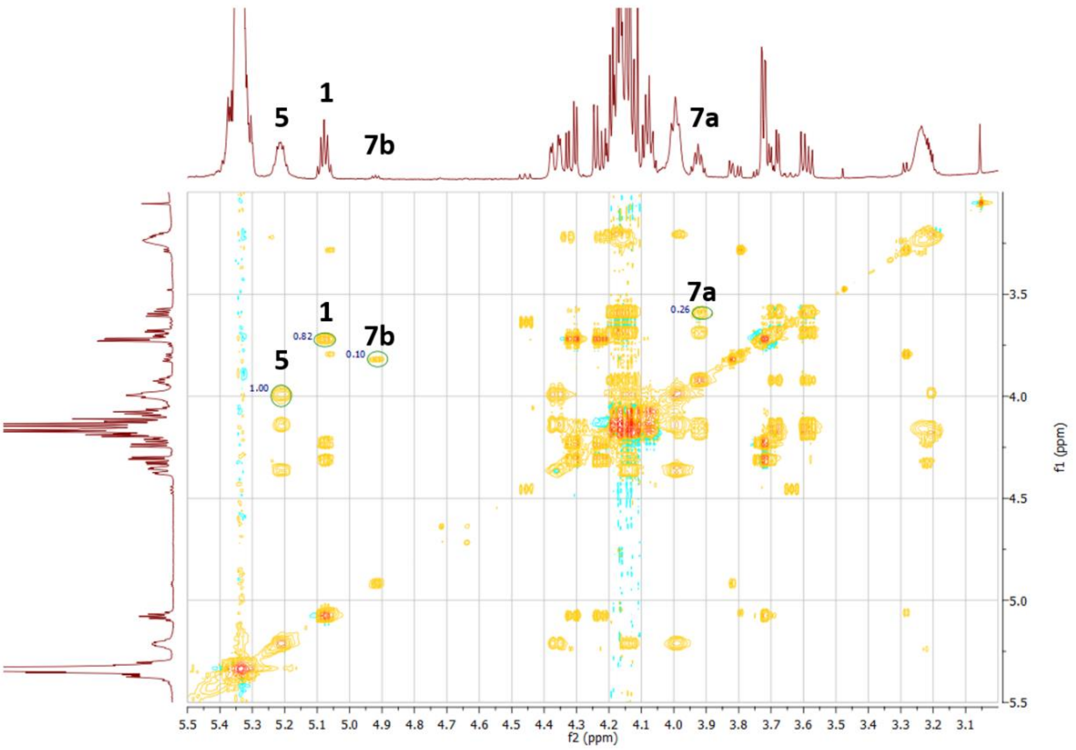

Figure S6. TOCSY NMR (500MHz, $CDCl_3$, region 5.5-3.0 ppm) of crude extract of Mix B containing **1**, **5**, **7a**–**b** and **8**.

III.c ESI-MS analysis of crude prebiotic reaction mixtures

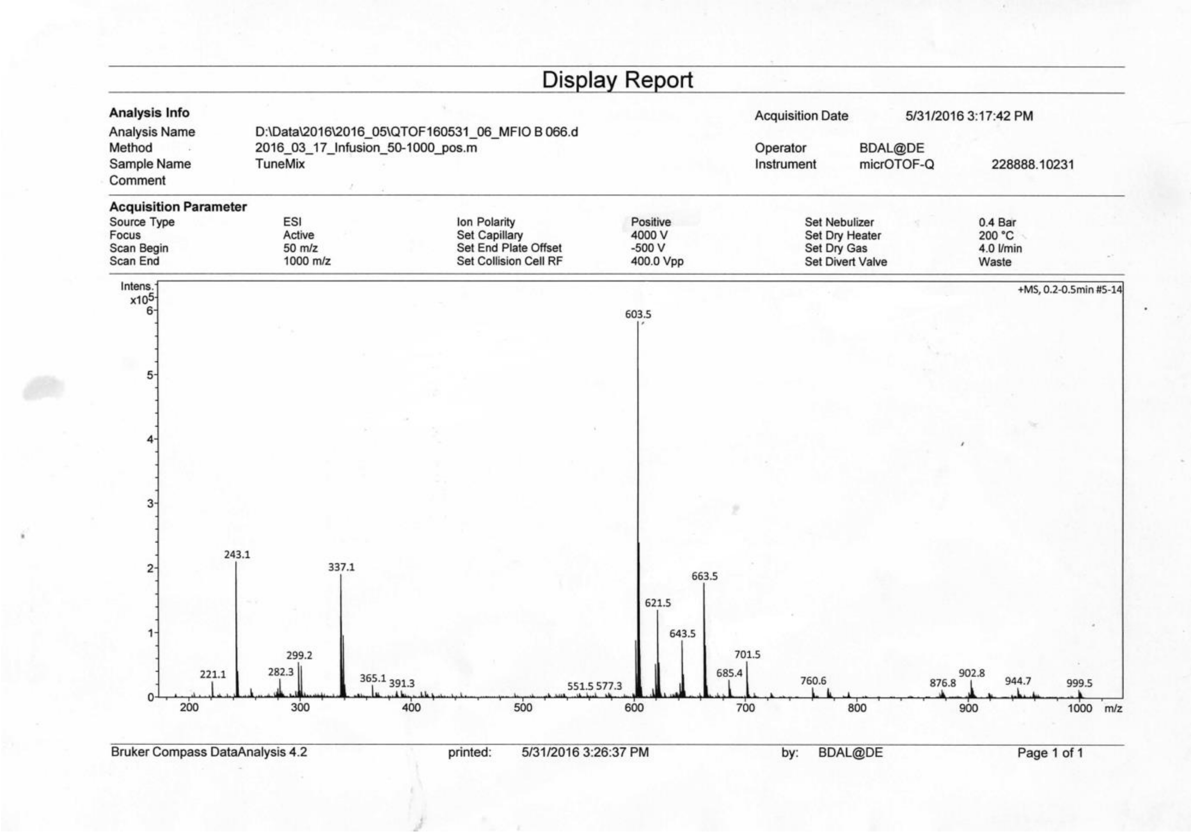

Figure S7. ESI-MS (positive ion mode) of crude reaction mixture obtained by heating **1** + **2a** + **3a** at 80°C for 48h. The use of urea (**2b**) gave similar results except the formation of **6** (m/z 663 $[M+H]^+$. Neat conditions gave similar results with **2a** or **2b.**

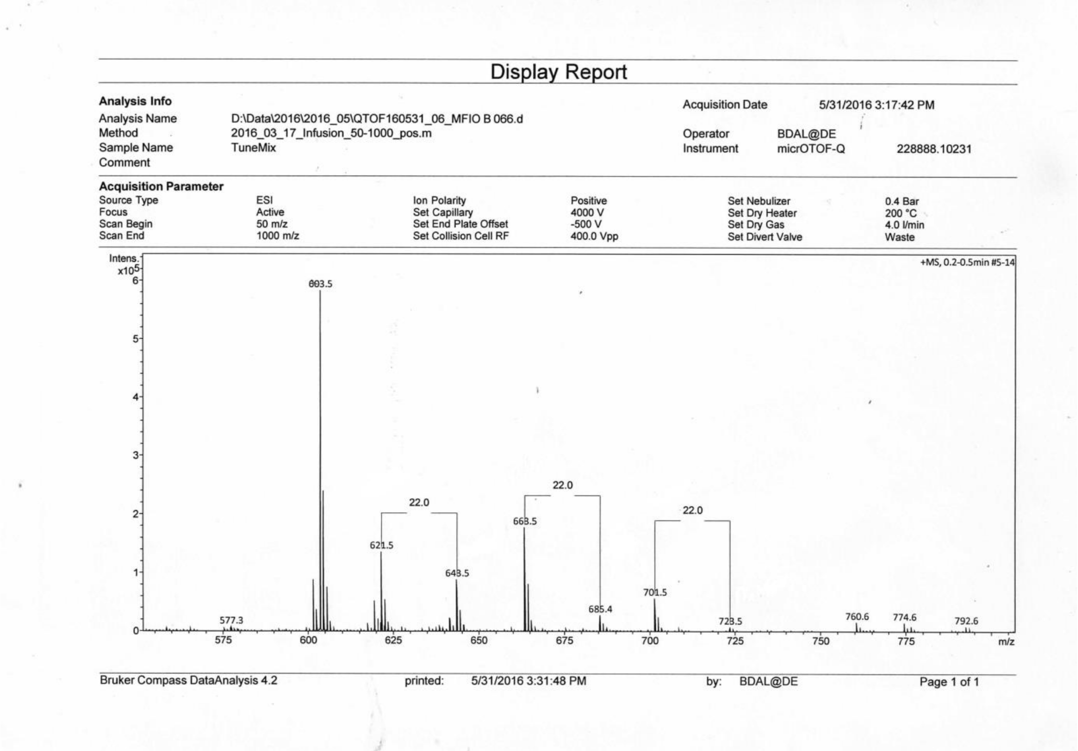

Figure S8. ESI-MS (positive ion mode) of crude reaction mixture obtained by heating **1** + **2a** + **3a** at 80°C for 48h. Fragmentation of the residual starting material as *m/z* 621 $[M+H]^+$, *m/z* 643 $[M+Na]^+$, *m/z* 603 $[M-H_2O]^+$ and fragmentation of the formed *rac*-DOPA (**4a**) as *m/z* 723 $[M+Na]^+$. Neat conditions gave same results with **2a** or **2b.**

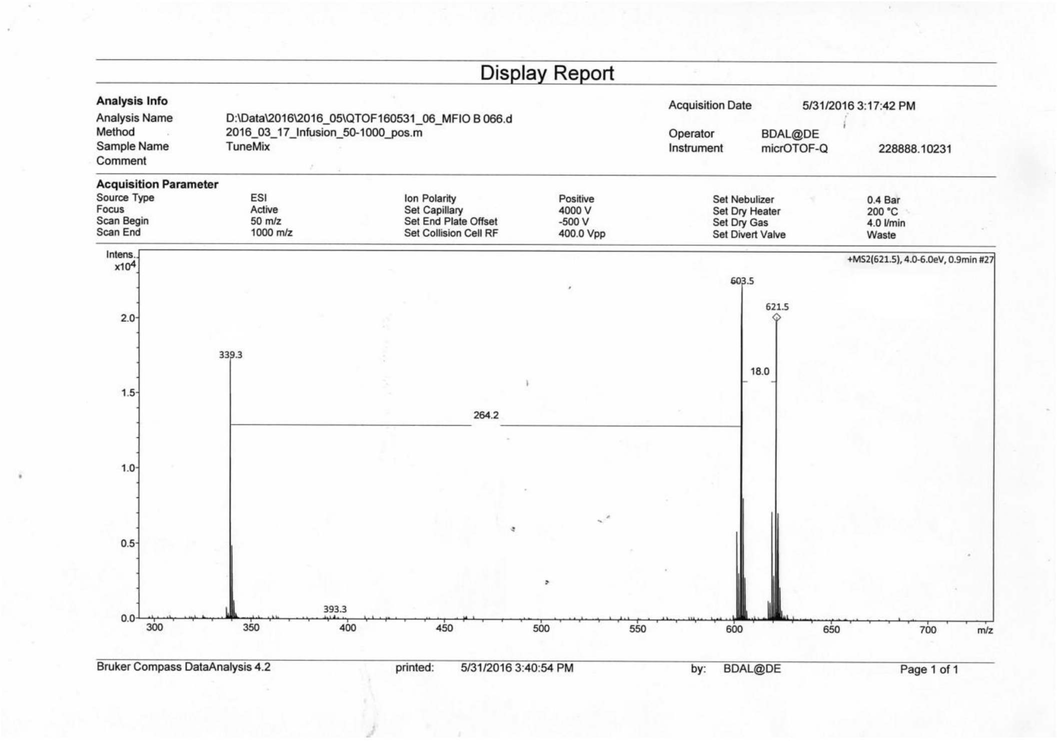

Figure S9. ESI-MS (positive ion mode) of crude reaction mixture obtained by heating **1** + **2a** + **3a** at 80°C for 48h. Fragmentation of the residual starting material **1** *m/z* 621 $[M+H]^+$ into *m/z* 603 (–H_2O) and 339.3 (–oleate+OH). Neat conditions gave same results with **2a** or **2b.**

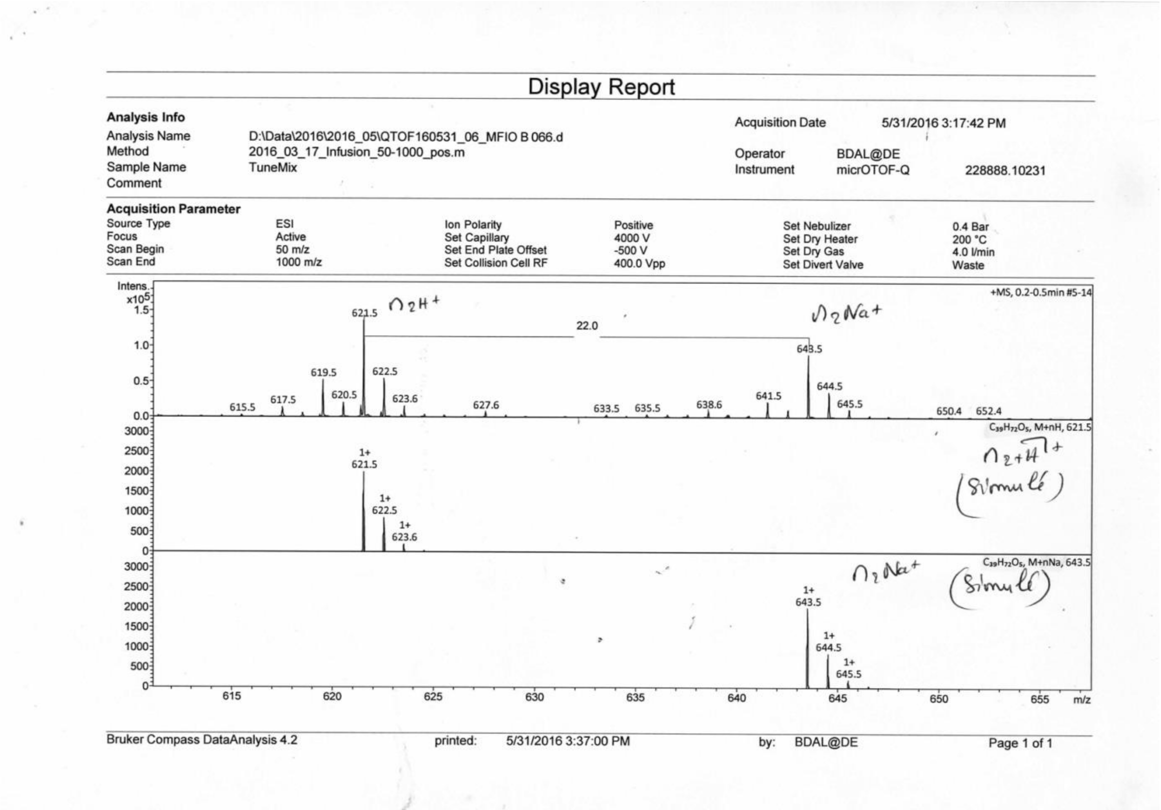

Figure S10. ESI-MS (positive ion mode) of: upper lane crude reaction mixture obtained by heating **1** + **2a** + **3a** at 80°C for 48h. Residual starting material **1**. Neat conditions gave similar results with **2a** or **2b.**

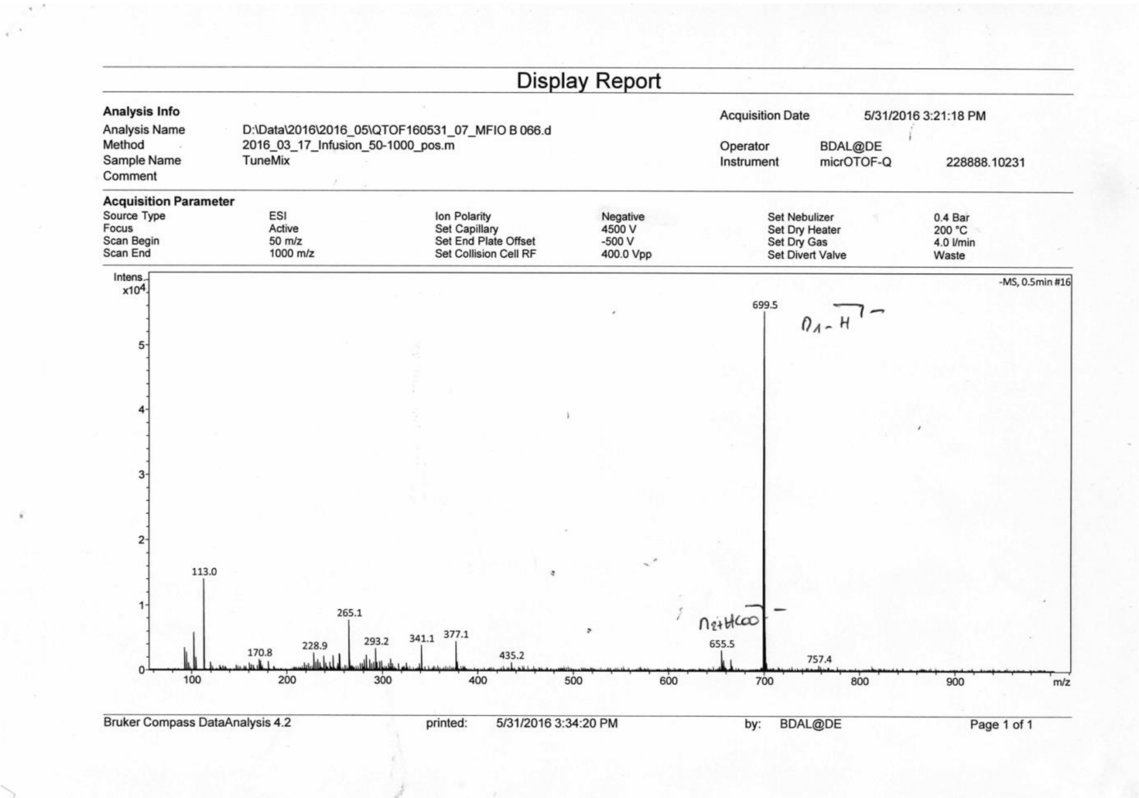

Figure S11. ESI-MS (negative ion mode) of crude reaction mixture obtained by heating **1** + **2a** + **3a** at 80°C for 48h. Fragmentation of the *rac*-DOPA (**4a**) as *m/z* 699 $[M–H]^-$. Mono-acyl glycerol phosphate (**4b**) was recorded as *m/z* 435 $[M–H]^-$.Neat conditions gave same results with **2a** or **2b.**

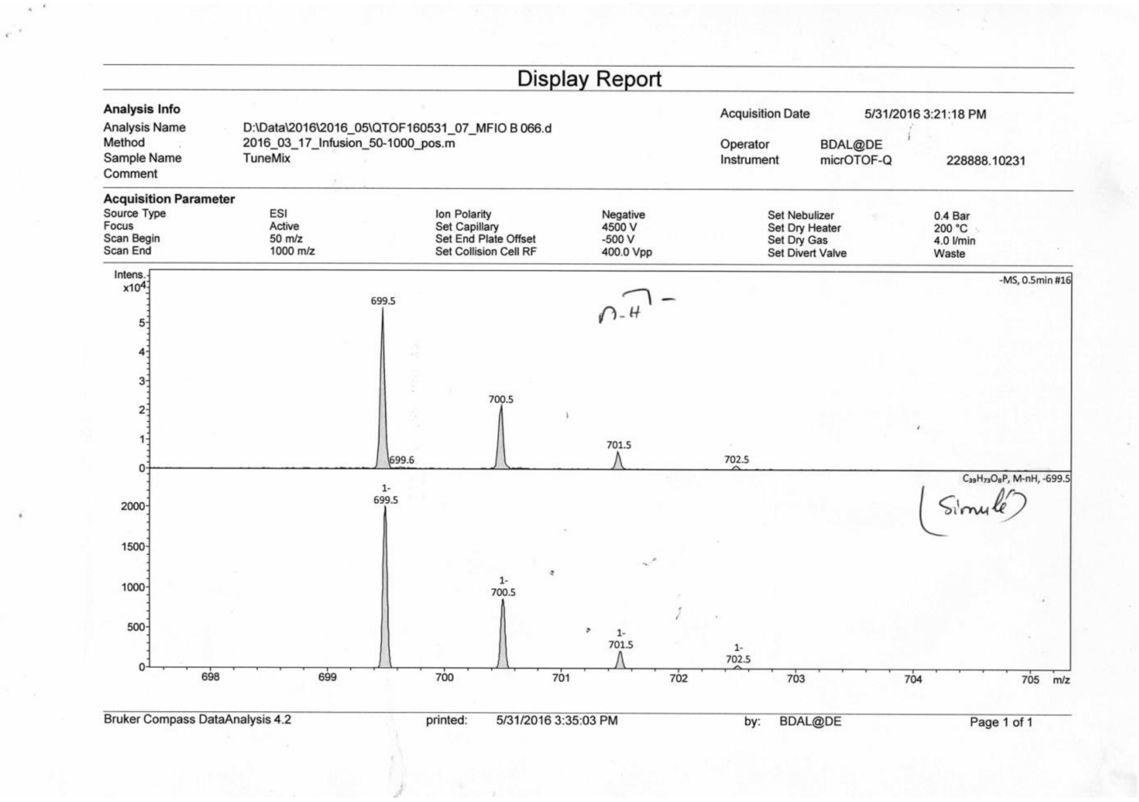

Figure S12. ESI-MS (negative ion mode) of crude reaction mixture obtained by heating **1** + **2a** + **3a** at 80°C for 48h. Fragmentation of the *rac*-DOPA (**4b**) *m/z* 699 $[M-H]^-$ and simulated spectra. Neat conditions gave same results with **2a** or **2b.**

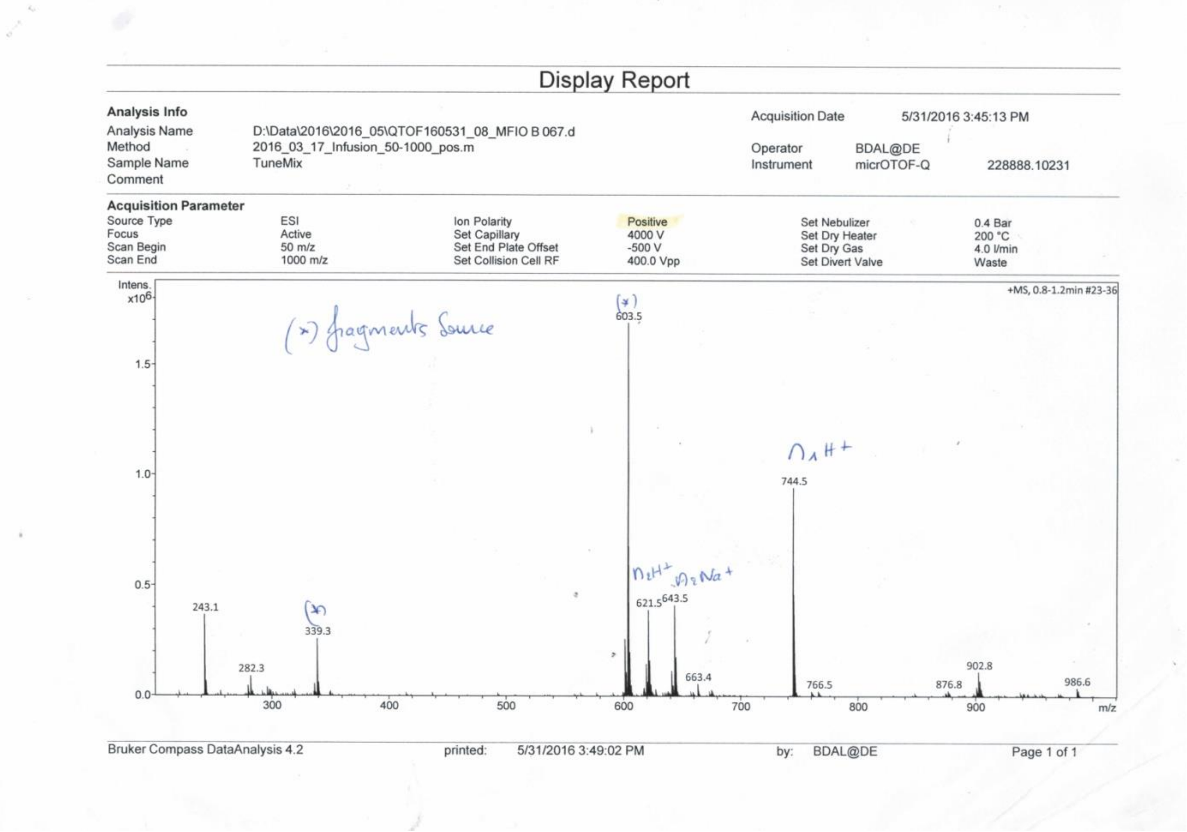

Figure S13. ESI-MS (positive ion mode) of crude reaction mixture obtained by heating **1** + **2a** + **3b** at 80°C for 48h. The use of urea (**2b**) gave similar results except the formation of **6** (*m/z* 663 $[M+H]^+$). Neat conditions gave same results with **2a** or **2b.**

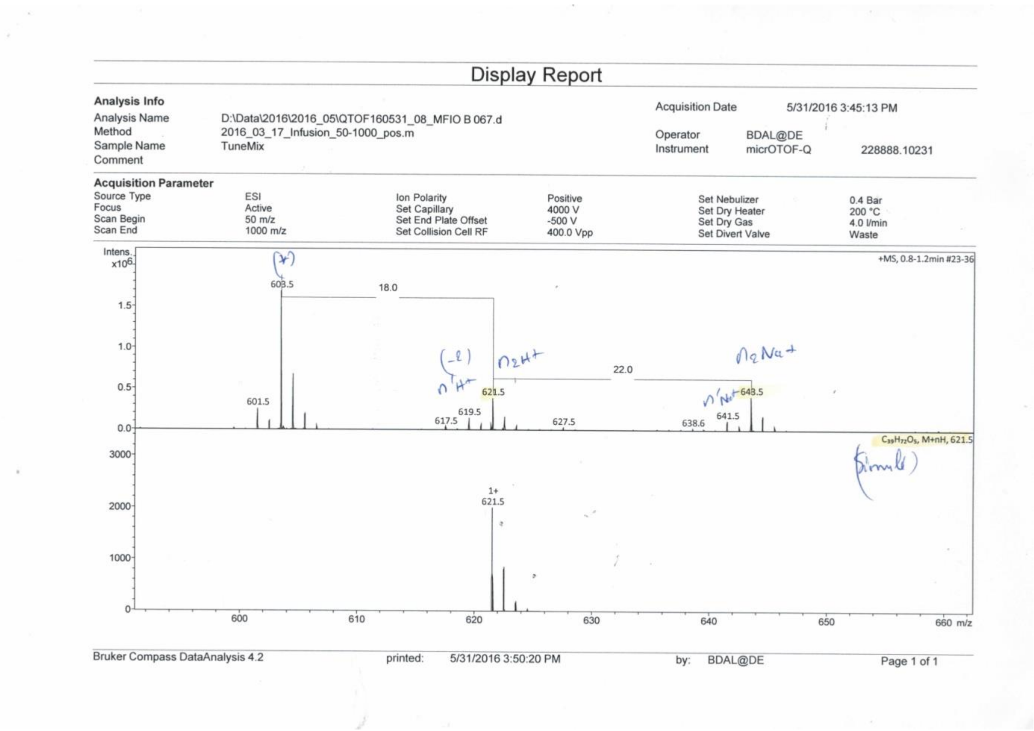

Figure S14. ESI-MS (positive ion mode) of crude reaction mixture (upper lane) obtained by heating **1** + **2b** + **3b** at 80°C for 48h. The use of cyanamide (**2a**) gave similar results. Fragmentation of the starting material **1** and simulated ESI-MS spectra are reported in the lower lane. Neat conditions gave same results with **2a** or **2b.**

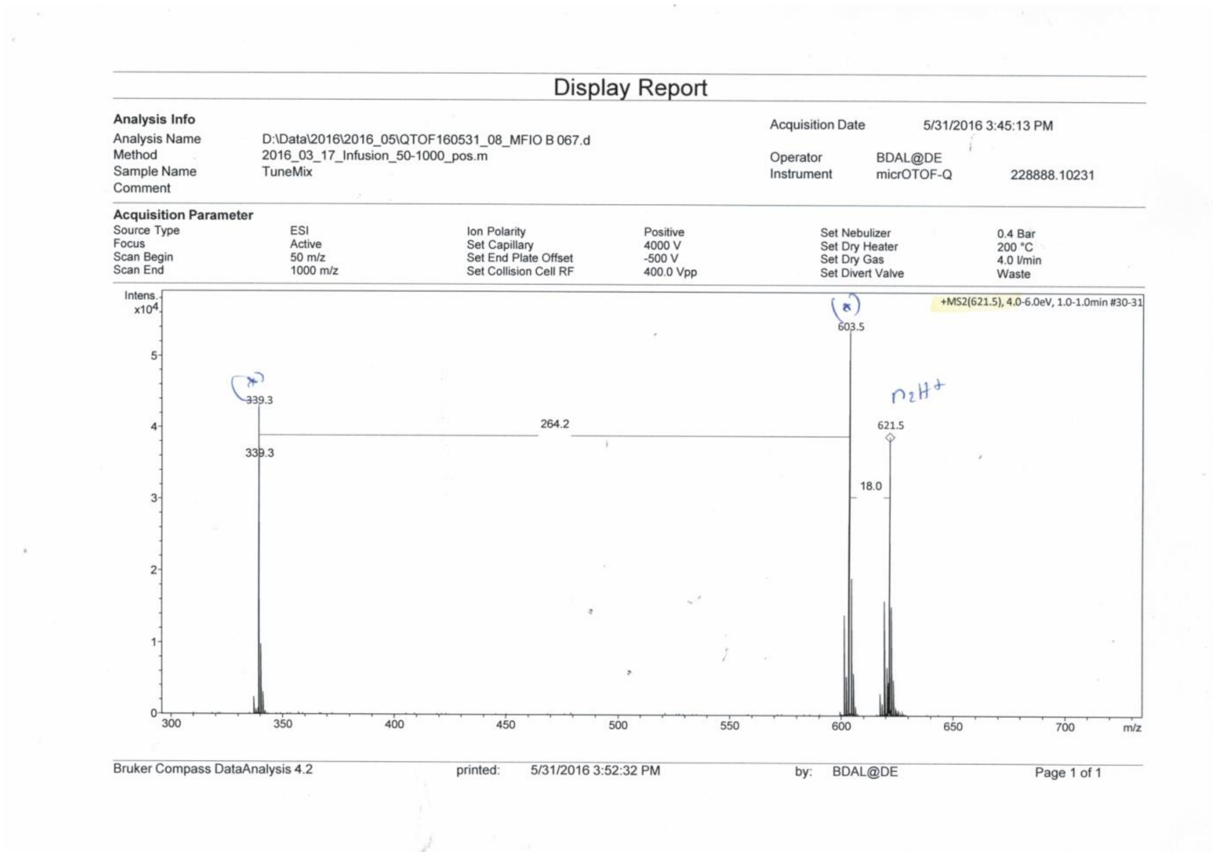

Figure S15. ESI-MS (positive ion mode) of crude reaction mixture obtained by heating **1** + **2b** + **3b** at 80°C for 48h. Fragmentation of the starting material **1**. The use of cyanamide (**2a**) gave similar results.

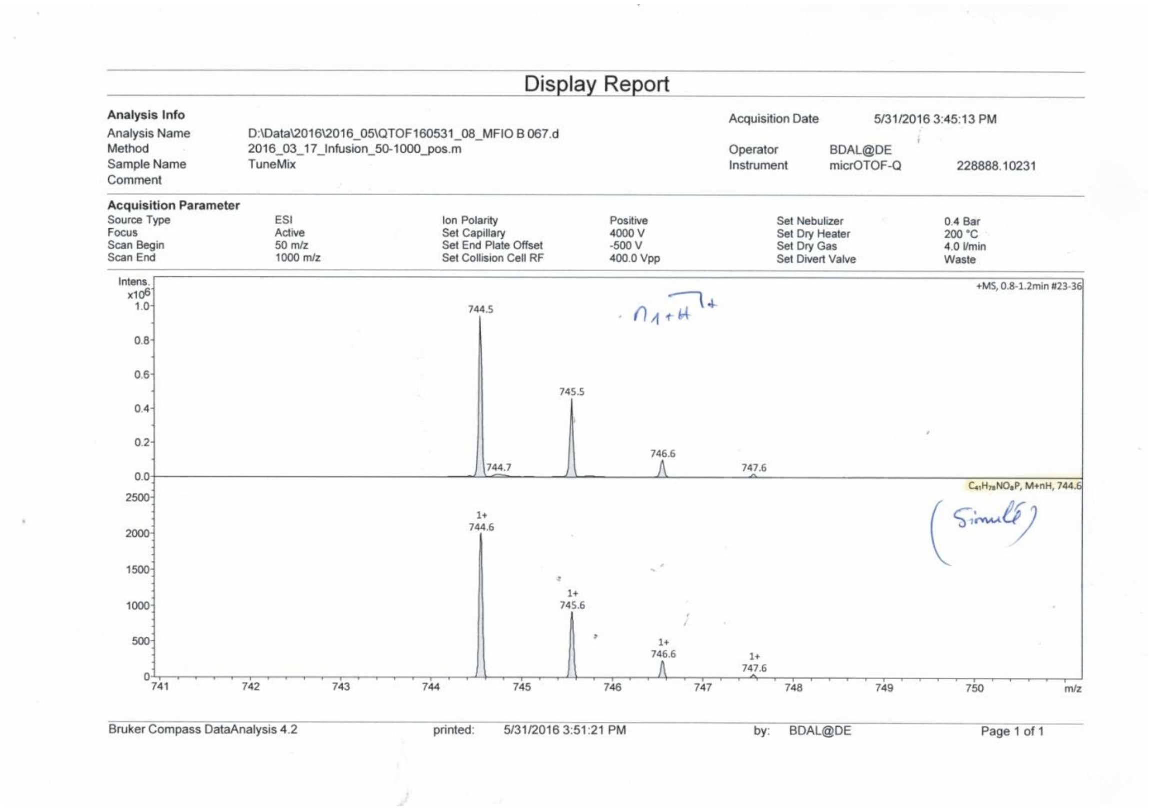

Figure S16. ESI-MS (positive ion mode, upper lane) and simulated ESI-MS spectra (down lane) of crude reaction mixture obtained by heating **1** + **2b** + **3b** at 80°C for 48h. Fragmentation of the starting material *rac*-DOPE (**5**) as m/z 744 $[M+H]^+$. The use of cyanamide (**2a**) gave similar results.

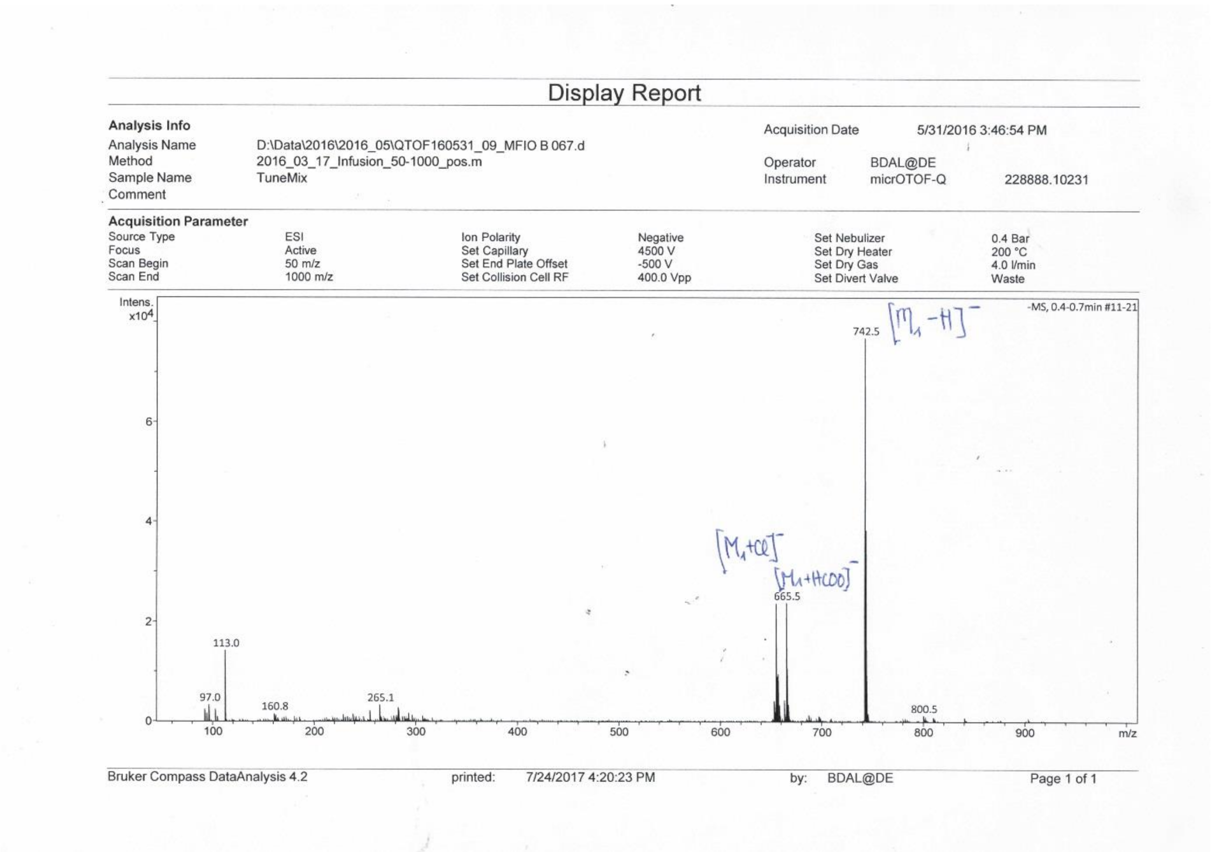

Figure S17. ESI-MS (negative ion mode) of crude reaction mixture obtained by heating **1** + **2b** + **3b** at 80°C for 48h. Compound *rac*-DOPE (**5**) detected as m/z 742 [M**–**H]$^{-}$ and residual starting material **1** as *m/z* 655 [M□Cl]$^{-}$ and *m/z* 665 [M**+**HCOO]$^{-}$.

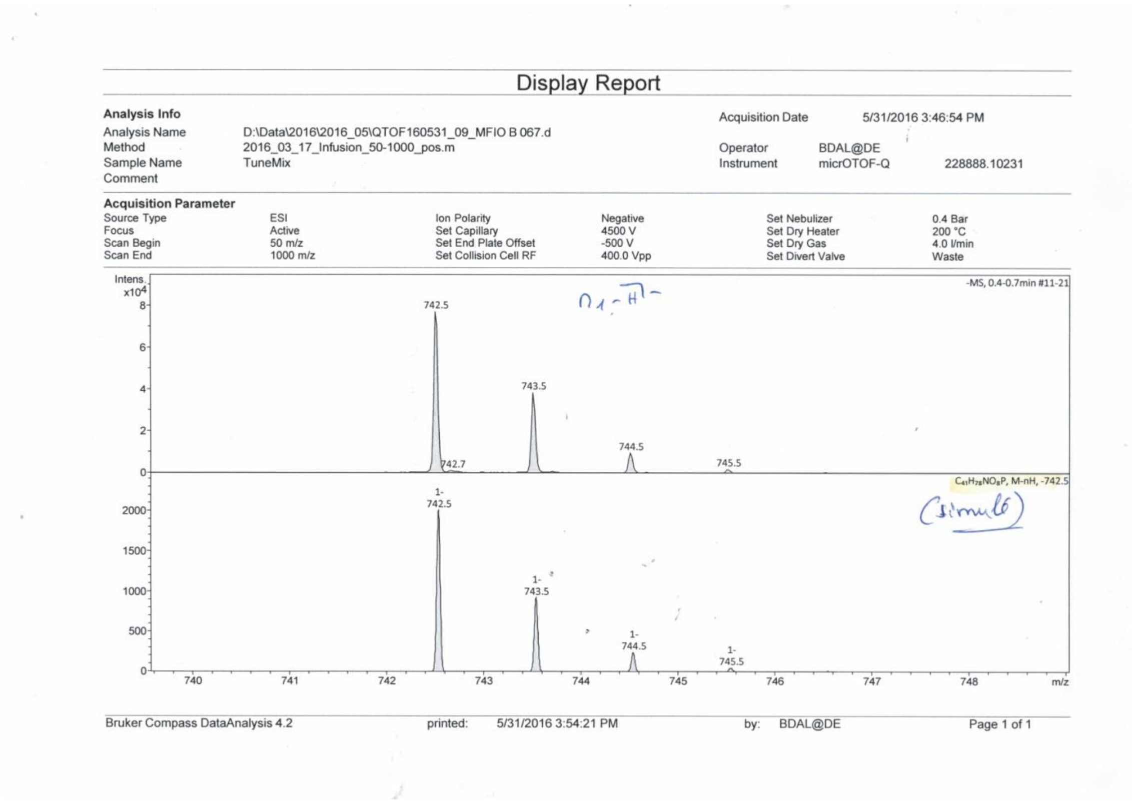

Figure S18. ESI-MS (negative ion mode, upper lane) and simulated spectrum (lower lane) of crude reaction mixture obtained by heating **1** + **2b** + **3b** at 80°C for 48h. Compound *rac*-DOPE (**5**) detected as *m/z* 742 [M–H]$^{-}$.

IV NMR spectroscopic characterization of commercial compounds

Chloroform solutions of commercial DOPA and DOPE (Avanti Lipids, Alabaster, USA) were used to this purpose. 1 mL of solution (corresponding to 25 mg) was dried under vacuum and the resulting lipidic film was dissolved in 660�L of $CDCl_3$. Oleic acid (24.6 mg, 0.1 mmol) was dissolved in 660�L of $CDCl_3$.

IV.a 1,2–Dioleoyl–*sn*–glycero–3–phosphate (commercial DOPA)

^1H NMR (500 MHz, $CDCl_3$) δ_H= 5.36 – 5.29 (m, 4H, 2 x *Z*–CH=CH), 5.24 (br s, 1H, $CH_2C\underline{H}CH_2$ glycerol), 4.40-4.37 (m, 1H, $C\underline{H}HCHCH_2$ glycerol), 4.21 (br s, 1H, $CH_2CHC\underline{H}H$ glycerol), 3.91 (br s, 2H, $CH\underline{H}CHCH\underline{H}$ glycerol), 3.6-2.9 (br, $PO_{>2}(OH)_{<2}$), 2.32-2.26 (m, 4H, 2 x $C\underline{H}_2COOR$), 2.02–1.98 (m, 8H, 2 x $C\underline{H}_2$-CH=CH-$C\underline{H}_2$), 1.57 (br s, 4H, 2 x $C\underline{H}_2CH_2COOR$), 1.38-1.22 (br s, 40H, 20 x CH_2), 0.88 (t, *J* = 10.0 Hz, 6H, 2 x CH_3).

IV.b 1,2–Dioleoyl–*sn*–glycero–3–phosphoethanolamine (commercial DOPE)

^1H NMR (500 MHz, $CDCl_3$) δ_H= 8.38 (br s, 2.5H, $NH_2 \rightleftharpoons NH_3^+$), 5.37-5.30 (m, 4H, 2 x *Z*–CH=CH), 5.24-5.18 (m, 1H, $CH_2C\underline{H}CH_2$ glycerol), 4.37 (dd, *J* = 5.0Hz, 1H, $C\underline{H}HCHCH_2$ glycerol), 4.16 – 4.10 (m, 3H, $CH\underline{H}CHC\underline{H}_2$ glycerol), 3.97 (br s, 2H, $OC\underline{H}_2CH_2NH_2$), 3.20 (br s, 2H, $OCH_2C\underline{H}_2NH_2$), 2.35–2.21 (m, 4H, 2 x $C\underline{H}_2COOR$), 2.02–1.99 (m, 8H, 2 x $C\underline{H}_2$-CH=CH-$C\underline{H}_2$), 1.60 (br s, 4H, 2 x $C\underline{H}_2CH_2COOR$), 1.39–1.22 (br s, 40H, 20 x CH_2), 0.88 (t, *J* = 10.0 Hz, 6H, 2 x CH_3).

IV.c (9*Z*)–octadec-9-enoic acid (commercial OA)

^1H NMR (500 MHz, $CDCl_3$) δ_H= 5.38 – 5.30 (m, 2H, *Z*–CH=CH), 2.35 (t, *J* = 5.0 Hz, 2H, $C\underline{H}_2COOR$), 2.03-1.99 (m, 2H, $C\underline{H}_2$-CH=CH-$C\underline{H}_2$), 1.66-1.60 (m, 2H, $C\underline{H}_2CH_2COOR$), 1.31–1.24 (br s, 20H, 10 x CH_2), 0.88 (t, *J* = 10.0 Hz, 3H, CH_3).

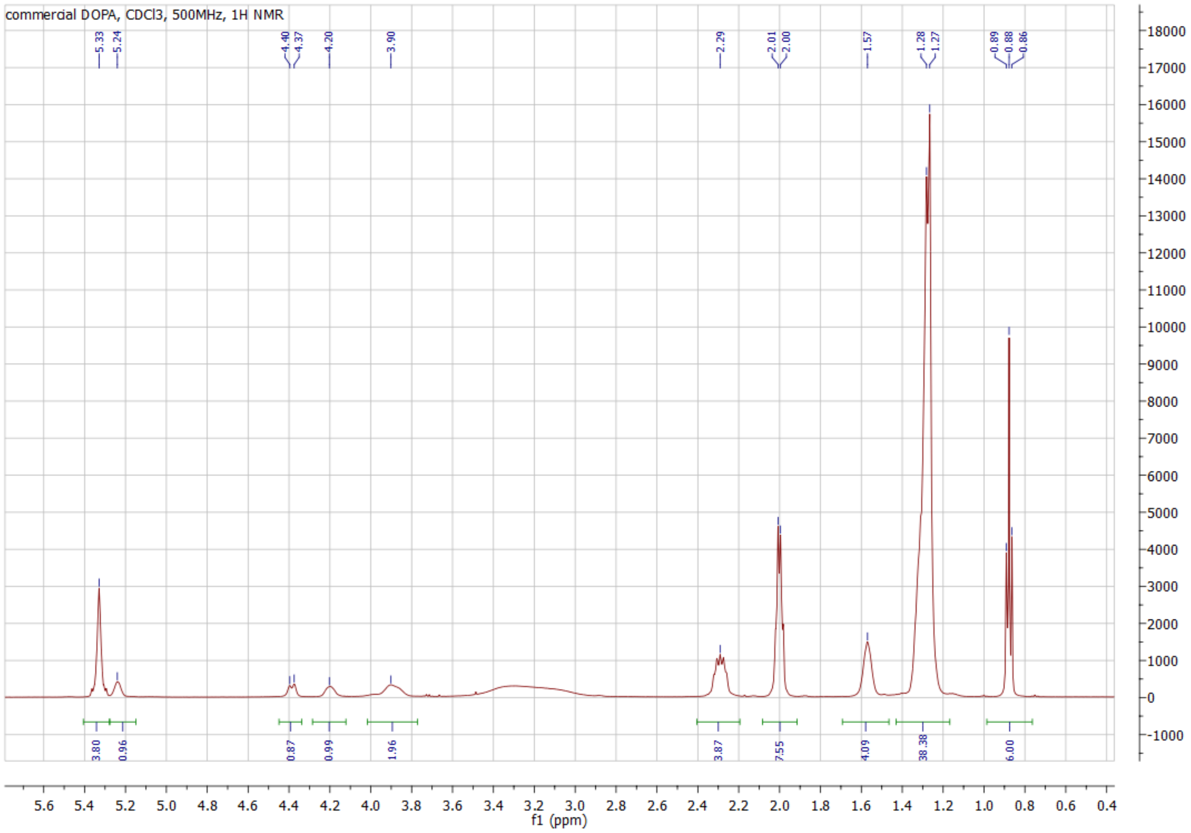

Figure S19 – ^1H NMR (500MHz, $CDCl_3$) of commercial DOPA.

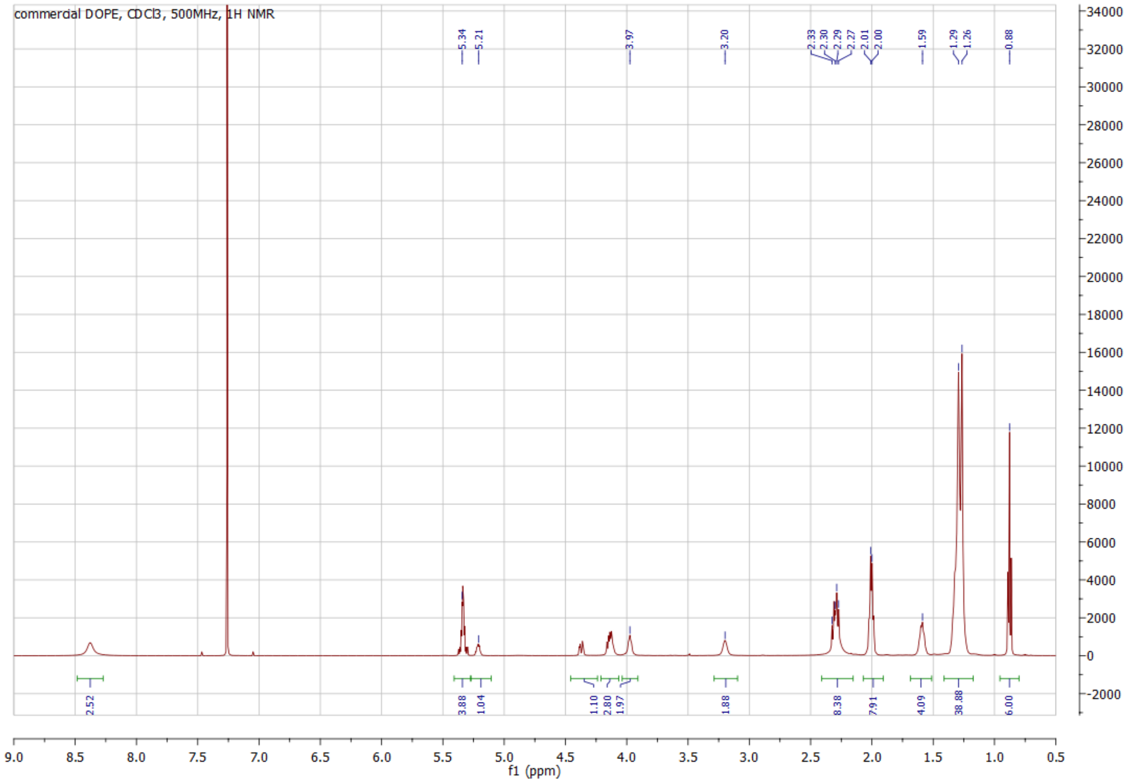

Figure S20 – ^{1}H NMR (500MHz, $CDCl_3$) of commercial DOPE.

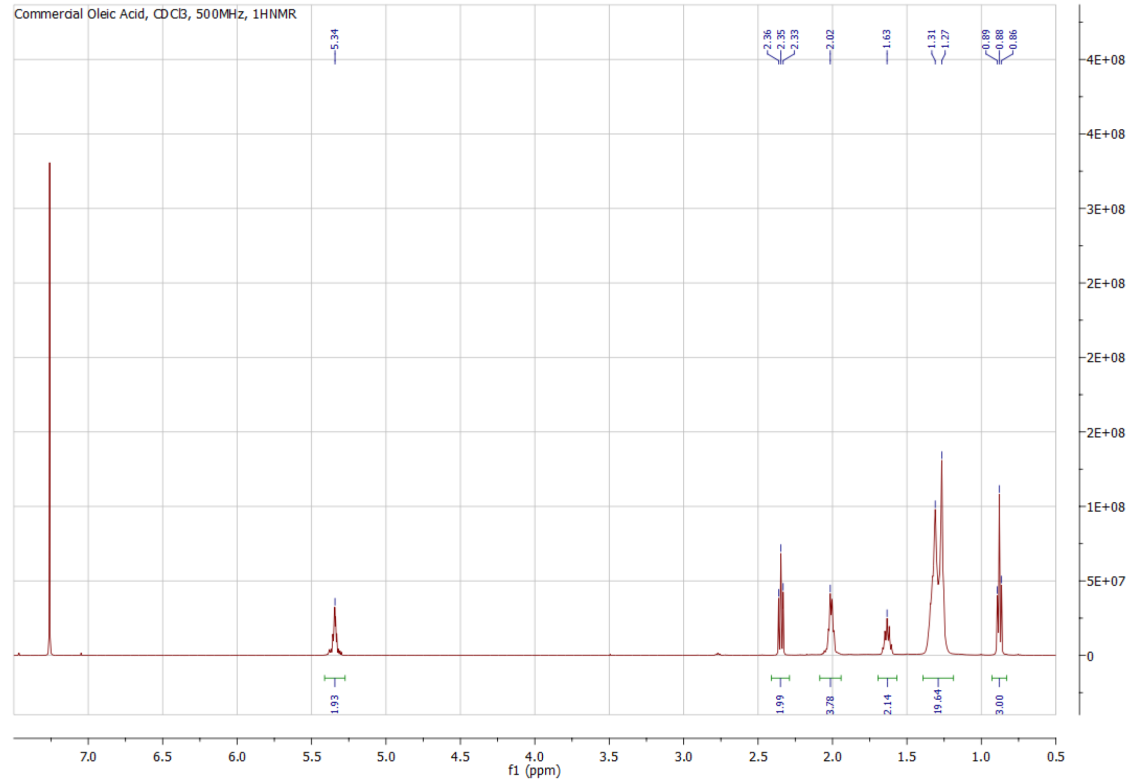

Figure S21 – ^{1}H NMR (500MHz, $CDCl_3$) of commercial oleic acid (**8**).

V Synthesis of *rac*–DOG (1) and *rac*–MOG (7a)

Racemic dioleoyl glycerol (*rac*–DOG, **1**) was obtained in three steps from commercial glycerol. Selective protection was performed with triphenylmethyl chloride (TrtCl) in the presence of a catalytic amount of 4-dimethylaminopyridine (DMAP). *Rac*3–triphenylmethyl–glycerol (**9**) was crystallized from dichloromethane/pentane and used without further purification. Subsequently, **9** was acylated with oleoyl chloride in the presence of DMAP. Compound **10** was obtained as a viscous oil and the deprotection of **10** was carried out with a catalytic amount of HCl in MeOH/$CHCl_3$ 1:1 *v/v*.

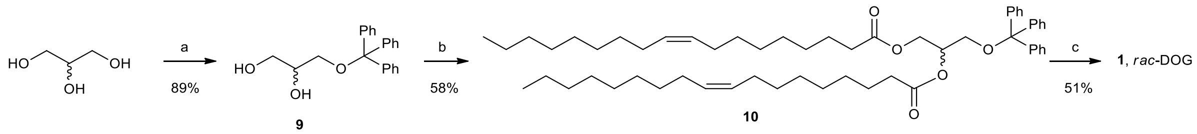

Reaction conditions: a) TrtCl, DMAP, 16h rt; b) OleoylCl, DMAP, 16h rt; c) HCl cat, 0°C, 16h

Scheme S1. Synthetic pathway of *rac*-1,2-dioleoyl-sn-glycerol (**1,** *rac*-DOG)

Racemic mono-oleoyl glycerol (*rac*–MOG, **7a**) was obtained in two steps from commercial [illegible],[illegible]-isopropylidene-DL-glycerol. Acylation was performed as described for the synthesis of compound **10**. The compound **11** was deprotected by using a small amount of acidic resin (Amberlyst® 15) and the product was obtained after filtration and evaporation. This kind of synthesis represents a novelty with respect to other syntheses reported in the literature for obtaining these type of compounds, including an enzymatic synthesis.

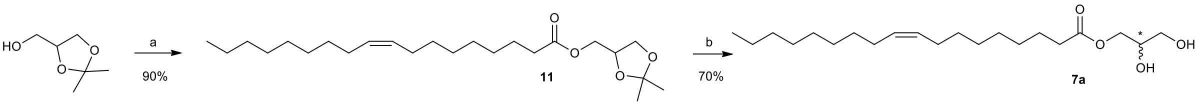

a) OleoylCl, DPAP, 16h rt; b) CF3COOH 80%

Scheme S2. Synthetic pathway of *(2R/S)*-2,3-dihydroxypropyl-1-oleate (**7a**, *rac*-MOG).

V.a 3–*O*–Triphenylmethyl–DL–glycerol (9)

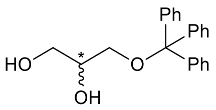

To a stirred solution of gycerol (10.0 g, 109.6 mmol), DMAP (0.075g, 0.6 mmol) and trityl chloride (7.5 g, 26.9 mmol) in 20 mL of anhydrous THF at 0 °C were added 4.5 mL of anhydrous triethylamine. The reaction mixture was stirred at r. t. overnight. A solution of $NaHCO_3$ (2.0 g in 50mL of H_2O) was added followed by stirring for 15min. The product was then extracted with ethyl acetate (2 × 35 mL). The combined organic phases were washed with brine (2 × 50 mL) and dried over anhydrous Na_2SO_4. The crude material obtained after evaporation of the solvent was crystallized from dichloromethane/pentane to give 32.57 g (89.7%) **9** as a white powder. R_f (hexane/EtOAc 1:1) 0.42; ^1H NMR (300 MHz, $CDCl_3$): δ_H= 7.57–7.07 (m, 15H, 3 x Ph), 3.92–3.73 (m, 1H, $CH_2C\underline{H}CH_2$), 3.68-3.63 (m, 2H, $CH_2CHC\underline{H}_2$), 3.36–3.18 (m, 2H, $C\underline{H}_2CHCH_2$).

V.b 1,2-*O,O*-Dioleoyl-DL-glyceryl-3-*O*-triphenylmethyl ether (10)

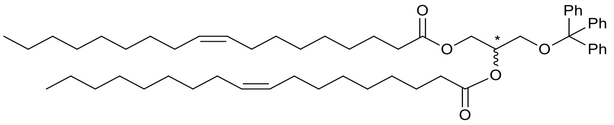

To a stirred solution of **9** (2.23 g, 6.7 mmol) in 50 mL of $CHCl_3$ was added oleoyl chloride (5.03 g, 5.52 ml, 16.7 mmol) and DMAP (2.06 g, 16.7 mmol). The resulting solution was stirred overnight at room temperature. The excess of oleoyl chloride was decomposed by addition of 50 ml solution of $NaHCO_3$ (0.4 M) and the resulting bisphasic solution was left stirring for 15 min. The biphasic solution was extracted with $CHCl_3$ (2 × 50 mL), and the combined organic phases were washed with 2 × 10 mL of brine and dried over Na_2SO_4. Evaporation of the solvent followed by chromatography over freshly activated SiO_2 with $CHCl_3$ gave 3.40 g (3.39 mmol, 58.2%) **10** as a white solid. R_f (hexane/EtOAc 4:1) 0.36; ^{1}H NMR (300 MHz, $CDCl_3$): δ_H= 7.40–7.11 (m, 15H, 3 x Ph), 5.27 (m, 4H, 2 x *Z*-CH=CH), 5.21–5.17 (m, 1H, $CH_2C\underline{H}CH_2$, glycerol), 4.33–4.01 (m, 2H, $\underline{CH_2}CHCH_2$, glycerol), 3.16 (m, 2H, $CH_2CHC\underline{H}_2$, glycerol), 2.34–2.21 (m, 4H, 2 x $C\underline{H}_2COOR$), 2.02–1.85 (m, 8H, 2 x $C\underline{H}_2$-CH=CH-$C\underline{H}_2$), 1.62–1.42 (2 x br, 4H, 2 x $C\underline{H}_2CH_2COOR$), 1.21 (s, 40H, 20 x CH_2), 0.81 (t, *J*= 8.0 Hz, 6H, 2 x CH_3).

V.c 1,2-*O,O*-Dioleoyl-DL-glycerol (1)

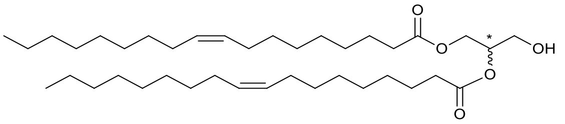

A solution of $CHCl_3$-MeOH containing 0.22 ml of concentrated HCl (37%) was cooled to 0°C and to this a solution, prepared by dissolving 2g of **9** (2.31 mmol) in 100 ml of $CHCl_3$-MeOH solution 1:1 *v/v*, was added drop wise during 6hrs. The clear solution was left stirring at 4°C overnight. A saturated solution of $NaHCO_3$ was added slowly (15 minutes) and the resulting heterogeneous biphasic solution was left stirring up to room temperature. The resulting solution was extracted with $CHCl_3$ (3× 250 ml), and the combined organic phases was washed with 3× 100 mlof brine and dried over Na_2SO_4. Evaporation of the solvent followed by chromatography over freshly activated SiO_2 with $CHCl_3$ gave 0.70 g (51%) **1** as pale yellow oil. R_f (2:1 hexane/EtOAc) 0.30; ^{1}H NMR (500 MHz, $CDCl_3$): δ_H= 5.40-5.28 (m, 4H, 2 x *Z*-CH=CH), 5.08 (quint, 1H, *J* = 5.0 Hz, C(2)H), 4.31 (dd, *J* = 11.9, 4.6 Hz, 1H, C(1)H_b), 4.24 (dd, *J* = 11.9, 4.6 Hz, 1H, C(1)H_a), 3.74 (dd, *J* = 12.2, 4.7 Hz, 1H, C(3)H_b), 3.72 (dd, *J* = 12.2, 4.7 Hz, 1H, C(3)H_a), 2.39-2.37 (2 x t, *J* = 7.5 Hz, 4H, 2 x $C\underline{H}_2COOR$), 2.08-1.90 (m, 8H, 2 x $C\underline{H}_2$-CH=CH-$C\underline{H}_2$), 1.67-1.56 (m, 4H, 2 x $C\underline{H}_2CH_2COOR$), 1.30, 1.26 (2 x br, 40H, 20 x CH_2), 0.87 (t, *J* = 6.8 Hz, 6H, 2 x CH_3). ^{13}C NMR (75MHz, $CDCl_3$): δ_C= 14.1 (CH_3), 22.1 (CH_2), 24.8 (CH_2), 24.9 (CH_2), 25.6 (CH_2), 27.0 (CH_2), 27.1 (CH_2), 27.2 (CH_2), 29.0-29.2 (4xCH_2), 29.3 (CH_2), 29.5 (CH_2), 29.7 (CH_2), 29.8 (CH_2), 31.9 (CH_2), 34.0 (CH_2), 34.3 (CH_2), 61.5 (C3), 62.0 (C1), 72.2 (C2), 129.8 (*Z*-$\underline{C}$H=CH), 130.0 (*Z*-CH=$\underline{C}$H), 173.5 (C=O), 173.9 (C=O); $[\alpha]_D^{25}$ = 0.00 (*c* 0.1, $CHCl_3$). ESI-MS *m/z* 643 as M+Na^+; HRMS (*m/z*): $[M]^+$ calcd. for $C_{39}H_{72}NO_5$: 634.5410, found $C_{39}H_{72}NNaO_5$: 643.5272.

V.d *(4R/S)*-(2,2-Dimethyl-1,3-dioxolan-4-yl)methyl oleate (11)

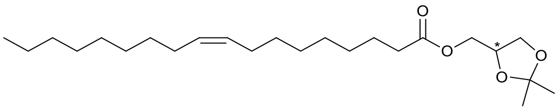

2.15 gr (15.13 mmol, 1 eq) of commercial DL-[illegible],[illegible]-isopropylideneglycerol were dissolved in 50 mL of CH_2Cl_2 and 2.31 gr (18.9 mmol, 1.25 eq) of 4-dimethylaminopyridine were added portiowise. The obtained solution was let stirring for 48hrs. A saturated solution of $NaHCO_3$ was added slowly (15 minutes) and the resulting heterogeneous biphasic solution was extracted with $CHCl_3$ (2× 50 ml), and the combined organic phases was washed with 2× 50 m of brine and dried over Na_2SO_4. Evaporation of the solvent followed by chromatography over freshly activated SiO_2 with petrol ethers/AcOEt 3:1 (Rf=0.63) gave 5.40 g (90%) pure **11** as colorless oil. ^{1}H NMR (300 MHz, $CDCl_3$): δ_H= 5.39-5.25 (m, 2H, *Z*-CH=CH), 4.30 (dt, *J* = 10.9, 6.2 Hz, 1H, C(4)), 4.19-4.04 (m, 3H, $RCOOCH\underline{H}_b$, $RCOOC\underline{H}_aH$, C(5)H_b), 3.72 (dd, *J* = 8.4, 6.2 Hz, 1H; C(5)H_a), 2.33 (t, *J* = 6.0 Hz, 2H, $C\underline{H}_2COOR$), 2.08-1.90 (m, 4H, $C\underline{H}_2$-CH=CH-$C\underline{H}_2$), 1.74-1.52 (m, 2H, $C\underline{H}_2CH_2COOR$), 1.42, 1.36 (2 x s, 6H, $(CH_3)_2C(2)$), 1.29 (m, 18H, 9 x CH_2), 0.89 (t, *J* = 8.0 Hz, 3H, CH_3). ^{13}C NMR: δ_C= 14.1 (CH_3), 22.7 (CH_2), 24.8 (CH_2), 25.4 (CH_2), 26.7 (CH_2), 27.1 (CH_2), 27.2 (CH_2), 29.1

(CH_2), 29.2 (CH_2), 29.3 (CH_2), 29.5 (CH_2), 29.7 (CH_2), 29.8 (CH_2), 31.9 (CH_2), 34.1 (CH_2), 34.3 (CH_2), 64.5 (COO<u>C</u>H_2), 66.3 (C5), 73.7 (C4), 109.8 (C2), 129.7 (Z-<u>C</u>H=CH), 130.0 (Z-CH=<u>C</u>H), 173.6 (C=O); $[\alpha]_D^{25}$ = 0.00 (*c* 0.1, $CHCl_3$).

V.e *(2R/S)*-2,3-dihydroxypropyl-1-oleate (7a)

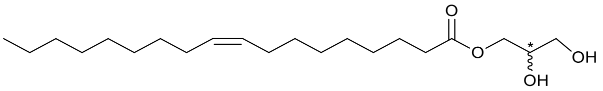

One gramme of **11** (2.5 mmol) were dissolved in MeOH (25 ml) and 2.5 gr of Amberlyst® 15 were added in 3 portions. The heterogeneous solution was let stirring overnight at room temperature. TLC analysis (petrol ethers:AcOEt 3:1) showed the total disappearance of starting material with the appearance of a more polar compound at R_f 0.30. The solution was filtered off and the residual solid washed 5 times with MeOH. After MeOH removal the yellowish solid was filtered thought a pad of fresh dried SiO_2 using petrol ethers/AcOEt 1:1 as eluent yielding 0.62 gr (1.7 mmol, 70%) **7a** (containing 7 mol% **7b**) as colorless oil. R_f (petrol ethers/AcOEt 1:1)=0.45; ^{1}H NMR (500 MHz, $CDCl_3$): δ_H= 5.33-5.24 (m, 2H, *Z*-CH=CH), 4.21 (dd, *J* = 11.7, 4.6 Hz, 1H, C(1)H_b), 4.15 (dd, *J* = 11.7, 4.6 Hz, 1H, C(1)H_a), 3.95-390 (m, 1H, C(2)H), 3.70 (dd, *J* = 11.4, 4.0 Hz, 2H; C(3)H_b), 3.60 (dd, *J* = 11.4, 4.0 Hz, 2H; C(3)H_a), 2.33 (t, *J* = 7.7 Hz, 2H, C<u>*H*</u>$_2$COOR), 2.05-1.90 (m, 4H, C<u>*H*</u>$_2$-CH=CH-C<u>*H*</u>$_2$), 1.63-1.50 (m, 2H, C<u>*H*</u>$_2$$CH_2$COOR), 1.30, 1.27 (2 x br, 20H, 10 x CH_2), 0.89 (t, *J* = 7.6 Hz, 3H, CH_3).^{13}C NMR: δ_C= 14.1 (CH_3), 22.7 (CH_2), 24.9 (CH_2), 27.1 (CH_2), 27.2 (CH_2), 29.0 (CH_2), 29.1 (CH_2), 29.2 (CH_2), 29.3 (CH_2), 29.5 (CH_2), 29.7 (CH_2), 29.8 (CH_2), 31.9 (CH_2), 34.1 (CH_2), 63.4 (C3), 65.1 (C1), 70.3 (C2), 129.7 (Z-<u>CH</u>=CH), 130.0 (Z-CH=<u>C</u>H), 174.3 (C=O); [⍰]$_D^{25}$ = 0.00 (*c* 0.1, $CHCl_3$). ESI-MS *m/z* 379.3 as $[M+Na]^+$.

7b. (300MHz, $CDCl_3$) : δ_H= 4.92 (quint, 1H, *J* = 4.7 Hz), 3.83 (d, 1H, *J*= 5.7 Hz); other signals are masked under those of **7a**

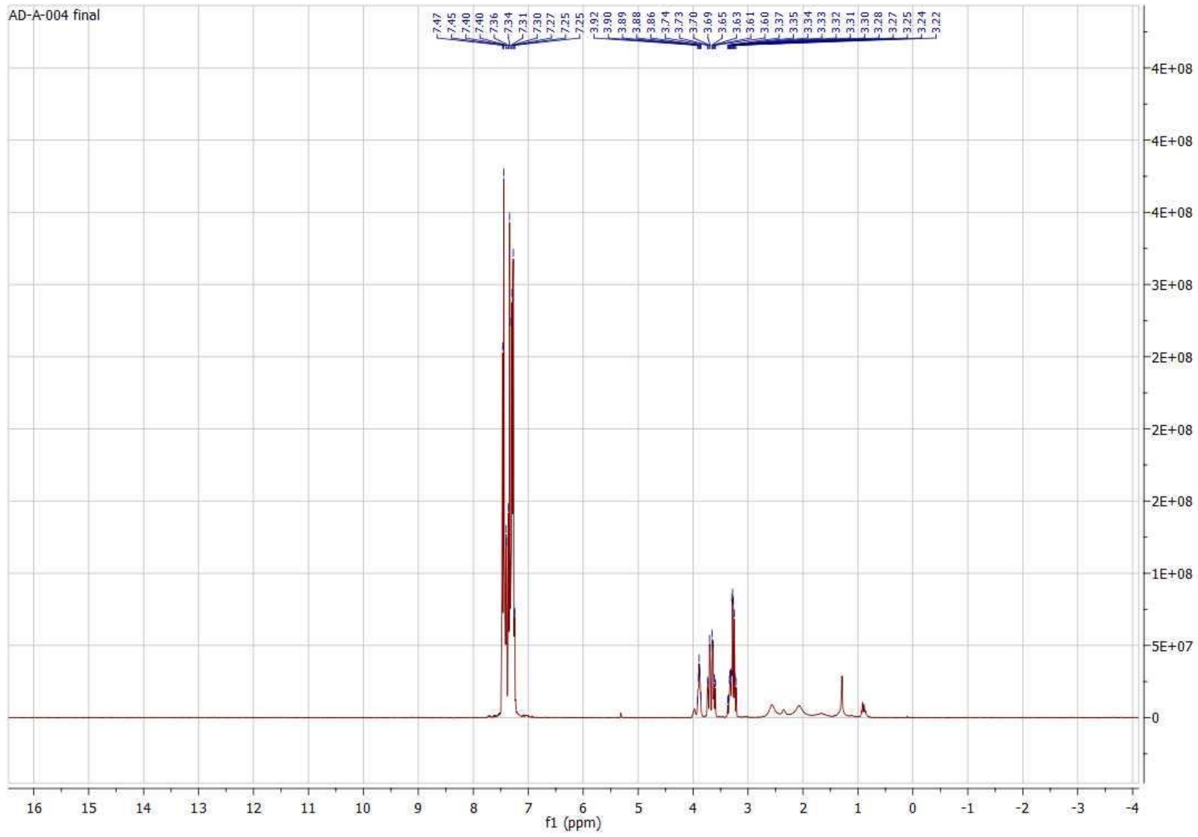

Figure S22. ^{1}H NMR (300 MHz, $CDCl_3$) of product **9**.

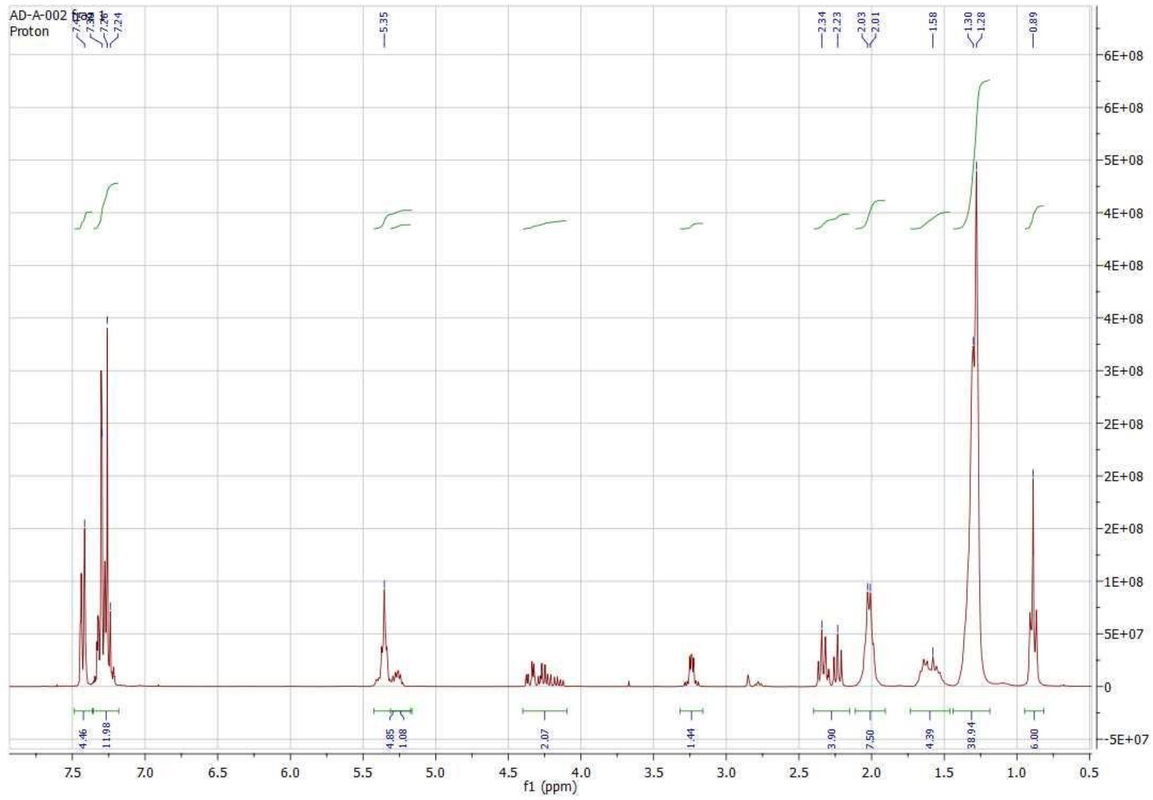

Figure S23. ^{1}H NMR (300MHz, $CDCl_3$) of product **10**.

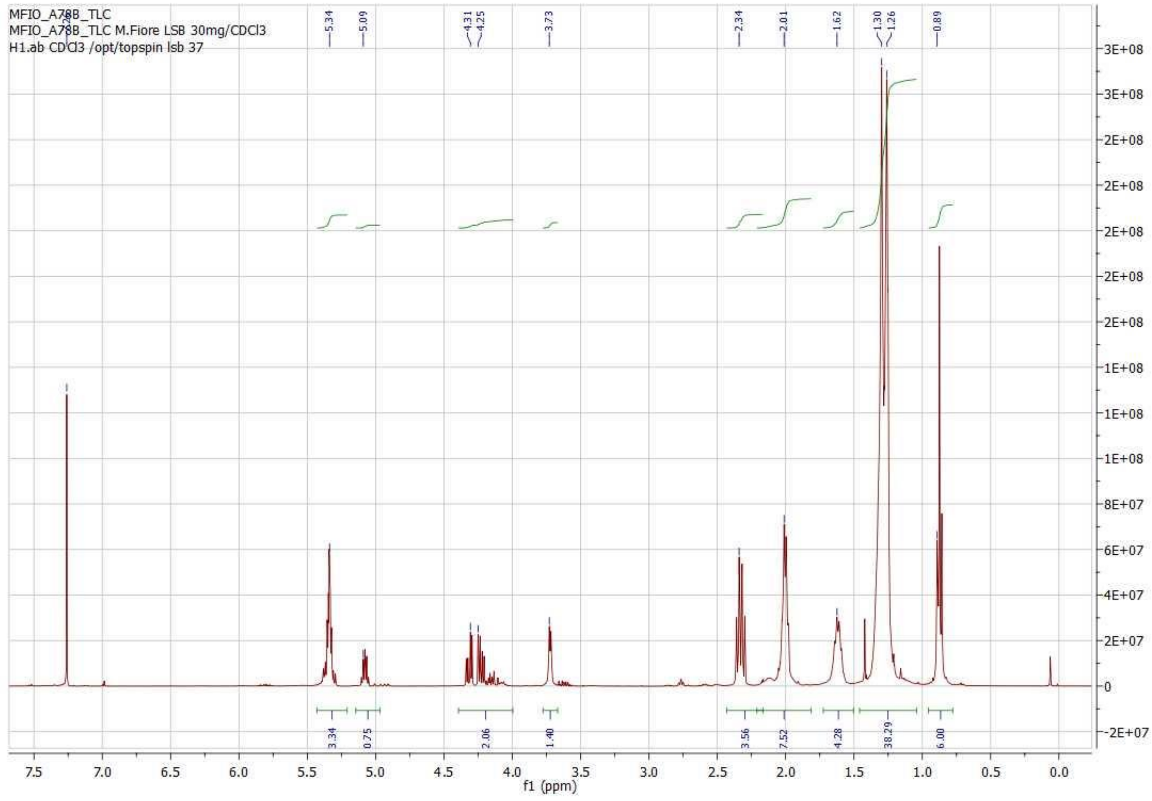

Figure S24 – ^{1}H NMR (300MHz, $CDCl_3$) of *rac*-DOG **1**.

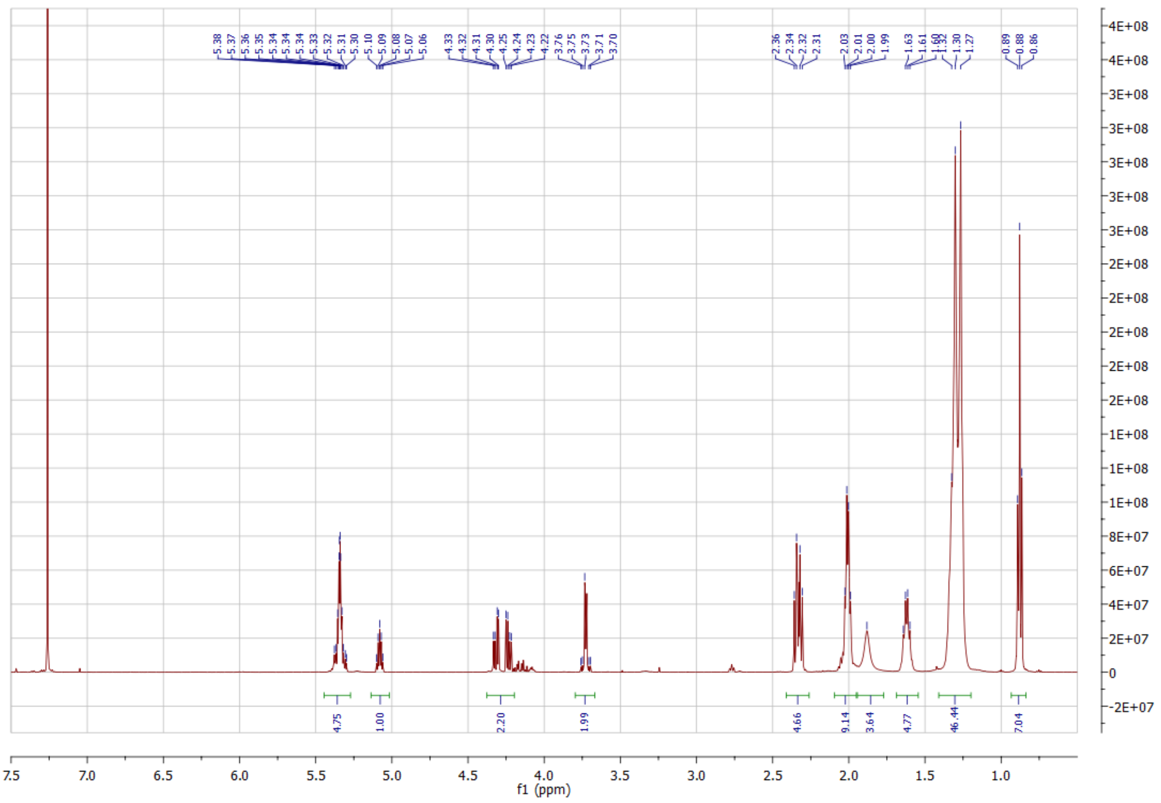

Figure S25. ^{1}H NMR (500MHz, $CDCl_3$) of *rac*-DOG **1**.

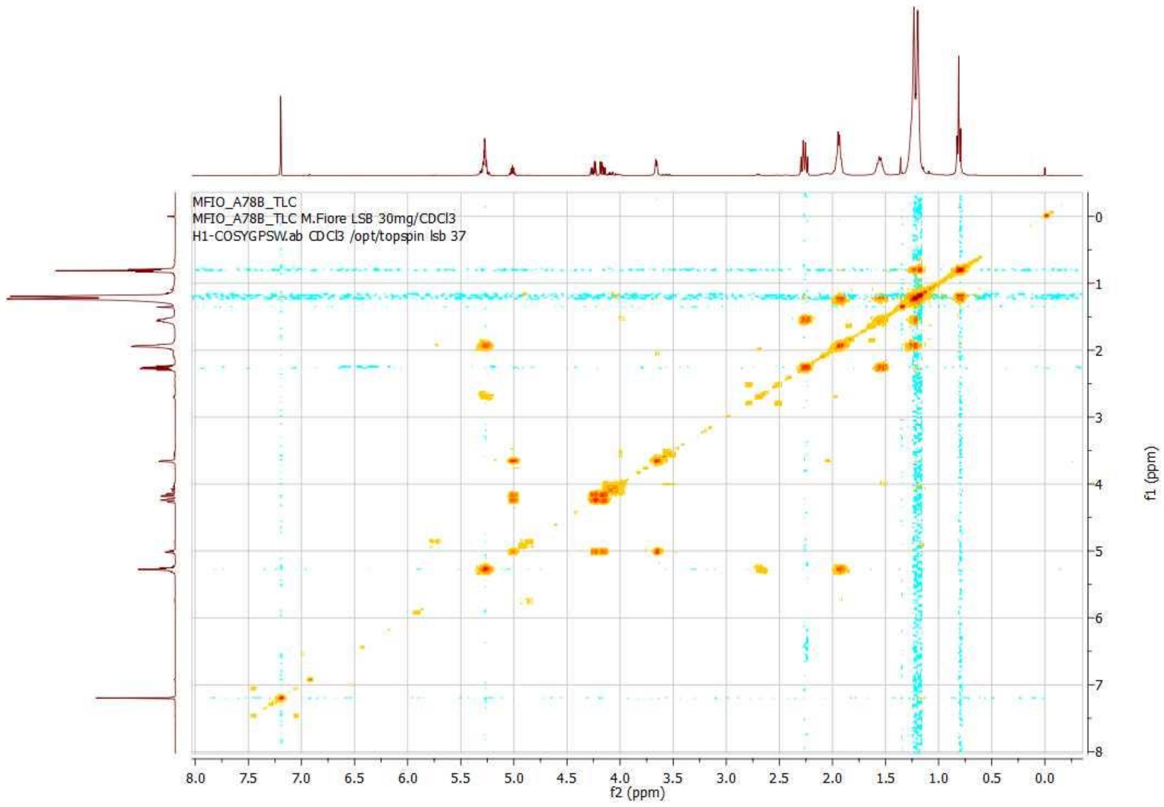

Figure S26. COSY (300 MHz, $CDCl_3$) of **1**.

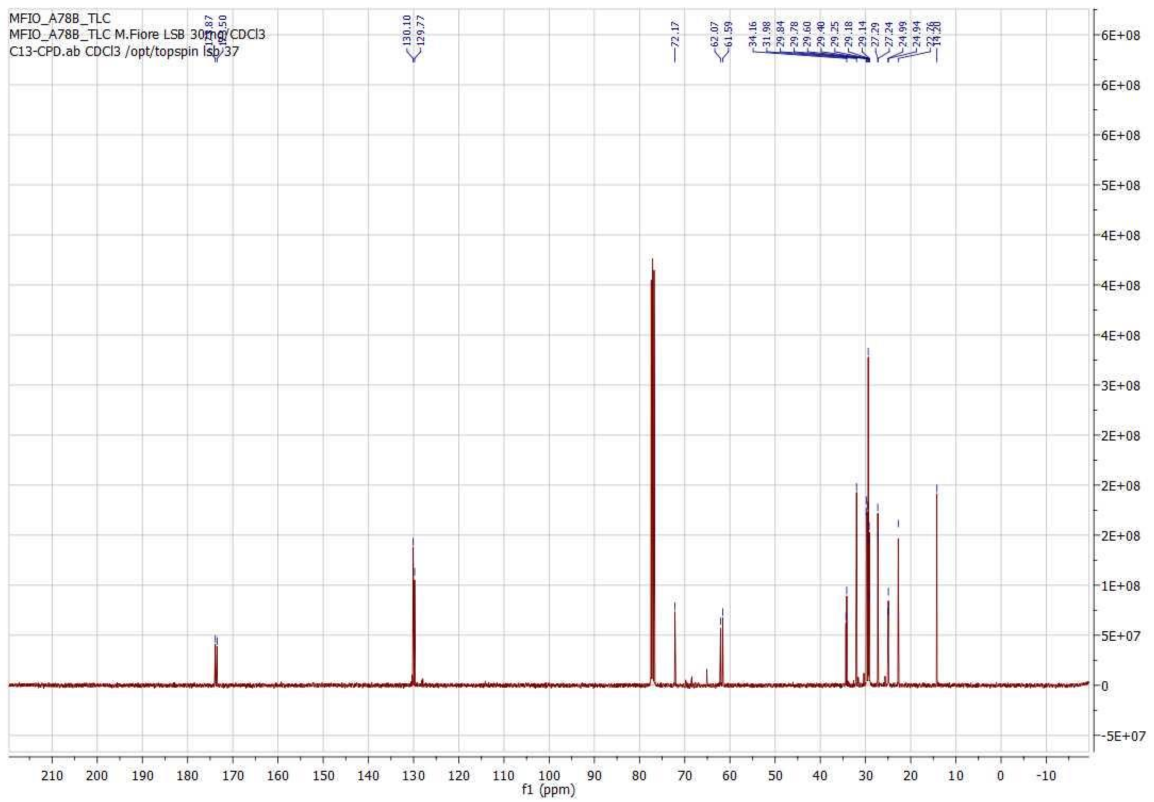

Figure S27. ^{13}C NMR (75 MHz, $CDCl_3$) of **1**.

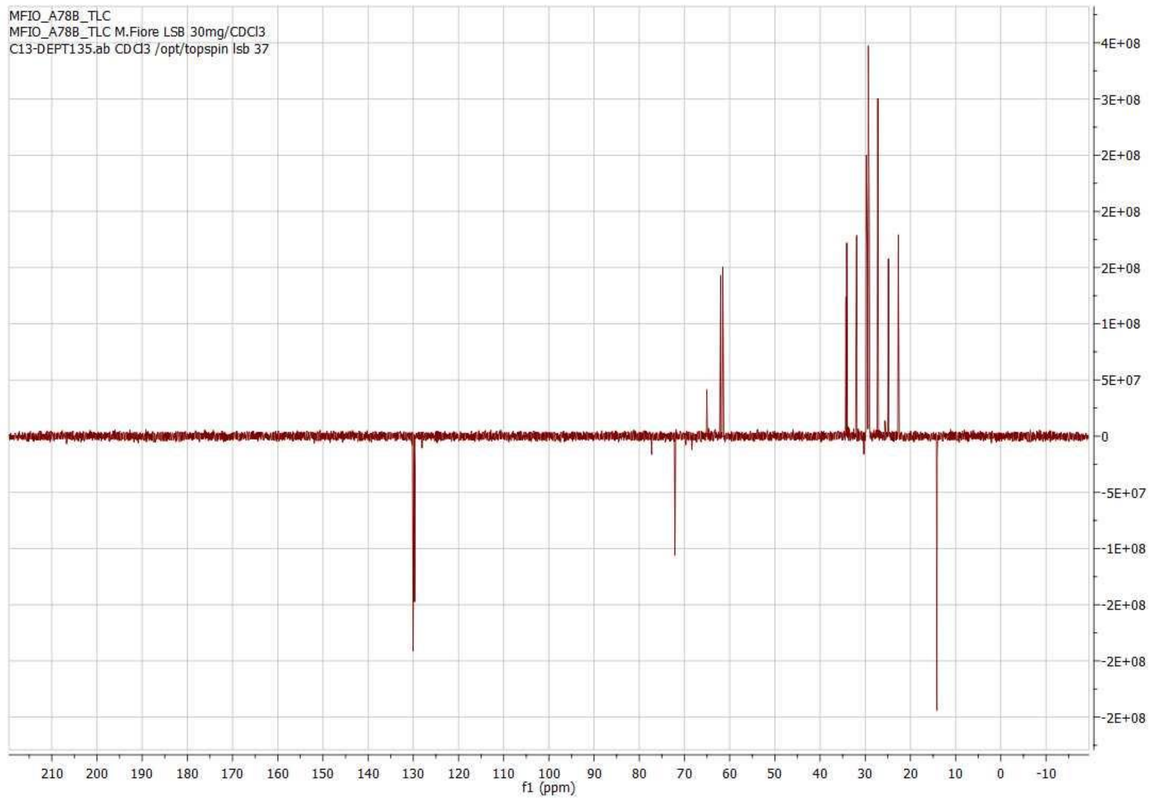

Figure S28. DEPT (75 MHz, $CDCl_3$) of **1**.

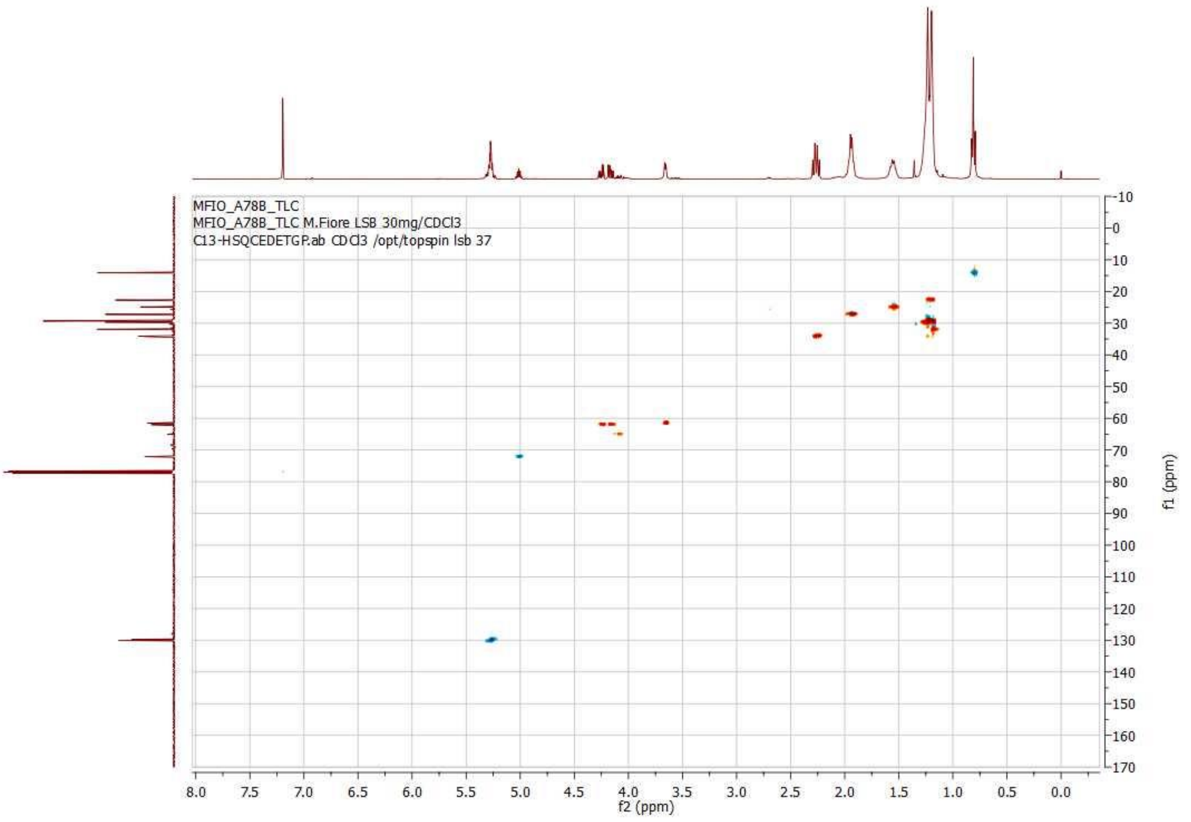

Figure S29. HSQC (75 MHz, $CDCl_3$) NMR of **1**.

CENTRE COMMUN DE SPECTROMETRIE DE MASSE

Analysis Info

Analysis Name	QTOF160523_03_WM_B_061_B.d		
Method	2016_03_17_Infusion_50-1000_pos.m	Acquisition Date	5/23/2016 3:00:30 PM
Comment		Instrument / Ser#	micrOTOF-Q 228888.10231

Acquisition Parameter

Source Type	ESI	Ion Polarity	Positive	Set Nebulizer	0.4 Bar
Focus	Active	Set Capillary	1200 V	Set Dry Heater	200 °C
Scan Begin	50 m/z	Set End Plate Offset	-500 V	Set Dry Gas	4.0 l/min
Scan End	1000 m/z	Set Collision Cell RF	400.0 Vpp	Set Divert Valve	Waste

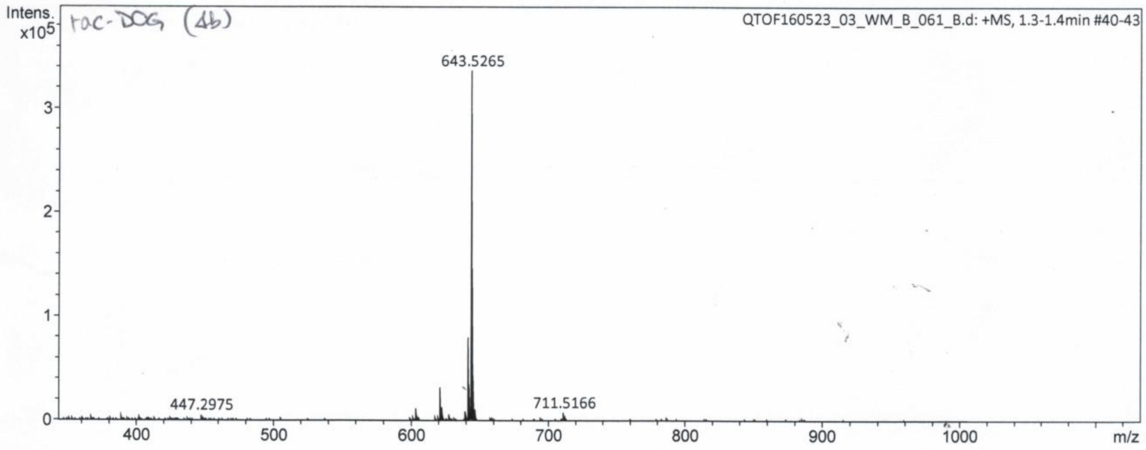

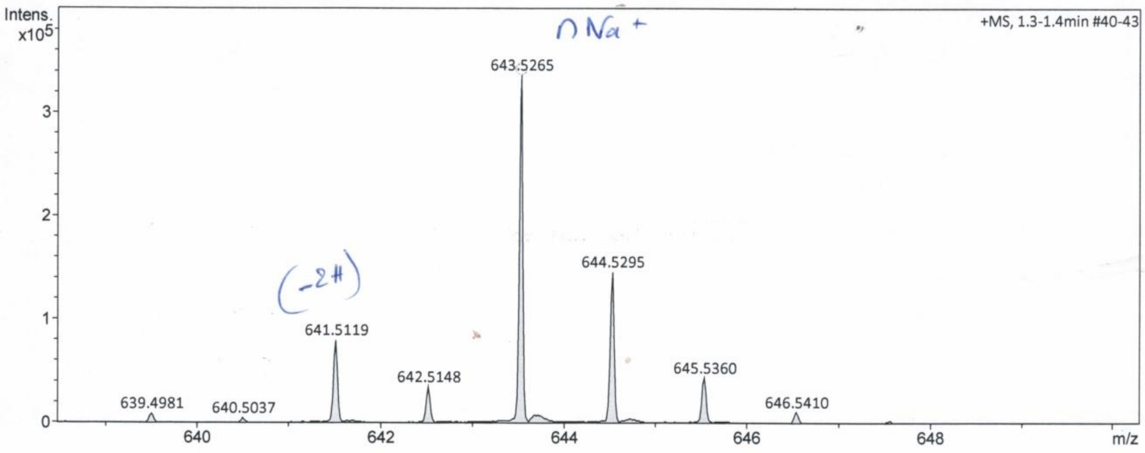

Meas. m/z	Ion Formula	m/z	err [ppm]	mSigma
643.5265	C39H72NaO5	643.5272	1.1	15.9

Bruker Compass DataAnalysis 4.2 Page 1 of 1

Figure S30. HR-ESI MS of **1**.

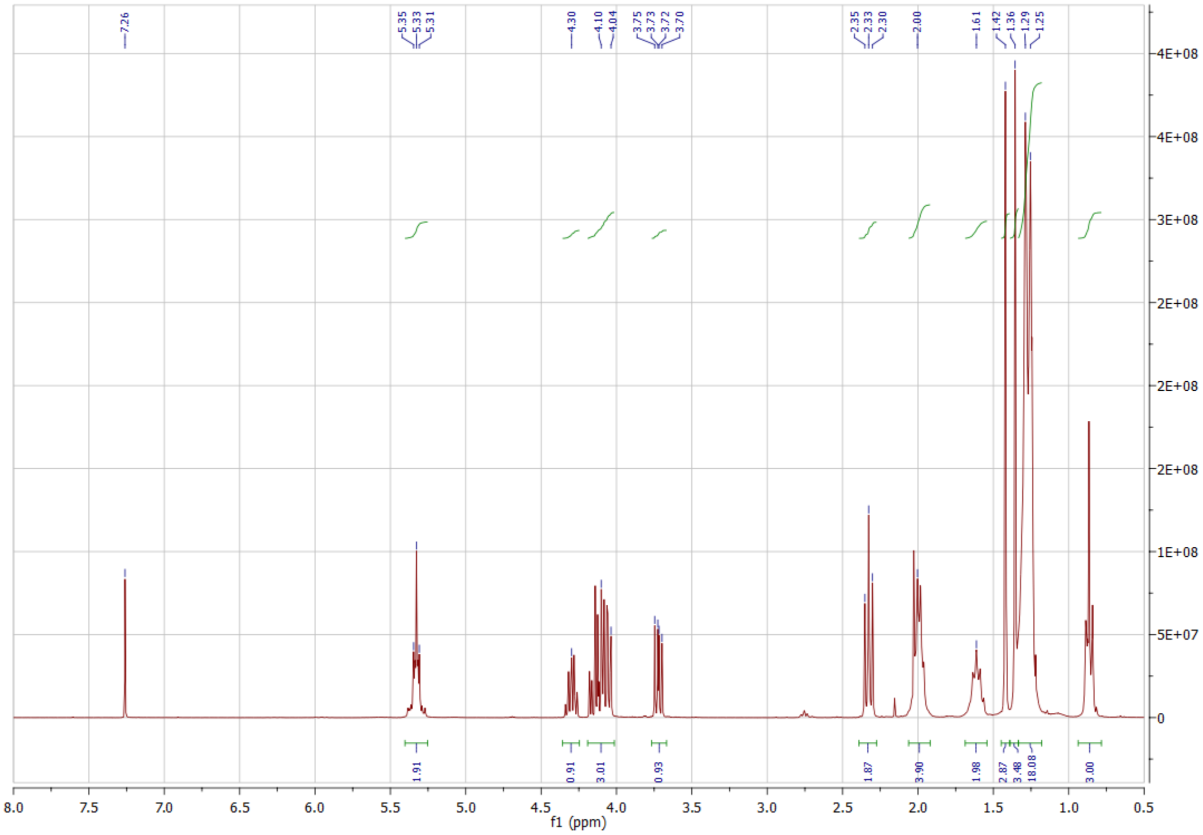

Figure S31. ^{1}H (300MHz, $CDCl_3$) of **11**.

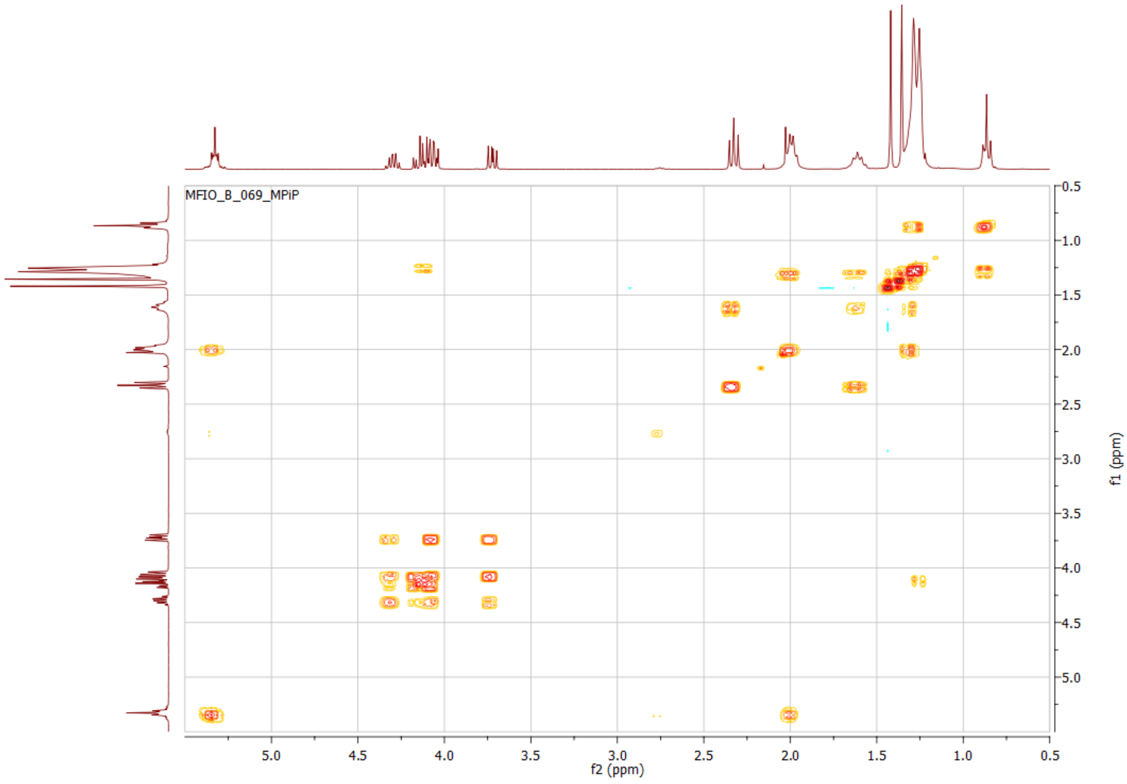

Figure S32. COSY (300MHz, $CDCl_3$) of **1.**

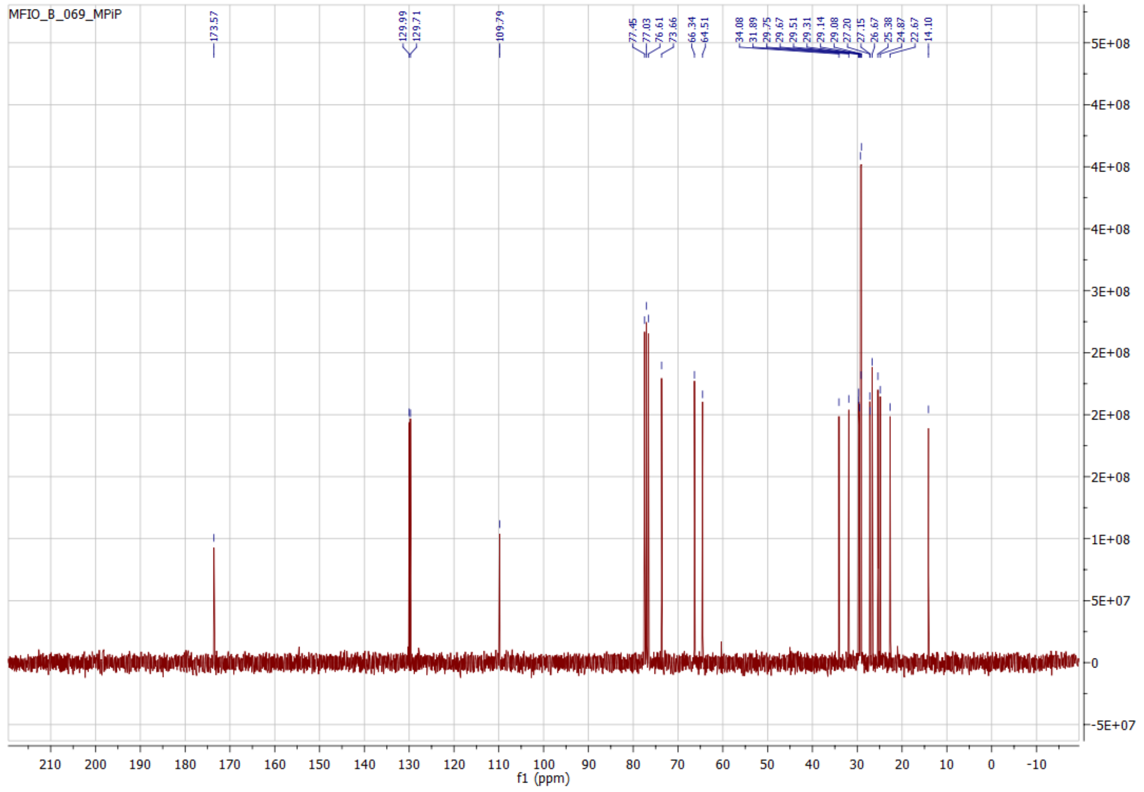

Figure S33. ^{13}C NMR (75MHz, $CDCl_3$) of **11**.

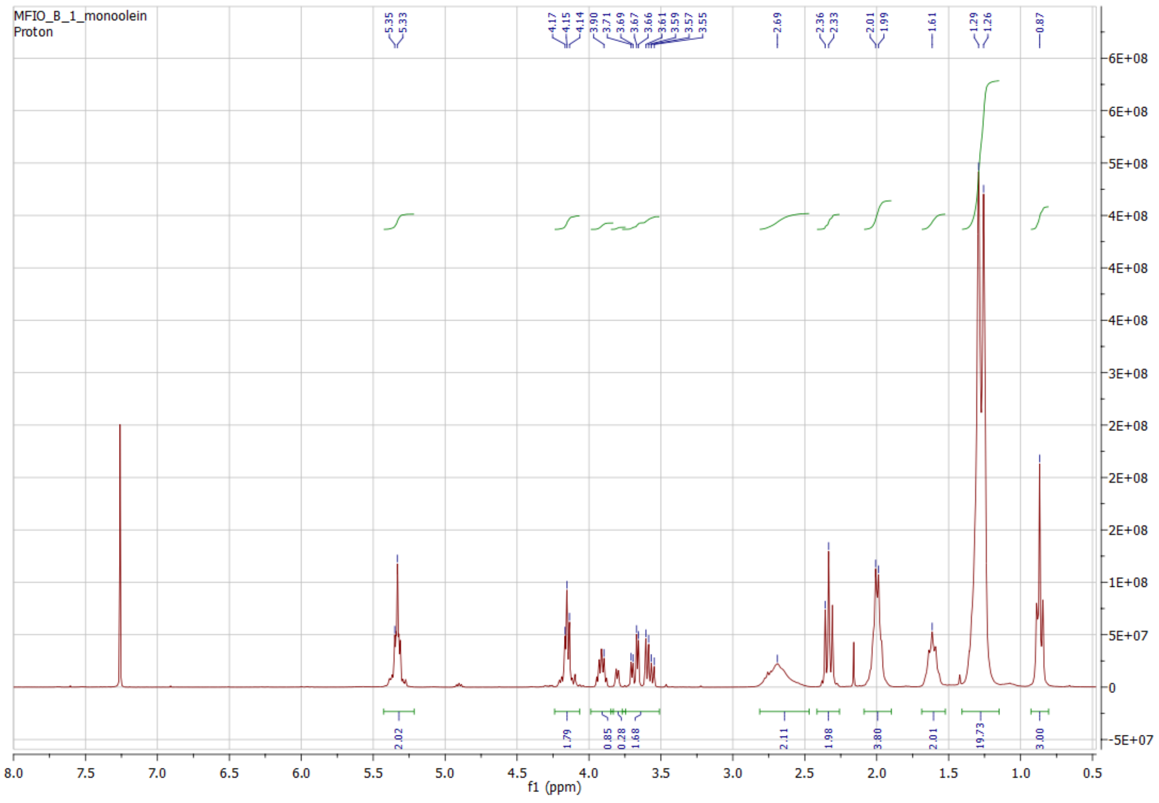

Figure S34. ^{1}H NMR (300MHz, $CDCl_3$) of **7a**.

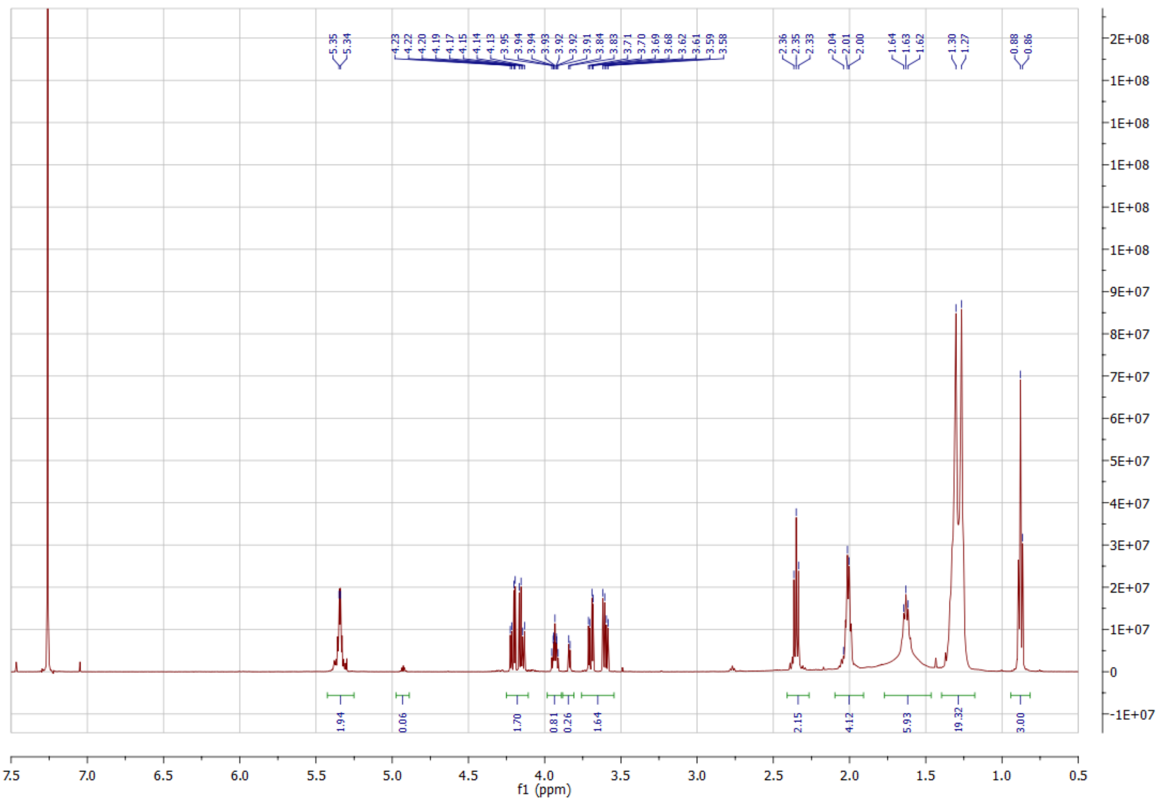

Figure S35. ^{1}H NMR (500MHz, $CDCl_3$) of **7a**.

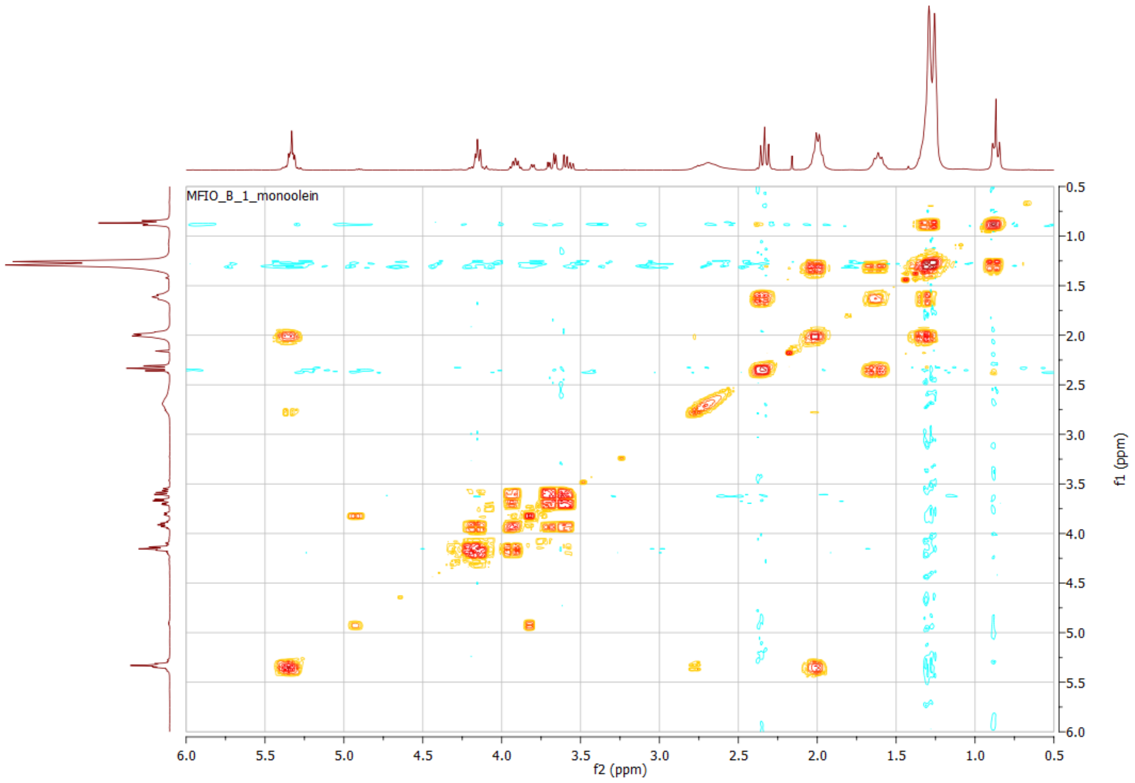

Figure S36. COSY (300MHz) of **7a**.

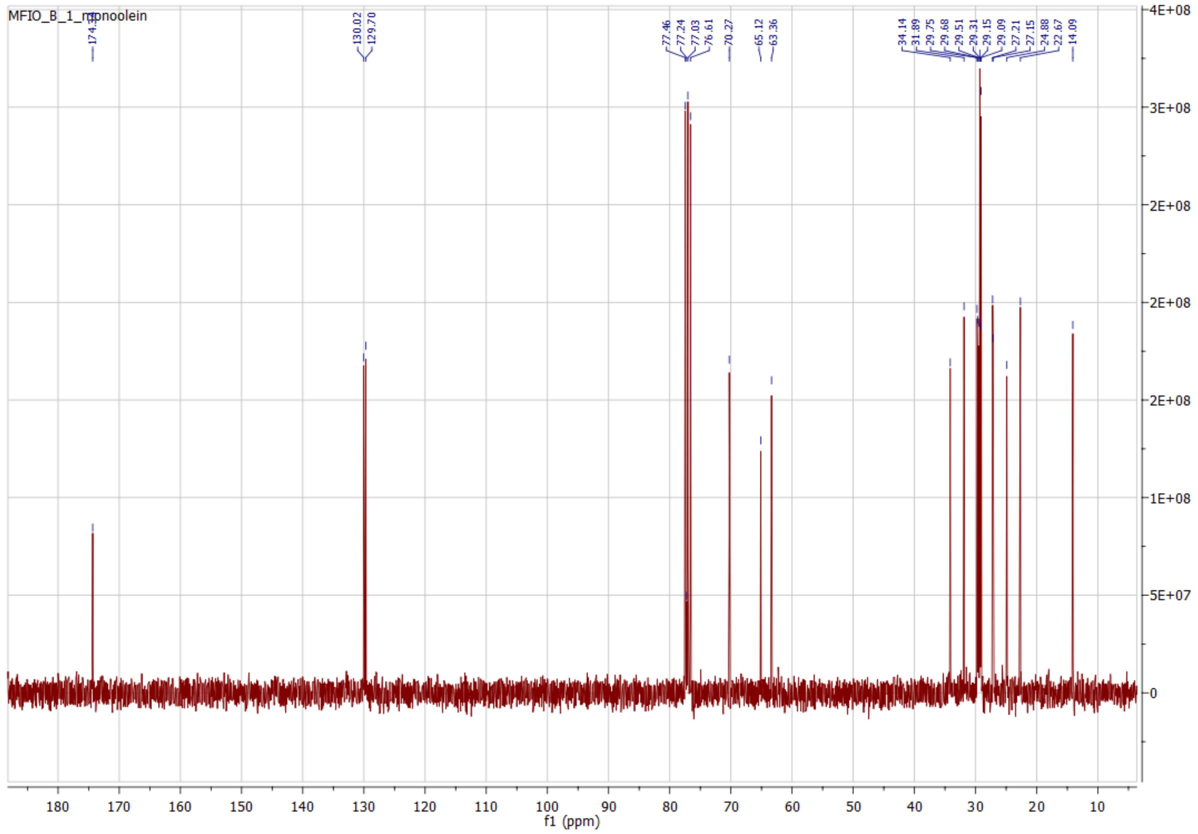

Figure S37. ^{13}C NMR (75 MHz, $CDCl_3$) of **7a**

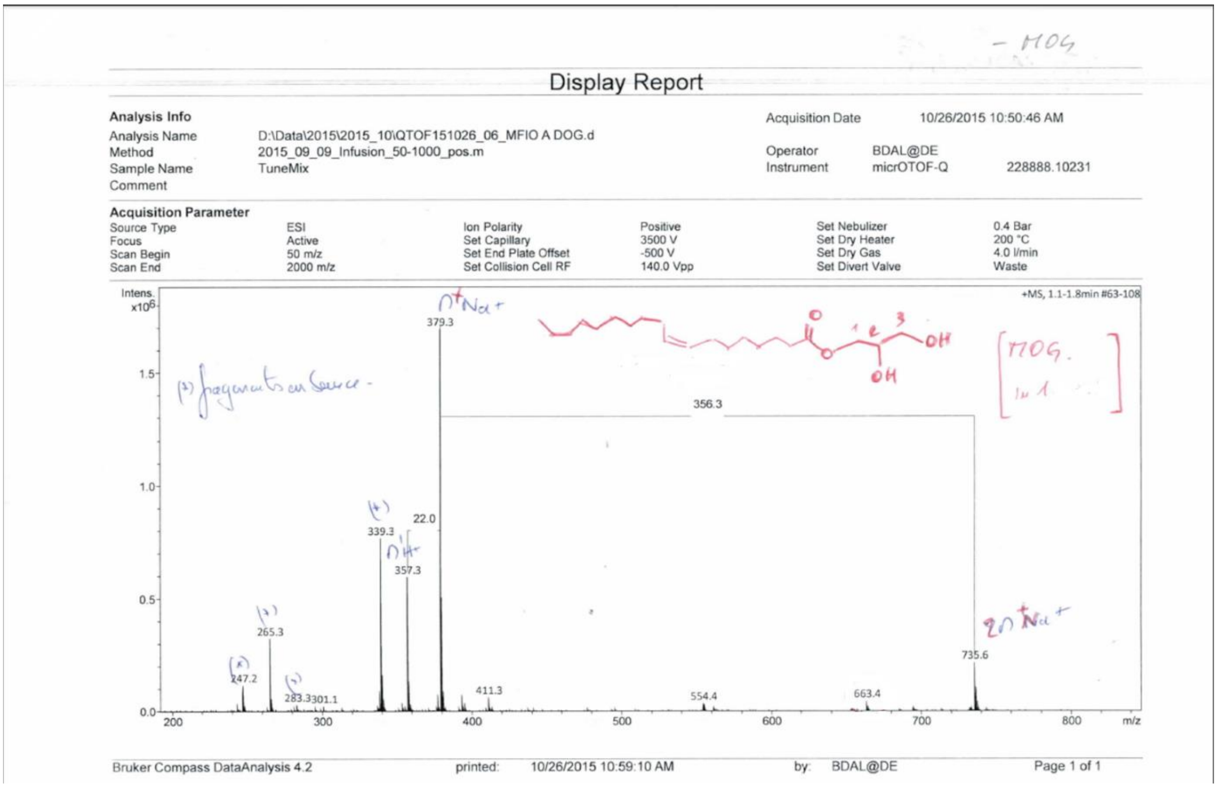

Figure S38. ESI–MS (positive ion mode) of **7a**.

VI Quantitative analysis of Mix A vesicles as derived from flow cytometry

Table S2. Quantitative analysis of Mix A vesicles as derived from flow cytometry

	**P1**<br>(low SSC)	**P2**<br>(high SSC)	Total
	81%	19%	100%
Normally filled (FITC < 10^4 a.u.)	79.5%<br>**p11**	17.8%<br>**p21**	97.3%
Highly filled (FITC > 10^4 a.u.)	1.5%<br>**p12**	1.2%<br>**p22**	2.7%
Highly filled (%)	1.8% (**p12/P1**)	6.3% (**p22/P2**)	

VII Additional confocal fluorescence micrographs of Mix A, M1–4 and M5

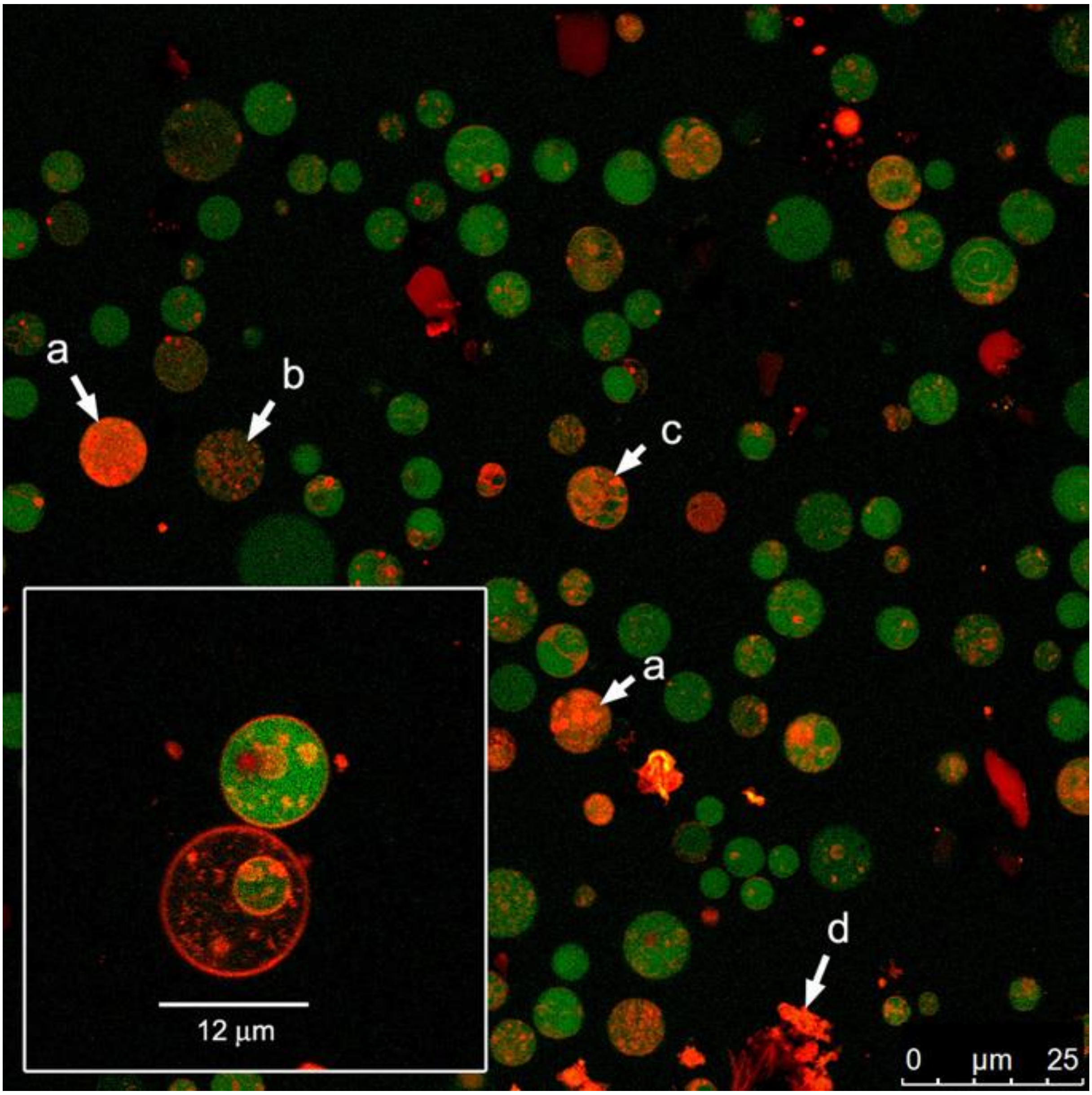

Figure S39. An additional image of GVs from Mix A hydration (in 5 mM bicine, pH 8.5; 200 mM sucrose inside/glucose outside), stained with DOPE-Rh (0.1 mol%). The red staining allows the detection of lipid-rich regions in the pictured particles. Calcein filled vesicles appear as a green circle surrounded by a thin red layer. In contrast, particles appearing red are lipid-rich. They might include quite large lipid particles (a), calcein-containing in the presence of captured small lipid particles or small vesicles (b), GVs with foam-like internal structure (c), or lipid clumps (d). In some cases GVs inside GVs (multivesicular vesicles or vesosomes) can be observed, as in the inset (note the different calcein-filling pattern).

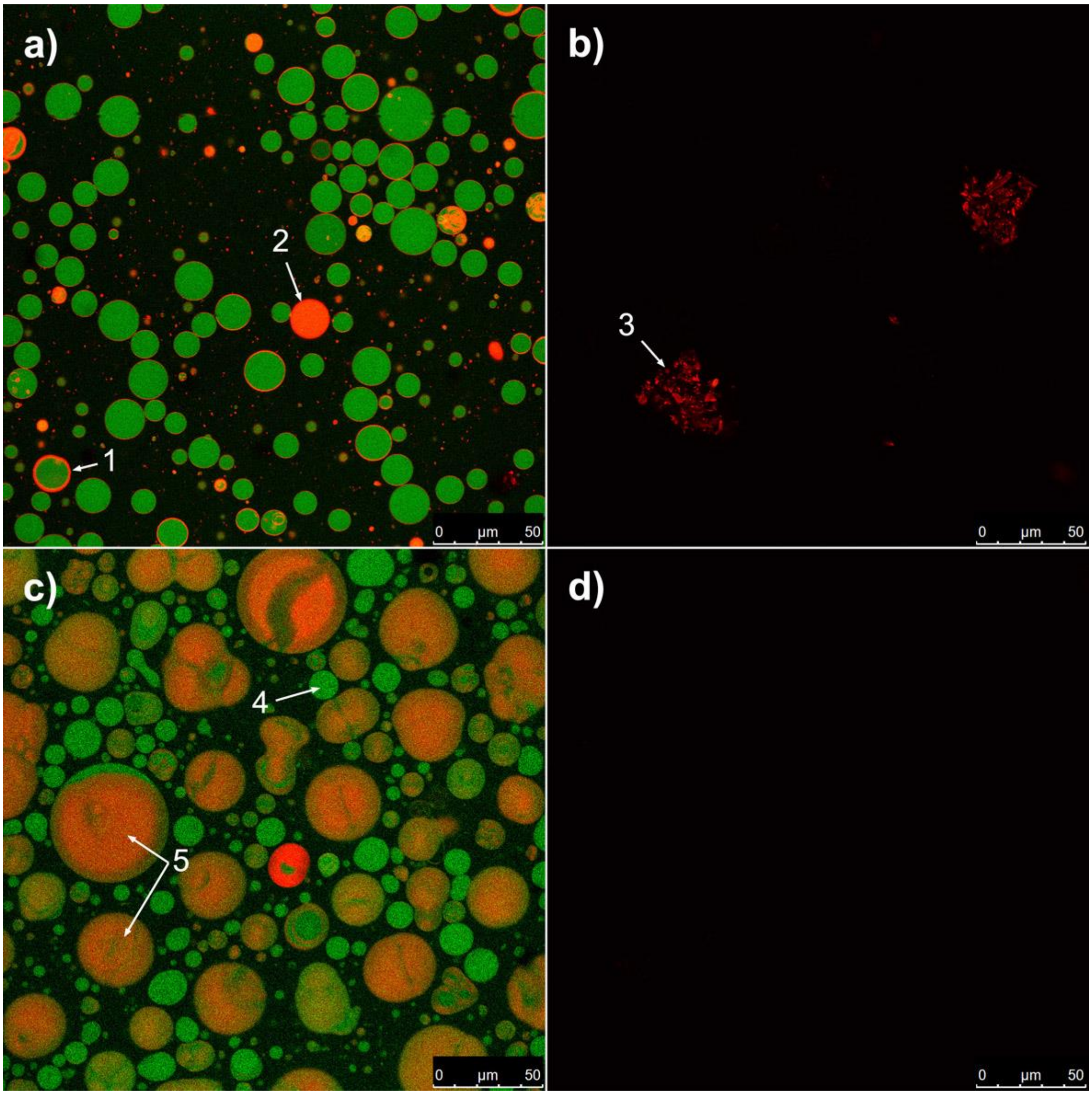

Figure S40. Pictures of GVs prepared by hydrating reconstituted mixtures M1 (a), M2 (b), M3 (c), M4 (d) (see Table 1 in the article). M1 and M3, which contain 30 and 60 mol% of DOPA, have been hydrated in the I-solution made of 25 mM Tris-HCl, 200 mM sucrose (pH 7.5). In contrast, M2 and M4, which contain 30 and 60 mol% of DOPE, have been hydrated with 200 mM Na-bicine, 200 mM sucrose (pH 8.5). Calcein-containing GVs are found in good amounts in samples M1 and M3, whereas the mixture remained essentially not hydrated for samples M2 and M4. Arrow 1 indicates a GVs which is probably multilamellar, as evident by its highly red fluorescence due to high amount of the lipid marker DOPE-Rh; arrow 2 indicates a lipid-filled spherical particle, probably surrounded by a lipid bilayer; arrow 3 indicates poorly hydrated lipid clumps; arrow 4 indicates a GVs in a sample which contain also lipid-rich particles, which appear red due to abundance of DOPE-Rh (arrow 5).

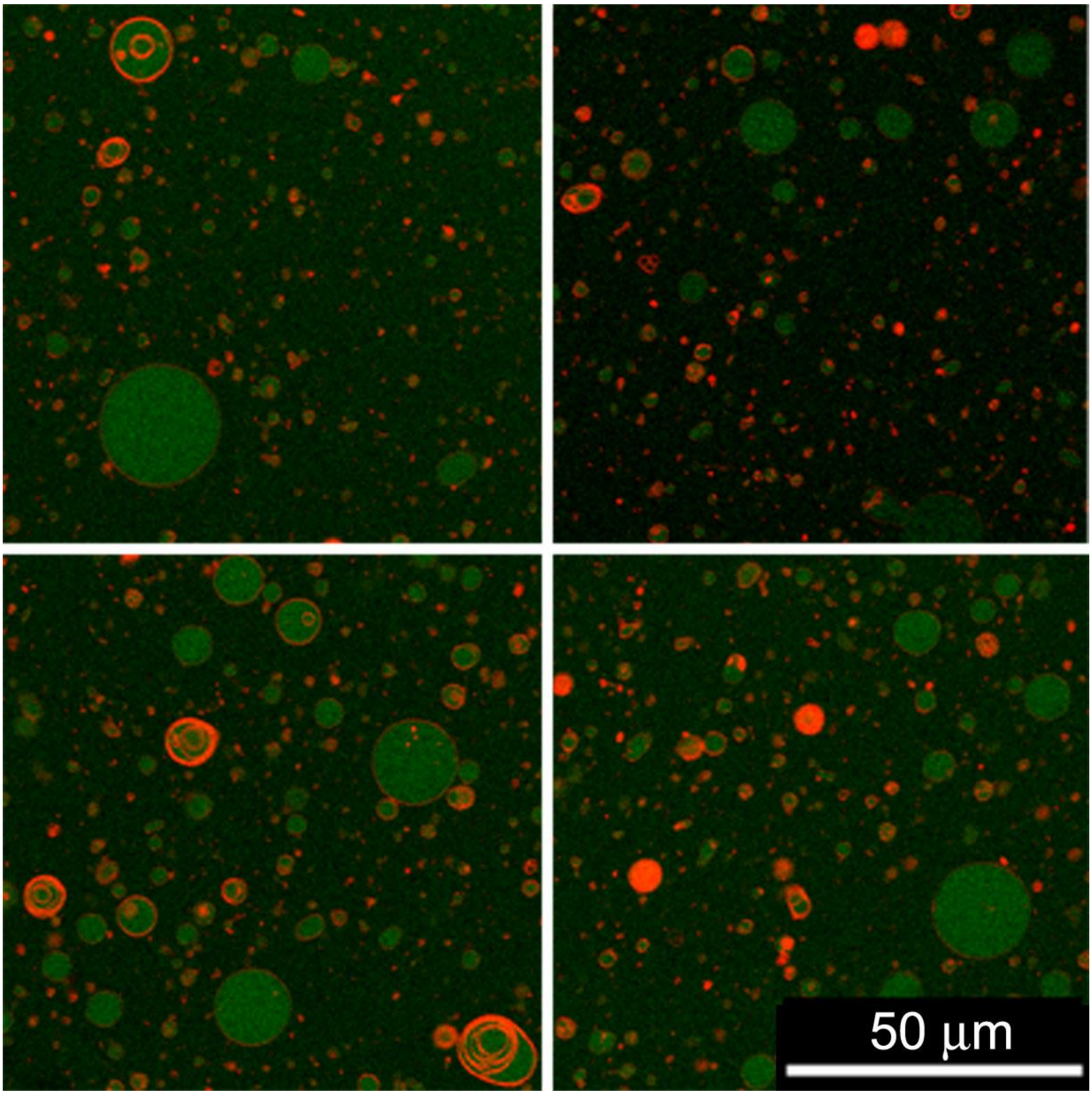

Figure S41. Pictures of GVs prepared from mixture M5 in 200 mM bicine pH 8.5; and 200 mM sucrose inside/200 mM glucose outside. Note that DOPC is 60 mol% (Table S3). GVs lumen appears green due to encapsulated calcein fluorescence, lipid membranes appear red due to the co-hydration of DOPE-Rh (0.02 % *w/v*).

Entry	Oleic Acid (**8**)	*rac*-MOG (**7a**)	*rac*-DOG (**1**)	DOPC	Vesicles size
M5	1	1	2	6	5-10 μm
